# Simulations reveal challenges to artificial community selection and possible strategies for success

**DOI:** 10.1371/journal.pbio.3000295

**Published:** 2019-06-25

**Authors:** Li Xie, Alex E. Yuan, Wenying Shou

**Affiliations:** 1 Basic Sciences Division, Fred Hutchinson Cancer Research Center, Seattle, Washington, United States of America; 2 Molecular and Cellular Biology PhD program, University of Washington, Seattle, Washington, United States of America; New York University, UNITED STATES

## Abstract

Multispecies microbial communities often display “community functions” arising from interactions of member species. Interactions are often difficult to decipher, making it challenging to design communities with desired functions. Alternatively, similar to artificial selection for individuals in agriculture and industry, one could repeatedly choose communities with the highest community functions to reproduce by randomly partitioning each into multiple “Newborn” communities for the next cycle. However, previous efforts in selecting complex communities have generated mixed outcomes that are difficult to interpret. To understand how to effectively enact community selection, we simulated community selection to improve a community function that requires 2 species and imposes a fitness cost on one or both species. Our simulations predict that improvement could be easily stalled unless various aspects of selection are carefully considered. These aspects include promoting species coexistence, suppressing noncontributors, choosing additional communities besides the highest functioning ones to reproduce, and reducing stochastic fluctuations in the biomass of each member species in Newborn communities. These considerations can be addressed experimentally. When executed effectively, community selection is predicted to improve costly community function, and may even force species to evolve slow growth to achieve species coexistence. Our conclusions hold under various alternative model assumptions and are therefore applicable to a variety of communities.

## Abstract 2

Simulations allow the authors to explore the feasibility of artificially selecting for functioning microbial communities, helping to establish why community selection might fail and how one could ensure that it works.

## Introduction

Multispecies microbial communities often display community functions, defined as biochemical activities not achievable by member species in isolation. Many community functions have important commercial values. For example, a 6-species microbial community—but not any member species alone—cleared relapsing *Clostridium difficile* infections in mice [1]. Community functions arise from interactions by which an individual alters the physiology of another individual. Thus, to improve community functions, one could identify and modify interactions [2,3]. In reality, this is no trivial task: each species can release dozens or more compounds, many of which may influence the partner species in diverse fashions [4,5,6,7]. From this myriad of interactions, one would then need to identify those critical for community function and modify them by altering species genotypes or the abiotic environment. One could also artificially assemble different combinations of species or genotypes to screen for high community function (e.g. [8, 9, 10]). However, some species may not be culturable in isolation. Moreover, the number of combinations becomes very large even when testing a moderate number of species and genotypes at various ratios, although recent advance has enabled massive parallel screening of synthetic microbial communities in droplets [11].

**Fig 1:**
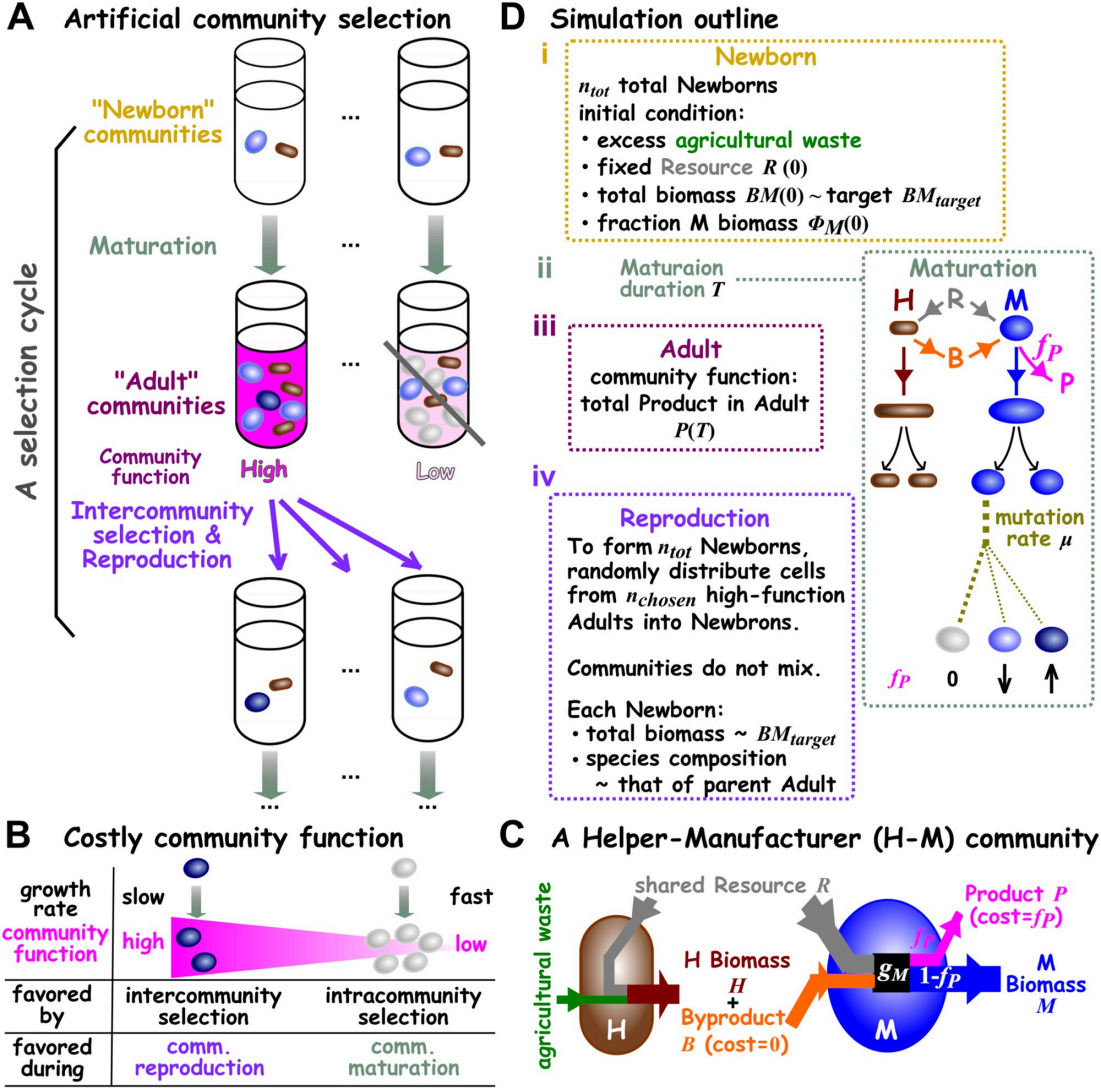
Community selection. (A) Schematic of artificial community selection. (B) Costly community function. Dark cells contribute more to community function per cell and thus divide more slowly than light cells. In other words, high contributors are disfavored by intracommunity selection during community maturation. However, communities dominated by high contributors are favored by intercommunity selection and have a higher chance to reproduce. (C) A Helper-Manufacturer community that converts substrates into a product. Helper H consumes agricultural waste and Resource R to grow biomass, and concomitantly releases Byproduct B at no fitness cost to itself. H’s Byproduct B is required by Manufacturer M. M consumes Resource and H’s Byproduct, and invests a fraction *f*_*P*_ of its potential growth *g*_*M*_ to make Product P while channeling the remaining to biomass growth. When biomass growth ceases, Byproduct and Product are no longer made. The 5 state variables (italicized) *H*, *M*, *R*, *B*, and *P* correspond to the amount of H biomass, M biomass, Resource, Byproduct, and Product in a community, respectively. Agricultural waste is present in excess, and thus does not enter equations. (D) Simulating artificial selection of H-M communities. (i) In our simulations, cycles of selection were performed on a total of *n*_*tot*_ = 100 communities with the indicated initial conditions. At the beginning of the first cycle, each Newborn had a total biomass of the target value (*BM*_*target*_ = 100; 60 M and 40 H each of biomass 1). In subsequent cycles, as dictated by experiments that we simulate, each Newborn’s total biomass would fluctuate around the target total biomass *BM*_*target*_, and each Newborn’s species ratio would fluctuate around its parent Adult’s species ratio. The amount of Resource in each Newborn was fixed at a value that could support a total biomass of 10^4^ (unless otherwise stated). (ii) The maturation time *T* was chosen so that, for an average community, Resource was not depleted by time *T* (in experimental terms, this would avoid complications of the stationary phase). During maturation, Resource *R*, Byproduct *B*, Product *P*, and each cell’s biomass were calculated from differential equations (Methods, Section 6). Once a cell’s biomass had grown from 1 to 2, it divided into 2 identical daughter cells. Death occurred stochastically to individual cells (not depicted). After division, mutations (different shades of oval) occurred stochastically to change a cell’s phenotypes (e.g., M’s *f*_*P*_). (iii) At the end of a cycle, community functions (total Product *P*(*T*)) were ranked. (iv) During community reproduction, high-functioning Adults were chosen and diluted into Newborns so that, on average, each Newborn had a total biomass of approximately the target biomass *BM*_*target*_. A total of *n*_*tot*_ = 100 Newborns were generated for the next selection cycle. In this study, communities never mixed with each other. Adult, Adult community; B, Byproduct; H, Helper; M, Manufacturer; Newborn, Newborn community; P, Product; R, Resource.

In an alternative approach, artificial selection of whole communities could be carried out over cycles to improve community trait [12–17, 115–120] (reviewed in [18,19,20]; Fig 1A). A selection cycle starts with a collection of low-density "Newborn" communities with artificially imposed boundaries (e.g., inside culture tubes). These low-density communities are incubated for a period of time ("maturation") to form "Adult" communities. During maturation, community members multiply and interact with each other and possibly mutate, and the community function of interest develops. At the end of maturation, desired Adult communities are chosen to “reproduce” such that each is randomly partitioned into multiple Newborn communities to start the next cycle. Superficially, this process may seem straightforward since "one gets what one selects for." After all, artificial selection on individuals has been successfully implemented to obtain, e.g., proteins of enhanced activities ([21,22,23]; S1 Fig). However, compared to artificial selection of individuals or monospecies groups, artificial selection of multispecies communities is more challenging (see detailed explanation in S1 Fig). For example, member species critical for community function may get lost during community reproduction.

The few attempts at community selection have generated interesting results. One theoretical study simulated artificial selection on multispecies communities based on the presence or absence of a member species [17]. Communities responded to selection, but only under certain conditions. In another theoretical study, multispecies communities responded to artificial selection for their ability to modify their abiotic environment in user-defined fashions [14]. In both cases, the response to selection quickly leveled off, and could be generated without mutations. Thus, community selection acted entirely on species types instead of new genotypes [14,17]. In experiments, complex microbial communities were selected for various traits [12,13,15,16,115,116]. For example, microbial communities selected to promote early or late flowering in plants were dominated by distinct species types [15]. However in other cases, a community trait may fail to improve despite selection and may improve even without selection [12,13,116].

Because communities used in these selection attempts were complex, much remains unknown. First, was the trait under selection a community function or achievable by a single species? If the latter, then community selection may not be needed, and the simpler task of selecting individuals or monospecies groups could be performed instead (S1 Fig). Second, did selection act solely on species types or also on newly arising genotypes? If selection acted solely on species types [14,15,17,118], then without immigration of new species to generate new variations, community function may quickly plateau [14,17,118]. If selection acted on genotypes, then community function could continue to improve as new genotypes evolve. Finally, why might a community trait sometimes fail to improve despite selection [12,13]?

In this study, we simulated artificial selection on communities with 2 defined species whose phenotypes can be modified by random mutations. Our goal is to improve a “costly” community function. A community function is costly if any community member’s fitness is reduced by contributing to that community function (Fig 1B). Costly community functions can arise when microbes are engineered to contribute. Costly community functions are particularly challenging to improve: because contributors to community function grow slower than noncontributors, noncontributors will take over during community maturation. If all Adult communities are dominated by noncontributors, then community selection will fail. To improve a costly community function, intercommunity selection (which occurs once every cycle) must overcome intracommunity selection throughout community maturation (Fig 1B).

By simulating a simplified 2-species community, we could compare the efficacy of different selection regimens with relative ease, unlike experimental comparisons that would require concerted efforts from multiple labs [24]. Additionally, by analyzing evolving communities—their functions and member species phenotypes—we could begin to understand evolutionary dynamics during community selection. We also designed our simulations to mimic real lab experiments so that our conclusions could guide future experiments. For example, our simulations incorporated not only chemical mechanisms of species interactions (as advocated by [25,26]) but also experimental procedures (e.g., pipetting cultures during community reproduction). Model parameters, including species phenotypes, mutation rate, and distribution of mutation effects, were based on a wide variety of published experiments. Note that most previous models (e.g., [121]) focused on binary phenotypes (e.g., either contributing or non-contributing) and therefore could not model community function improvement driven by the evolution of quantitative phenotypes. We show that artificial community selection can improve a costly community function, but only after circumventing a multitude of failure traps. In discussions, we will elaborate on (i) challenges and solutions to community selection; (ii) the tension between intracommunity selection versus intercommunity selection; (iii) similarities and dinstinctions among individual selection, group selection, and community selection; (iv) why optimizing monocultures may not lead to optimal community function; (v) implications of our work; and (vi) future directions.

## Results

We will first introduce the subject of our community selection simulation: a commensal 2-species community that converts substrates to a valued product. We will then define community function and describe how we simulate artificial community selection. Using simulation results, we will demonstrate critical measures that make community selection effective. Finally, we show that our conclusions are robust under alternative model assumptions, applicable also to mutualistic communities and communities whose member species may not coexist. To avoid confusion, we will use "community selection" or "selection" to describe the entire process of artificial community selection (community formation, growth, selection, and reproduction), and use "choose" or "intercommunity selection" to refer to the step in which the experimentalist decides which communities will reproduce.

### A Helper-Manufacturer community that converts substrates into a product

Motivated by previous successes in engineering 2-species microbial communities that convert substrates into useful products [27,28,29], we numerically simulated selection of such communities.

In our community, Manufacturer M can manufacture Product P of value to us (e.g., a biofuel or a drug) at a fitness cost to self, but only if assisted by Helper H (Fig 1C). Specifically, Helper but not Manufacturer can digest an agricultural waste (e.g., cellulose), and as Helper grows biomass, Helper releases Byproduct B at no fitness cost to itself. Manufacturer requires H’s Byproduct (e.g., carbon source) to grow. In addition, Manufacturer pays the cost of *f*_*P*_ (0 ≤ *f*_*P*_ ≤ 1) fraction of its potential growth to make Product P while using the rest (1 − *f*_*P*_) for its biomass growth. Both species also require a shared Resource R (e.g., nitrogen). Thus, the 2 species together—but not any species alone—can convert substrates (agricultural waste and Resource) into Product.

We define community function as the total amount of Product accumulated as a low-density Newborn community grows into an Adult community over maturation time *T*, i.e., *P*(*T*). In the Discussion section, we explain problems associated with an alternative definition of community function (e.g., per capita production; Methods Section 7; S2 Fig). We will initially focus on the scenario in which community function is not costly to Helpers but incurs a fitness cost of *f*_*P*_ to M. Later, we will show that our conclusions also hold when community function is costly to both H and M. Below, we will describe how we simulated community selection, followed by how we chose parameters of species phenotypes and parameters of selection regimen.

### Simulating community selection

We simulated 4 stages of community selection (Fig 1D) as follows: (i) forming Newborn communities, (ii) Newborn communities maturing into Adult communities, (iii) choosing high-functioning Adult communities, and (iv) reproducing the chosen Adult communities by splitting each into multiple Newborn communities of the next cycle. Our simulation was individual-based. That is, it tracked phenotypes and biomass of individual H and M cells in each community as cells grew, divided, mutated, or died. Our simulations also tracked dynamics of chemicals (including Product) in each community and accounted for actual experimental steps such as pipetting cultures during community reproduction. Below is a brief summary of our simulations, with more details in Methods (Section 6).

Each simulation started with *n*_*tot*_ (= 100) number of Newborn communities. Each Newborn community always started with a fixed amount of Resource *R*(0). Agricultural waste was always supplied in excess and thus did not enter our equations. In the first cycle, each Newborn community had a total biomass equal to a target value *BM*_*target*_ (= 100; 60 Manufacturers and 40 Helpers each of biomass 1). See Discussion for problems associated with not having a biomass target.

During community maturation, biomass of individual cells grew. The biomass growth rate of a Helper cell depended on Resource concentration (Monod equation; S3A Fig; Eq 23). As H grew, it consumed Resource and simultaneously released Byproduct (Eqs 21 and 22). The potential growth rate of a Manufacturer cell depended on the concentrations of Resource and H’s Byproduct (Mankad-Bungay dual-nutrient equation [30]; S3B Fig; see experimental support in S4 Fig). As M grew, it consumed Resource and Byproduct (Eqs 21 and 22) proportional to its potential growth rate. M grew biomass at (1 − *f*_*P*_) fraction of potential growth rate (Eq 24), and released Product at a rate proportional to *f*_*P*_ fraction of potential growth rate (Eq 25). Once an H or M cell’s biomass grew from 1 to 2, it divided into 2 cells of equal biomass with identical phenotypes, thus capturing experimental observations of continuous biomass increase (S5 Fig) and discrete cell division events [31]. At any time, H and M cells died stochastically at a constant death rate. Although mutations can occur during any stage of the cell cycle, we assigned mutations immediately after cell division, such that each mutable phenotype of both cells mutated independently.

Mutable phenotypes included H and M’s "growth parameters" (maximal growth rates in excess nutrients; affinities for nutrients), and M’s cost *f*_*P*_. These phenotypes have been observed to rapidly change during evolution [32,33,34,35]. Mutated phenotypes could range between 0 and their respective evolutionary upper bounds. Among mutations that alter phenotypes (denoted "mutations"), on average, half abolished the function (e.g., zero growth rate, zero affinity, or zero cost *f*_*P*_) based on experiments on green fluorescent protein, viruses, and yeast [36,37,38]. Effects of the other 50% of mutations were bilateral-exponentially distributed, enhancing or diminishing a phenotype by a few percent, based on our reanalysis of published yeast data sets (S6 Fig) [39]. We held death rates constant, because death rates were much smaller than growth rates and therefore mutations in death rates would be inconsequential. We also held release and consumption coefficients constant. This is because, e.g., the amount of Byproduct released per H biomass generated is constrained by biochemical stoichiometry.

At the end of community maturation time *T*, we compared community function *P*(*T*) (the total amount of Product accumulated in the community by time *T*) for each Adult community. We chose high-functioning Adults to reproduce. Each chosen Adult was randomly partitioned into Newborns with target total biomass *BM*_*target*_. For example, if the chosen Adult had a total biomass of 60*BM*_*target*_, then each cell would be assigned a random integer between 1 and 60, and those cells with the same random integer would be allocated to the same Newborn. Experimentally, this is equivalent to volumetric dilution using a pipette ("pipetting"). Thus, for each Newborn, the total biomass and species ratio fluctuated around their expected values in a fashion associated with pipetting (Methods Section 9). From top-functioning Adults, a total of *n*_*tot*_ Newborns were obtained to enter the next selection cycle (see below for details).

### Choosing species: Enhancing species coexistence

In order to improve community function through community selection, species should ideally coexist throughout selection cycles. That is, all species should grow at a similar average growth rate within each cycle. Furthermore, species ratio should not be extreme because otherwise, the low-abundance species could be lost by chance during Newborn formation. Species coexistence at a moderate ratio has been experimentally realized in engineered communities [27,28,40,41].

**Fig 2:**
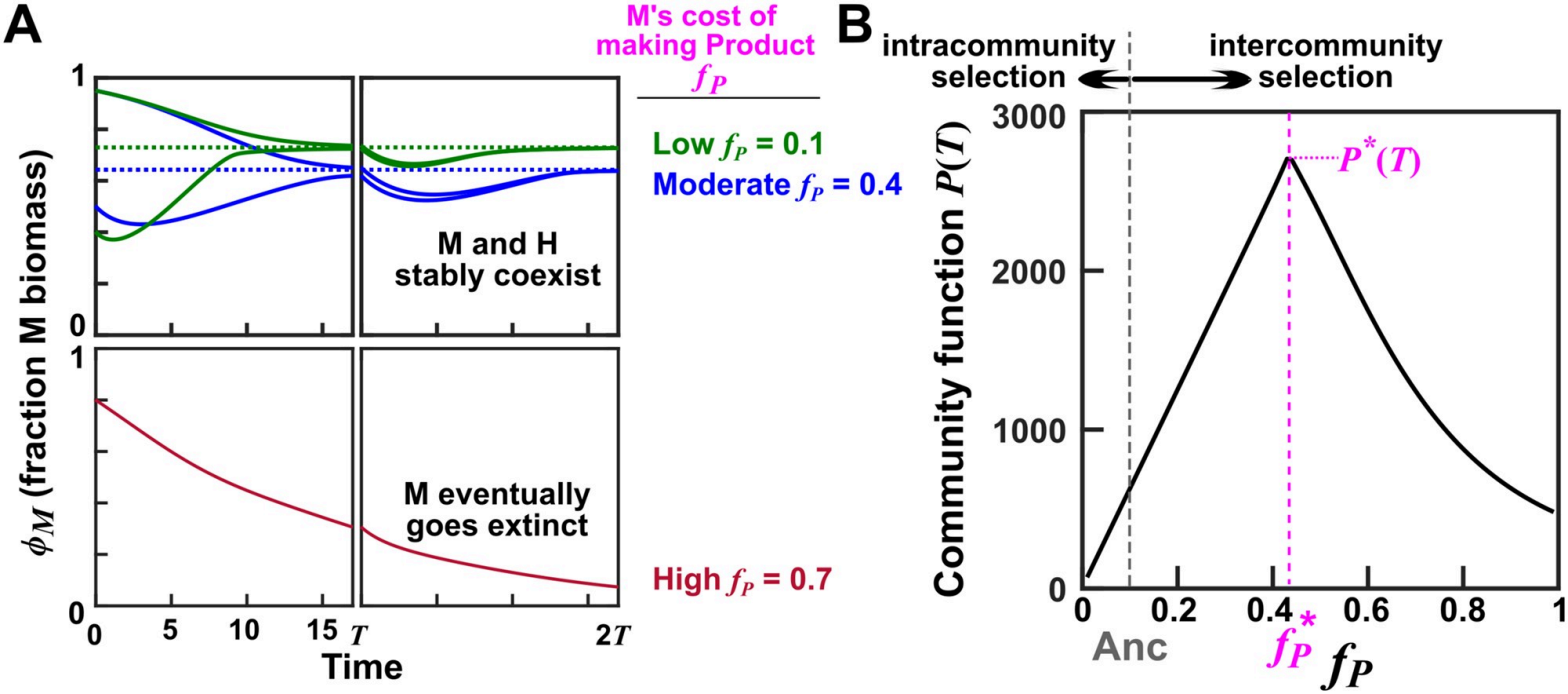
Species coexistence and optimal community function at an intermediate cost. Calculations were based on Eqs 6–10 with H and M’s growth parameters fixed at their respective evolutionary upper bounds (Table 1, “Preadapted”). (A) Stable species coexistence at moderate to low cost. Bottom: When *f*_*P*_, the fraction of potential growth Manufacturer diverts for making Product, is high (e.g., *f*_*P*_ = 0.7), M will eventually go extinct (i.e., fraction of M < 1 ÷ *BM_target_*). Top: At moderate and low cost *f*_*P*_ (e.g., *f*_*P*_ = 0.4 and *f*_*P*_ = 0.1), H and M can stably coexist. That is, different initial species ratios will converge to a steady-state value. At the end of the first cycle (time *T* = 17), Byproduct and Resource were reset to the initial conditions at time zero (0 and 10^4^, respectively), and total biomass was reduced to the target value *BM*_*target*_ = 100, while the fraction of M biomass *ϕ*_*M*_ remained the same as that of the parent community. See main text for how values of maturation time and Resource were chosen. (B) Optimal community function occurs at an intermediate cost *f*_*P*_. Community functions at various combinations of *f*_*P*_ and fraction of M biomass (out of *BM*_*target*_ = 100 total biomass) were computed by integrating Eqs 6–10. Maximal community function *P*(*T*) is achieved at an intermediate cost $f_{P}^{*}=0.41$ (magenta dashed line) when Newborn species composition is also optimal (46 H and 54 M cells). Note that, at zero *f*_*P*_, no Product would be made; at *f*_*P*_ = 1, M would go extinct. The maximal P*(*T*) could not be further improved even if we allowed all growth parameters and *f*_*P*_ to mutate (S10 Fig). Thus, P*(*T*) is locally maximal in the sense that small deviation will always reduce *P*(*T*). Ancestral *f*_*P*_ (gray) is lower than $f_{P}^{*}$. The central question is this: can intercommunity selection improve ancestral *f*_*P*_ to $f_{P}^{*}$ despite intracommunity selection favoring lower *f*_*P*_? The Matlab code can be found in S1 Code. H, Helper; M, Manufacturer.

To achieve species coexistence at a moderate ratio in the H-M community, three considerations need to be made. First, M’s cost *f*_*P*_ for making Product must not be too large, otherwise M would always grow slower than H and thus eventually go extinct (Fig 2A, bottom). Second, H and M’s growth parameters (maximal growth rates in excess nutrients; affinities for nutrients) must be balanced. This is because, upon Newborn formation, H can immediately start to grow on agricultural waste and Resource, whereas M cannot grow until H’s Byproduct has accumulated to a sufficiently high level. Thus, to achieve coexistence, M must grow faster than H at some point during community maturation. Third, to achieve a moderate steady-state species ratio, metabolite release and consumption need to be balanced [40]. Otherwise, the ratio between releaser and consumer can be extreme.

Based on these considerations and published measurements on *Saccharomyces cerevisiae* and *Escherichia coli*, we chose H and M’s ancestral growth parameters and their evolutionary upper bounds, as well as release, consumption, and death parameters (Table 1, Methods Section 2 and Section 3). Our choice of parameters ensured that throughout evolution, different species ratios would converge toward a moderate steady-state value during community maturation (Fig 2A, top). Note that if species were not chosen properly, selection might fail due to insufficient species coexistence (Fig 6A), although we will demonstrate that under effective community selection, requirements on species coexistence could be relaxed (Fig 6B–6D).

### Choosing selection regimen parameters: Avoiding known failure modes

After choosing member species with appropriate phenotypes, we need to consider the parameters of our selection regimen (Fig 1D). These parameters include the total number of communities under selection (*n*_*tot*_), the number of Adult communities chosen to reproduce (*n*_*chosen*_, the “bottleneck size” when choosing a fraction of Adults to reproduce), Newborn target total biomass (*BM*_*target*_, the "bottleneck size" when splitting an Adult into Newborns), the amount of Resource added to each Newborn (*R*(0)), the amount of mutagenesis that controls the rate of phenotype-altering mutations (*μ*), and maturation time (*T*). Compared to the well-studied problem of group selection in which the unit of selection is a monospecies group [42–56], community selection is more challenging (Discussion; S1 Fig). However, the 2 types of selections do share some common aspects (Discussion; S1 Fig). Thus, we can apply group selection theory, together with other practical considerations, to better design community selection regimen.

Let’s begin by considering the total number of communities under selection. The highest community function achieved among a large number of communities will likely exceed that achieved among a small number of communities. However, experimentally achieving a very large number of communities can be challenging. We chose a total of 100 communities (*n*_*tot*_ = 100).

*n*_*chosen*_, the number of Adults chosen by the experimentalist to reproduce, reflects intercommunity selection strength. Since the top-functioning Adult is presumably the most desirable, we reproduced it into as many Newborns as possible and then reproduced the second best, etc., until we obtained *n*_*tot*_ Newborn communities for the next cycle (the "top-dog" strategy). In most simulations, the top-functioning community contributed approximately 60–70 Newborns. We then reproduced the next highest-functioning Adult in the same way and randomly chose enough (approximately 30–40) Newborns so that a total of *n*_*tot*_ = 100 Newborns were generated for the next selection cycle. Later, we will compare the top-dog strategy with other strategies employing weaker intercommunity selection strengths.

Whether we mix chosen Adults before splitting them into Newborns could impact the efficacy of community selection. In our simulations, chosen Adults were not mixed to limit the spread of noncontributors. In other words, we created a spatially-structured environment, which is known to discourage noncontributors [57–60].

If the mutation rate is very low, then community function cannot rapidly improve. If the mutation rate is very high, then noncontributors will be generated at a high rate, and as the fast-growing noncontributors take over during community maturation, community function will likely collapse. Here, we chose *μ*, the rate of phenotype-altering mutations, to be biologically realistic (0.002 per cell per generation per phenotype, which is lower than the highest values observed experimentally; Methods Section 4).

If Newborn target total biomass *BM*_*target*_ is very large, or if the number of doublings within maturation time *T* is very large, then noncontributors will take over in all communities during maturation (S7 Fig, compare B-D with A), as predicted by group selection theory. On the other hand, if *BM*_*target*_ and the number of generations within *T* are both very small, then mutations will be rare within each cycle, and many cycles will be required to improve community function. Finally, if *BM*_*target*_ is very small, then a member species might get lost by chance during Newborn formation. In our simulations, we chose Newborn’s target total biomass *BM*_*target*_ = 100 biomass (approximately 50 to 100 cells). Unless otherwise stated, we fixed the input Resource *R*(0) to support a maximal total biomass of 10^4^ and chose maturation time *T* so that even if H and M had evolved to grow as fast as possible, total biomass would undergo approximately 6 doublings (increasing from about 100 to about 7,000). Thus, by the end of *T*, ≤ 70% Resource would be consumed by an average community. This meant that when implemented experimentally, we could avoid complications of Resource depletion and stationary phase while not wasting too much Resource.

### Community selection may not be effective under conditions reflecting common lab practices

**Fig 3:**
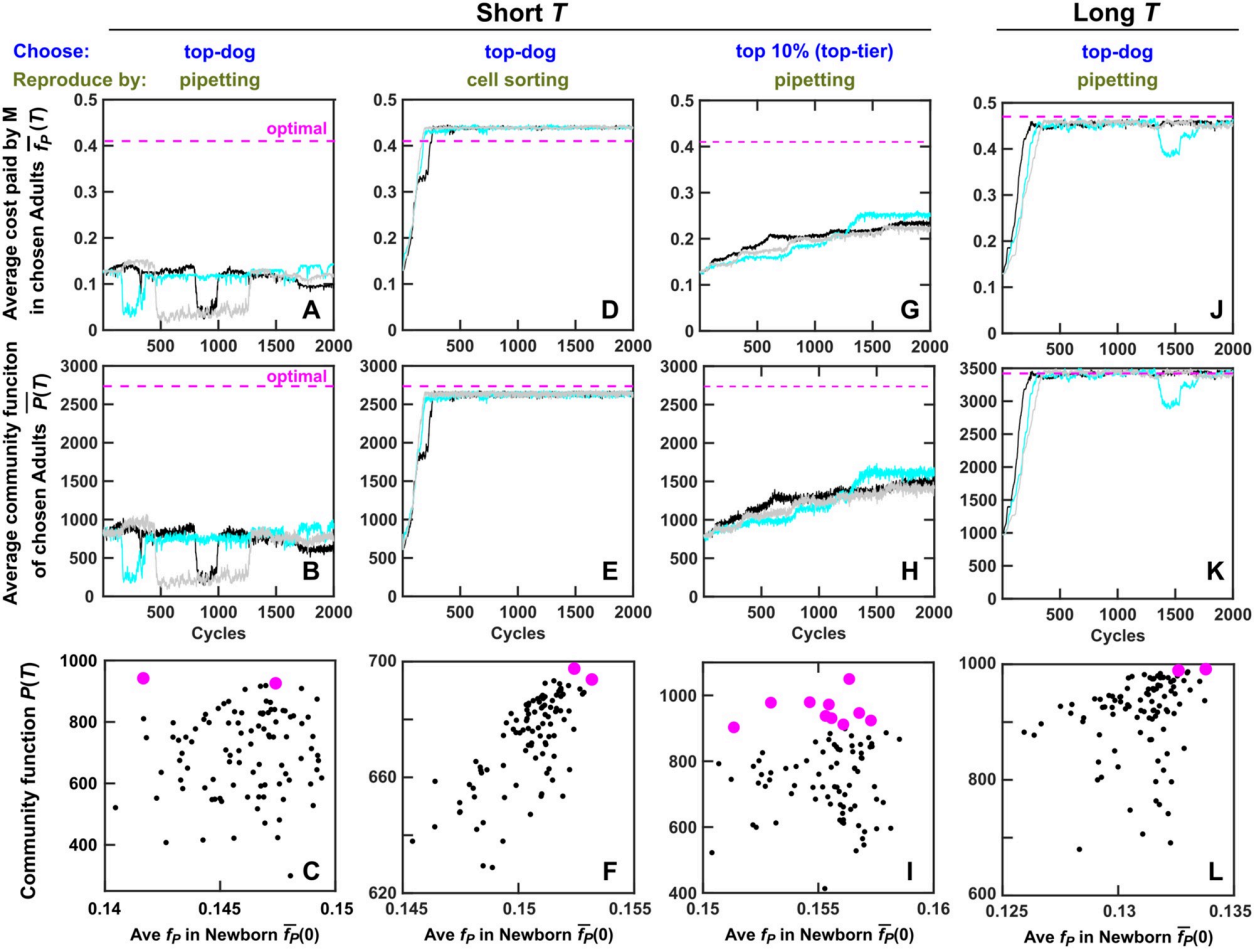
Community selection can be stalled by routine experimental procedures, and can succeed when community function correlates with its heritable determinant or when using the top-tier strategy. (A–I) Evolution dynamics when the maturation time *T* was sufficiently short to avoid Resource depletion and stationary phase (*T* = 17). (A–C) Adults were chosen using the top-dog strategy and diluted into progeny Newborns as if via pipetting (i.e., H and M biomass fluctuated around their expected values). Community selection was not effective: Average *f*_*p*_ and community function failed to improve to their theoretical optima. Community function poorly correlated with its heritable determinant ${\bar{f}}_{P}(0)$ (the average cost paid by M in Newborn). Black and magenta dots: unchosen and chosen communities from 1 selection cycle, respectively. (D–F) Adults were chosen using the top-dog strategy. A fixed H biomass and a fixed M biomass from a chosen Adult were allocated into each progeny Newborn as if using a cell sorter. Community selection was successful. Community function also correlated with its heritable determinant ${\bar{f}}_{P}(0)$. Here, Newborn total biomass *BM*(0) and fraction of M biomass *ϕ*_*M*_(0) were, respectively, fixed to *BM*_*target*_ = 100 and *ϕ*_*M*_(*T*) of the parent Adult of the previous cycle. (G–I) When we chose the top 10% Adults and let each reproduce 10 Newborns as if via pipetting, community function improved somewhat despite poor correlation between community function and its heritable determinant ${\bar{f}}_{P}(0)$. For selection dynamics over many cycles, see S14 Fig. (J–L) Evolution dynamics when maturation time was long (*T* = 20) such that, by the end of *T*, most Resource was consumed (stationary phase). Adults were chosen using the top-dog strategy and reproduced as if via pipetting. Community selection was successful due to high correlation between community function and its heritable determinant ${\bar{f}}_{P}(0)$, assuming that variable time in stationary phase would not introduce nonheritable variations in community function. Black, cyan, and gray curves represent independent simulation trials. $\bar{P}(T)$ was the average of *P*(*T*) across all chosen Adults. ${\bar{f}}_{P}(T)$ was obtained by first averaging among M within each chosen Adult and then averaging across all chosen Adults. The simulation codes can be found in S2 Code, and the data can be found in S1 Data. Adult, Adult community; H, Helper; M, Manufacturer; Newborn, Newborn community.

In initial simulations, we only allowed M’s cost *f*_*p*_ to be modified by mutations, and we fixed H and M’s growth parameters (maximal growth rates in excess metabolites; affinities for metabolites) to their evolutionary upper bounds. Such a simplification is justified with our particular parameter choices (Table 1) for the following reasons. First, we obtained qualitatively similar conclusions regardless of whether we fixed growth parameters or not (e.g., compare final community functions in Fig 3B and 3E versus S8A and S8D Fig). Second, when growth parameters mutated during community selection, they improved to their evolutionary upper bounds anyways (S8C and S8F Fig). Later, we will show an opposite case in which growth parameters were kept below their evolutionary upper bounds by effective community selection (Fig 6). A great advantage can be gained by allowing only cost *f*_*p*_ to mutate: we can now calculate the theoretical maximal community function P*(*T*) and its associated optimal cost $f_{P}^{*}\left(=0.41\right)$ and optimal species ratio at a fixed total Newborn biomass (Fig 2B).

We started simulations with M’s cost *f*_*p*_ lower than the optimal value $f_{P}^{*}$. Could intercommunity selection increase *f*_*p*_ to $f_{P}^{*}$, despite intracommunity natural selection favoring lower *f*_*p*_?

As expected, in control simulations in which Adult communities were randomly chosen to reproduce, community function was driven to zero by intracommunity natural selection as fast-growing nonproducing M took over (S9 Fig).

When we chose Adults using the top-dog strategy (starting from the top-functioning Adult followed by the runners-up) and split them into Newborns as if via pipetting, M’s cost *f*_*p*_ and community function *P*(*T*) did not decline to zero but they barely improved, and both were far below their theoretical optima (Fig 3A and 3B).

### Common lab practices can generate sufficiently large nonheritable variations in community function that interfere with selection

Why did community selection fail to increase M’s cost *f*_*P*_ and community function? One possibility is that community function was not sufficiently heritable from one cycle to the next (S1 Fig). We therefore investigated the heredity of community function by examining the heredity of community function determinants.

Community function *P*(*T*) was largely determined by phenotypes of cells in the Newborn community. This is because maturation time was sufficiently short (approximately 6 doublings), and therefore newly arising genotypes could not rise to high frequencies within one cycle to significantly affect community function. Because all phenotypes except for *f*_*P*_ were fixed, community function had 3 independent determinants: Newborn’s total biomass *BM*(0), Newborn’s fraction of M biomass *ϕ*_*M*_(0), and Newborn’s average M cost ${\bar{f}}_{P}(0)$ (*f*_*P*_ averaged across all M cells in the Newborn). Note that the first 2 values can stochastically fluctuate as expected from pipetting; e.g., to pipette 100 cells, one can end up pipetting between 70 and 130 cells even with a precise pipette.

**Fig 4:**
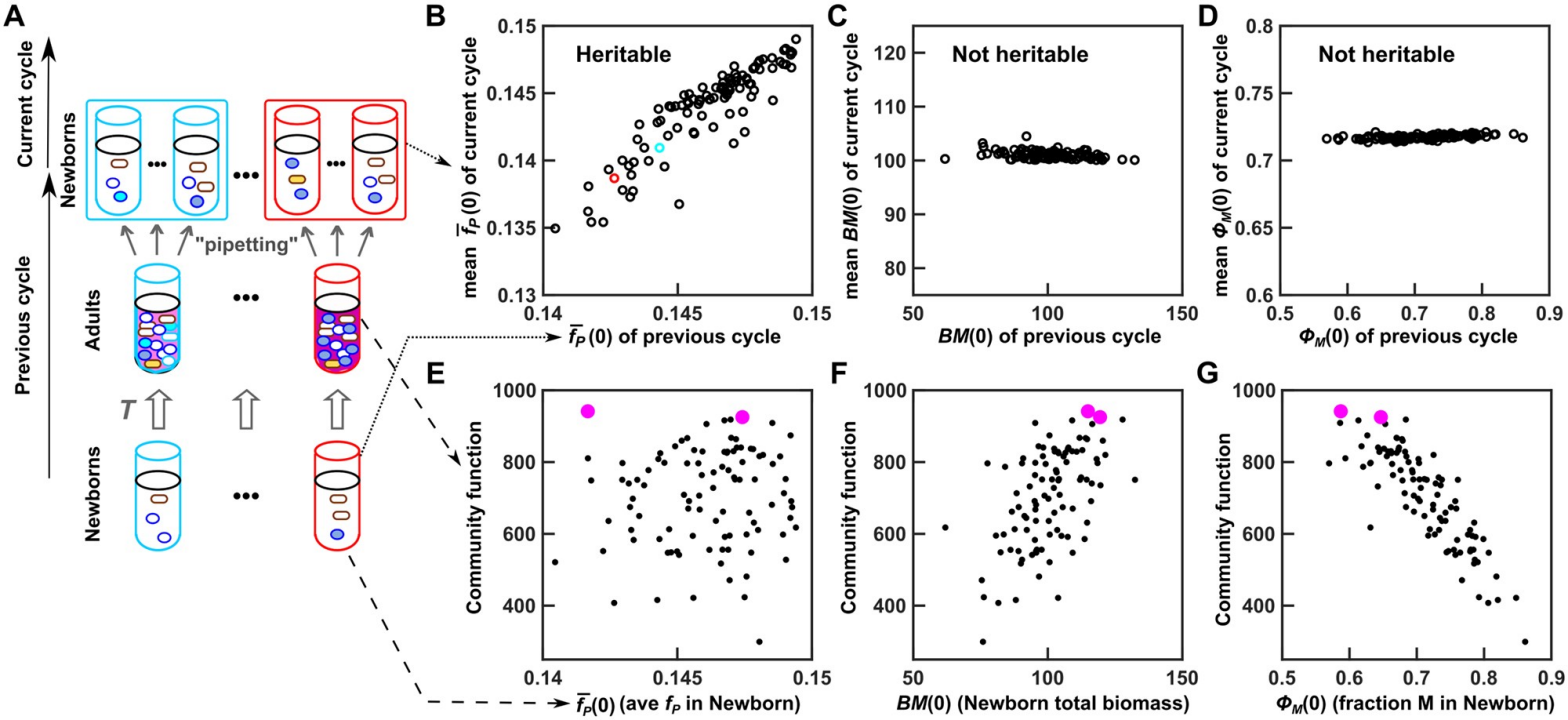
During ineffective community selection, community function correlates weakly with its heritable determinant and strongly with nonheritable determinants. (A) Schematic of community lineages across “previous” and “current” selection cycles. (B-G) Data of Newborns and corresponding Adults (previous cycle) were taken from the 180th cycle of the simulation displayed in black in Fig 3A and 3B. We then allowed each Adult to reproduce Newborns (current cycle), forming 100 lineages (tubes with the same color outline belong to the same lineage). (B–D) Among the 3 determinants of community function, ${\bar{f}}_{P}(0)$ (*f*_*P*_ averaged among M cells in Newborn) is heritable, but *BM*(0) (total biomass of Newborn) and *ϕ*_*M*_(0) (fraction of M biomass in Newborn) are not. For each lineage, the community function determinant at the previous cycle was scatter plotted against the average value at the current cycle. (E–G) During ineffective community selection (Fig 3B), community function *P*(*T*) correlates weakly with heritable determinant but strongly with nonheritable determinants. Each dot represents one community. Magenta dots: "successful" Newborns that achieved the highest community function at adulthood and therefore were chosen to reproduce in the top-dog strategy. The Matlab code for B–D can be found in S2 Code, and the data for E–G can be found in S1 Data. Adult, Adult community; M, Manufacturer; Newborn, Newborn community.

A community function determinant is considered heritable if it is correlated between Newborns of one cycle (Fig 4A, bottom row) and their respective progeny Newborns in the next cycle (Fig 4A, color-matched top row). Among the 3 determinants, ${\bar{f}}_{P}(0)$ was heritable (Fig 4B): if a Newborn community had a high average *f*_*P*_, so would the mature Adult community and Newborn communities reproduced from it. On the other hand, Newborn total biomass *BM*(0) was not heritable (Fig 4C). This is because when an Adult community reproduced via pipette dilution, the dilution factor was adjusted so that the total biomass of a progeny Newborn community was, on average, the target biomass *BM*_*target*_. Newborn’s fraction of M biomass *ϕ*_*M*_(0), which fluctuated around *ϕ*_*M*_(*T*) of its parent Adult, was not heritable either (Fig 4D). This is because, regardless of the species composition of Newborns, Adults would have similar steady-state species composition (Fig 2A top panel), and so would their offspring Newborns.

In successful community selection, variations in community function should be mainly caused by variations in its heritable determinants. However, community function *P*(*T*) weakly correlated with its heritable determinant ${\bar{f}}_{P}(0)$ but strongly correlated with its nonheritable determinants (Fig 4E–4G). For example, the Newborn that would achieve the highest function had a below-median ${\bar{f}}_{P}(0)$ (left magenta dot in Fig 4E) but had high total biomass *BM*(0) and low fraction of M biomass *ϕ*_*M*_(0) (Fig 4F and 4G). In other words, variation in community function is largely nonheritable, because it largely arises from variation in nonheritable determinants.

The reason for strong correlations between community function and its nonheritable determinants became clear by examining community dynamics. Recall that to avoid stationary phase, we had chosen maturation time so that Resource would be in excess by the end of maturation. Thus, a "lucky" Newborn community starting with a higher-than-average total biomass would convert more Resource to Product (dotted lines in top panels of S11 Fig). Similarly, if a Newborn started with higher-than-average fraction of Helper H biomass, then H would produce higher-than-average Byproduct, which meant that M would endure a shorter growth lag, grow more, and make more Product (dotted lines in bottom panels of S11 Fig).

To summarize, when community function significantly correlated with its nonheritable determinants (Fig 4F and 4G), community selection failed to improve community function (Fig 3B).

### Reducing nonheritable variations in an experimentally feasible manner promotes artificial community selection

Reducing nonheritable variations in community function should facilitate community selection. One possibility would be to reduce stochastic fluctuations in nonheritable determinants. For example, a cell sorter could allocate to each Newborn community a fixed biomass or cell number from each species, if different species have different fluorescence [61]. Indeed, in simulations, when we fixed Newborn’s total biomass and species fraction ("cell sorting"; Methods, Section 6), community function became strongly correlated with its heritable determinant (Fig 3F). In this case, average cost ${\bar{f}}_{P}$ and community function *P*(*T*) both increased under selection (Fig 3D and 3E) to near the optimal. Improvement was not seen if either Newborn total biomass or species fraction was allowed to fluctuate stochastically (S12A Fig). Community function also improved if fixed numbers of H and M cells (instead of biomass) were allocated into each Newborn, even though each cell’s biomass fluctuated between 1 and 2 (S13C Fig; Methods, Section 6).

Nonheritable variations in community function could also be curtailed by reducing the dependence of community function on nonheritable determinants. For example, we could extend the maturation time *T* to nearly deplete Resource. In this selection regimen, Newborns would still experience stochastic fluctuations in total biomass and species composition. However, "unlucky" communities would have time to "catch up" as "lucky" communities wait in stationary phase. Indeed, with this extended maturation time *T*, community function became strongly correlated with its heritable determinant ${\bar{f}}_{P}(0),$ and community function improved without having to fix Newborn total biomass or species composition (Fig 3J–3L). However, at long maturation time, nonheritable variations in community function could still arise from stochastic fluctuations in the duration of stationary phase (which could affect cell survival or recovery time in the next selection cycle). Thus, for most simulations, we used short maturation time unless stated otherwise.

### Reducing inter-community selection strength can promote selection when nonheritable variations hinder selection

Since the highest community function may not correspond to the highest *f*_*P*_ (Fig 3C), we examined whether a "top-tier" strategy might outperform the top-dog strategy. In the top-tier strategy, we chose, for example, the top 10% Adults, allowing each to reproduce 10 Newborns. When we partitioned Adults into Newborns as if using pipetting, although community function failed to improve under the top-dog strategy, it improved somewhat under the top-tier strategy (Fig 3, compare G-I with A-C). In fact, the top-tier strategy improved community function under a wide range of selection strengths (e.g., top 5% to top 50%; S15 Fig). When we chose top 2% (2 Adults, each contributing 50 Newborns), community function did not improve (S15 Fig). When we chose all 100 Adults (each contributing 1 Newborn), community function declined to zero as expected (S15 Fig), because there was no intercommunity selection.

The superiority of top-tier over top-dog strategy rests on giving "unlucky" Adults a chance to reproduce. We reached this conclusion by noting that if we minimized nonheritable variations in community function (by fixing total biomass and species fraction in Newborns), then top-dog is superior to top-tier strategy (S16 Fig). Also note that the top-tier strategy in the presence of nonheritable variations (pipetting; Fig 3G–3I and S14 Fig) does not work as effectively as eliminating nonheritable variations (cell sorting; Fig 3D–3F).

**Fig 5:**
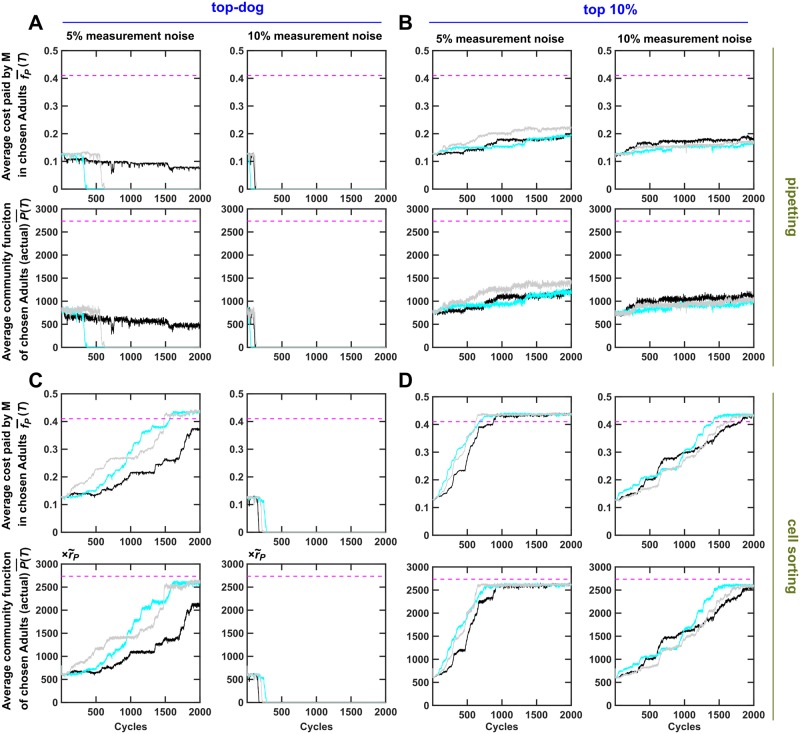
Ineffective selection due to community function measurement noise can be rescued by the top-tier strategy acting in synergy with cell sorting. Adult communities were chosen to reproduce based on "measured community function *P*(*T*)"—the sum of actual *P*(*T*) and a "noise term" randomly drawn from a normal distribution with zero mean and standard deviations of 5% or 10% of the ancestral *P*(*T*). Dynamics of average *f*_*P*_ and average community function of the chosen Adult communities (${\bar{f}}_{P}(T)$ and $\bar{P}(T)$) are plotted. When community function measurement noise is low (5%), cell sorting largely rescues ineffective community selection (A–D, left panels). When community function measurement noise is high (10%), both cell sorting and top-tier strategy are required (A–D, right panels). Black, cyan, and gray curves represent independent simulation trials. $\bar{P}(T)$ was averaged across the chosen Adults. ${\bar{f}}_{P}(T)$ was obtained by first averaging among M within each chosen Adult and then averaging across the chosen Adults. The simulation codes can be found in S3 Code, and the data can be found in S2 Data. Adult, Adult community; M, Manufacturer.

As expected, community function measurement noise—another source of nonheritable variation—interferes with community selection (compare Fig 3A and 3B with Fig 5A; Fig 3D and 3E with Fig 5C). In this case, community selection can be improved by leveraging both the top-tier strategy and cell sorting (Fig 5, compare B and C with D). This is presumably because (i) cell sorting reduces nonheritable variations in community function and (ii) the top-tier strategy gives “unlucky” communities suffering unfavorable community function measurement errors a chance to reproduce.

### Effective community selection can enforce species coexistence

Properly executed community selection could even improve the functions of communities whose member species may not coexist. Consider an H-M community in which, unlike the H-M community we have considered so far, H had the evolutionary potential to grow much faster than M. In this case, high community function not only required M to pay a fitness cost of *f*_*P*_ but also required H to pay a fitness cost by growing sufficiently slowly to not outcompete M.

**Fig 6:**
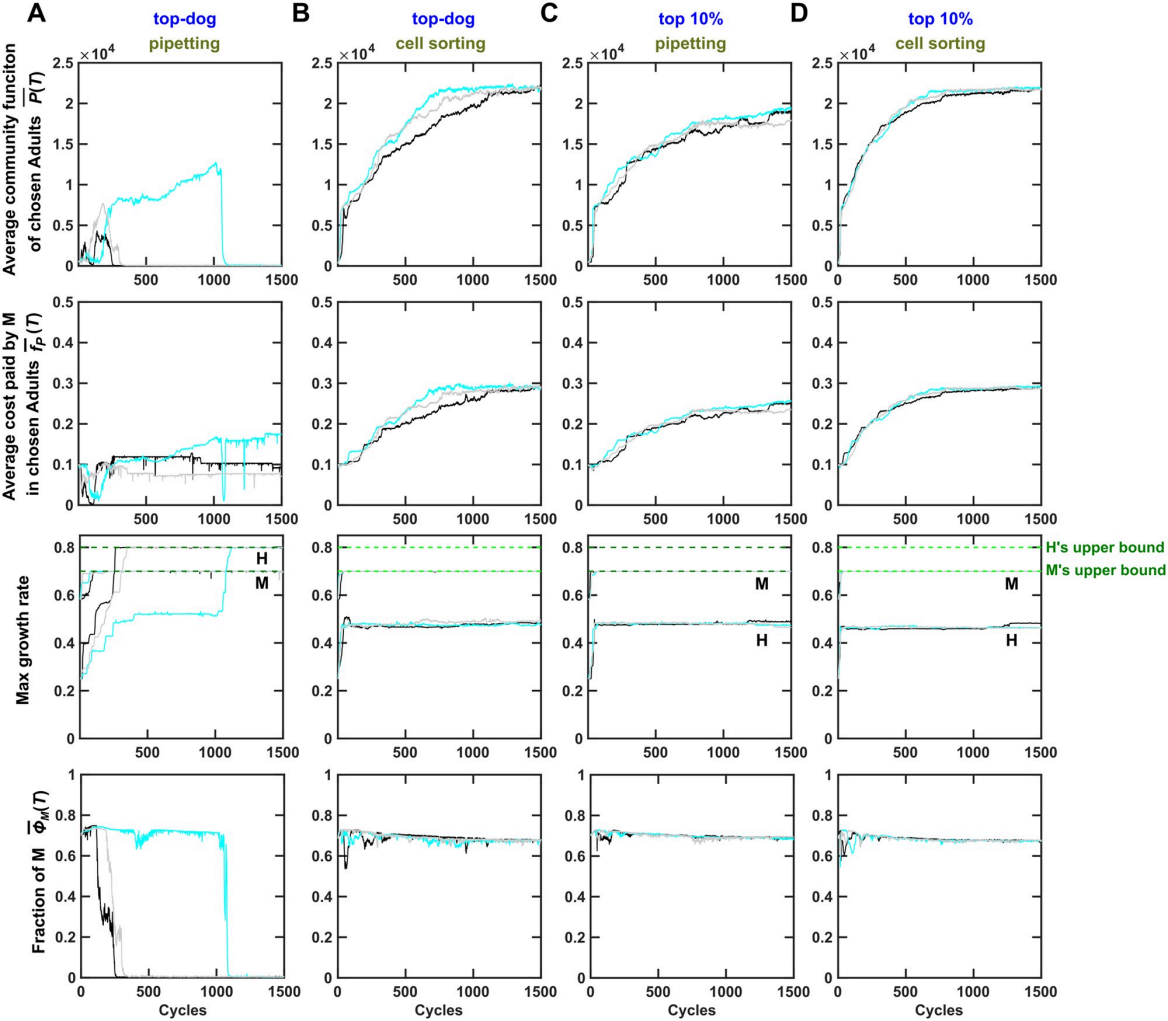
Effective community selection can encourage species coexistence. Here, the evolutionary upper bound for *g*_*Hmax*_ ($g_{Hmax}^{*}=0.8$) was larger than that for *g*_*Mmax*_ ($g_{Mmax}^{*}=0.7$), opposite to that in Figs 2–5. (A) When the top-dog strategy and pipetting were used to choose and reproduce Adult communities, M was almost outcompeted by H as H evolved to grow faster than M (rows 3 and 4). Although M would ordinarily go extinct, community selection managed to maintain M at a very low level (bottom). This imbalanced species ratio resulted in very low community function (top). (B-D) When community selection was effective, using top-dog with cell sorting (panel B), top-tier with pipetting (panel C), or top-tier with cell sorting (panel D), community selection successfully improved community function and ${\bar{f}}_{P}$. In these cases, H’s growth parameter did not increase to its evolutionary upper bound (panel B-D row 3, also see S17B–S17D Fig), allowing a balanced species ratio (panel B-D bottom) and high community function (panel B-D top). Resource supplied to Newborn communities here supports 10^5^ total biomass to accommodate faster growth rates (and hence community function is larger than in other figures). Black, cyan, and gray curves represent independent simulation trials. $\bar{P}(T)$ (average community function) and ${\bar{\phi }}_{M}(T)$ (average fraction of M biomass in Adult communities) were averaged across the chosen Adults. ${\bar{f}}_{P}(T)$ was obtained by first averaging among M within each chosen Adult and then averaging across all chosen Adults. The simulation codes can be found in S4 Code, and the data can be found in S3 Data. Adult, Adult community; H, Helper; M, Manufacturer.

We started community selection at ancestral growth parameters and allowed them and *f*_*P*_ to mutate. When community selection was ineffective (top-dog with pipetting; Fig 6A), H’s maximal growth rate evolved to its upper bound and exceeded M’s maximal growth rate (Fig 6A, row 3, S17A Fig). This drove M to almost extinction (Fig 6A, row 4), and community function was very low (Fig 6A, row 1). During effective community selection (top-dog with cell sorting, top 10% with pipetting, or top 10% with cell sorting), H’s maximal growth rate remained far below its evolutionary upper bound and below M’s maximal growth rate (Fig 6B–6D, row 3). This is because if H’s maximal growth rate in a community had evolved to be too high, then H would drive M to low abundance, and the resulting low community function would be disfavored by intercommunity selection. Because H was constrained to grow slower than M, H and M can coexist at a moderate ratio, and community function improved (Fig 6B–6D, row 1). Note that, unlike Fig 5, here top-tier and cell sorting did not show synergism. This is because when nonheritable variation in community function is minimized by cell sorting, the top-tier strategy does not seem to be helpful (S16 Fig).

### Robust conclusions under alternative model assumptions

We have demonstrated that when selecting for high H-M community function, seemingly innocuous experimental procedures (e.g., choosing the top-functioning Adults and pipetting portions of them to form Newborns) could be problematic. Instead, more precise procedures (e.g., cell sorting) or moderate intercommunity selection strength (e.g., the top-tier strategy) might be required. Our conclusions held when we used a much lower mutation rate (2 × 10^−5^ instead of 2 × 10^−3^ mutation per cell per generation per phenotype, S18 Fig), although lower mutation rate slowed down community function improvement. Our conclusions also held when we used different distributions of mutation effects (S19 Fig) or incorporated epistasis (i.e., a non-null mutation would likely reduce *f*_*P*_ if the current *f*_*P*_ was high and increase *f*_*P*_ if the current *f*_*P*_ was low; S20 and S21 Figs; Methods Section 5).

To further test the generality of our conclusions, we simulated community selection on a mutualistic H-M community. Specifically, we assumed that Byproduct was inhibitory to H. Thus, H benefited M by providing Byproduct, and M benefited H by removing the inhibitory Byproduct, similar to the syntrophic community of *Desulfovibrio vulgaris* and *Methanococcus maripaludis* [62]. We obtained similar conclusions in this mutualistic H-M community (S22 Fig). We have also shown that similar conclusions hold for communities in which member species may not coexist (Fig 6).

In summary, our conclusions seem general under a variety of model assumptions and apply to a variety of communities.

## Discussion

A desired community function can be attained by identifying appropriate combinations of species types. For example, by using cellulose as the main carbon source in a process called "enrichment," Kato and colleagues obtained a community consisting of a few species that together degrade cellulose [63]. However, if we solely rely on species types, then without a constant influx of new species, community function will likely level off quickly [14,17,118]. Here, we consider artificial selection of communities with defined member species so that improvement of community function requires new genotypes that contribute more toward community function at a higher cost to itself.

### Community selection can be challenging but is feasible

Artificial selection of whole communities to improve a costly community function requires careful considerations. These considerations include species choice (Figs 2 and 6), mutation rate (compare Fig 3 and S18 Fig), the total number of communities under selection, Newborn target total biomass (S7 Fig), the number of generations during maturation (which in turn depends on the amount of Resource added to each Newborn and the maturation time; S7 Fig), intercommunity selection strength (Fig 3, S15 and S16 Figs), how we reproduce Adults (e.g., pipetting versus cell sorting; Fig 3), and the uncertainty in community function measurements (Fig 5).

Many of these considerations face dilemmas. For example, a large Newborn size (*BM*_*target*_) might lead to reproducible takeover by noncontributors (S7 Fig), but a small Newborn size would mean that large nonheritable variations in community function can readily arise and interfere with selection unless special measures are taken (Fig 3).

We can take obvious steps to mitigate nonheritable variations in community function. For example, we can repeatedly measure community function to increase measurement precision, thereby facilitating selection (Fig 5). We can also use the top-tier strategy so that unlucky communities harboring desirable genotypes can have a chance to reproduce (Figs 3 and 5). Note that a top-tier strategy had often been implemented during artificial individual selection [114] and artificial community selection [119] to maintain variations among entities. Although some community functions (such as steady-state species ratio or steady-state growth rate of mutualistic communities) are not sensitive to fluctuations in Newborn biomass compositions [40,64]; for those that are, we can use a cell sorter to fix Newborn species biomass compositions to reduce nonheritable variations in community function (Figs 3 and 5).

The need to suppress nonheritable variations in community function can have practical implications that may initially seem nonintuitive. For example, when shared Resource is nonlimiting (to avoid stationary phase), we must dilute a chosen Adult community to a fixed target biomass instead of by a fixed fold. This is because otherwise, selection would fail as we choose larger and larger Newborn size instead of higher and higher *f*_*P*_ (Methods, Section 7; S23 Fig).

The definition of community function is also critical. If we had defined community function as Product per M biomass in the Adult community *P*(*T*)/*M*(*T*) (which is approximately proportional to $\frac{f_{P}}{1-f_{P}}$: see Methods Section 7), then we would be selecting for higher and higher *f*_*P*_, and M can go extinct (S2 Fig).

### Intracommunity selection versus intercommunity selection

Intracommunity selection and intercommunity selection are both important. Intracommunity selection occurs during community maturation and favors fast growers. Intercommunity selection occurs prior to community reproduction and favors high community function.

For M’s cost *f*_*P*_, intracommunity selection favors low *f*_*P*_, while intercommunity selection favors $f_{P}^{*}$, the *f*_*P*_ value leading to the highest community function. Thus, when current $f_{P}<f_{P}^{*}$, intercommunity selection runs against intracommunity selection. When current $f_{P}>f_{P}^{*}$, intracommunity and intercommunity selections are aligned.

For growth parameters (maximal growth rates, affinities for metabolites), depending on their evolutionary upper bounds, intercommunity selection may or may not be aligned with intracommunity selection. For example, using parameters in Table 1 (Fig 3), improving growth parameters promoted community function (S8A–S8C Fig; S24A Fig). This is because with these choices of evolutionary upper bounds, H could not evolve to grow so fast to overwhelm M. Thus, with sufficient Resource and with species coexistence (Fig 2A top), faster H and M growth resulted in more Byproduct, larger M populations, and consequently higher Product level. If H could evolve to grow faster than M, then increasing growth parameters could decrease community function due to H dominance (Fig 6A; S17A Fig; S24B Fig). Note that even in this case, properly executed community selection can promote species coexistence and improve community function (Fig 6B–6D).

### Contrasting selection at different levels

Selection of individuals bears some resemblance to selection of communities. Whereas community function relies on interactions between different species, an individual’s fitness relies on interactions between different genes. To ensure sufficient heredity between an individual and its offspring, elaborate cellular mechanisms have evolved. They include cell cycle checkpoints to ensure accurate DNA replication and segregation [65], small RNA-mediated silencing of transposons [66], and clustered regularly interspaced short palindromic repeats and the CRISPR-associated protein (CRISPR-Cas) degradation of foreign viral DNA [67]. In community selection, heredity-enhancing mechanisms such as stable species ratio (Fig 2A top panel) could already be in place due to ecological interactions or arise due to evolution (e.g., mutations that affect ecological interactions). If endosymbiosis should evolve in response to community selection (i.e., one microbe stably living inside another microbe much like chloroplasts living inside plant cells), then community selection would transition to individual selection.

Group selection is connected to individual selection and community selection. Group selection, and in a related sense kin selection [42–55,68], have been extensively examined to explain, e.g., the evolution of traits that lower individual fitness but increase the success of a group (e.g., sterile ants helping the survival of an ant colony). Note that the term "group selection" has often been used to describe individual selection in spatially structured populations without group births or deaths, although such usage has been criticized [69]. Artificial group selection can sometimes be viewed as artificial individual selection. For example, when Newborn groups start with a single founder producing a product of interest, then artificial group selection for high group production is equivalent to artificial individual selection for the founder’s ability to produce over time as it grows into a population. On the other hand, if group function relies on synergistic interactions between populations with distinct phenotypes, then group selection can be thought of as a special case of community selection. The difference is that, unlike community function, group function can arise as a single founder multiplies and differentiates into distinct populations (e.g., filamentous cyanobacteria growing and differentiating into nitrogen-fixing cells and photosynthetic cells [70]).

Group selection and community selection display additional similarities and distinctions. First, group selection and community selection are similar in that Newborn size must not be too large [71,72], and maturation time must not be too long. Otherwise, all entities (groups or communities) will accumulate noncontributors in a similar fashion, and this lack of variation among entities impedes selection (Price equation [56]; S1B Fig; S7 Fig). Second, species interactions in a community could drive species composition to a value suboptimal for community function [73]. A similar constraint could also occur during artificial group selection if the founder genotype gives rise to interacting subpopulations. Otherwise, the problem of suboptimal composition does not exist for group selection. Finally, in group selection, when a Newborn group starts with a small number of individuals (e.g., one individual), a fraction of Newborn groups of the next cycle will be highly similar to the original Newborn group (S1B Fig, bottom panel). This heredity facilitates group selection. In contrast, when a Newborn community starts with a small number of total individuals, large stochastic fluctuations in Newborn composition can interfere with community selection (Figs 3 and 4). In the extreme case, a member species may even be lost by chance. Even if a fixed biomass of each species is allocated into Newborns during community selection, heredity is much reduced due to random sampling of genotypes from multiple species. For example, if Newborn communities start with one contributor from each of the 2 species and if the highest-functioning Adult community has accumulated 50% noncontributors in each species, then only 50%×50% = 25% Newborn communities of the next cycle will be similar to the original Newborns. In contrast, if Newborn groups are initiated with a single contributor and if the highest-functioning Adult group has accumulated 50% noncontributors, then 50% Newborn groups of the next cycle will be similar to the original Newborn.

### Community function may not be maximized through pre-optimizing member species in monocultures

If we know how each member species contributes to community function, might we pre-optimize member species in monocultures before assembling them into high-functioning communities? This turns out to be challenging due to the difficulty of recapitulating community dynamics in monocultures. For example, artificial group selection on M failed to increase M’s cost *f*_*P*_ to $f_{P}^{*}$ optimal for community function. Specifically, we started simulations with *n*_*tot*_ of 100 Newborn M groups, each inoculated with one M cell (to facilitate group selection, S1B Fig bottom panel) [71]. We supplied each Newborn M group with the same amount of Resource as we would for H-M communities as well as excess Byproduct (since it is difficult to reproduce community Byproduct dynamics in M groups). After incubating these M groups for the same maturation time *T*, the group with the highest level of Product would be chosen and reproduced into Newborn M groups (initiated with a single cell) for the next cycle. M’s growth parameters improved to evolutionary upper bounds (S25A Fig), because faster-growing M cells would lead to higher group function in the presence of sufficient Resource and excess Byproduct. When growth parameters were fixed to evolutionary upper bounds, optimal *f*_*P*_ for monoculture *P*(*T*) could be calculated to occur at an intermediate value ($f_{P,Mono}^{*}=0.13$; S25B Fig). Optimal group function was indeed realized during selection (S25A Fig). However, the associated optimal *f*_*P*_ was much lower than that for community function ($f_{P}^{*}=0.41$; see Methods Section 8 for an explanation). Thus, optimizing monoculture activity does not necessarily lead to optimized community function.

### Implications of our work

Our work sheds new light on previous work. In the work of Swenson and colleagues [12], authors tested 2 selection regimens with Newborn sizes differing by 100-fold. The authors hypothesized that smaller Newborns would have a high level of variation that should facilitate selection. However, the hypothesis was not corroborated by experiments. As a possible explanation, the authors invoked the "butterfly effect" (the sensitivity of chaotic systems to initial conditions). Our results suggest that even for nonchaotic systems like the H-M community, selection could fail due to interference from nonheritable variations. This is because in Newborns with small sizes, fluctuations in community composition can be large, which compromises heredity of a community trait.

A general implication of our work is that before launching a selection experiment, one should carefully design the selection regimen. For example, one may want to check the sensitivity of community function to fluctuations in Newborn biomass composition. The first method of checking involves estimating the "signal to noise" ratio: one could use the most precise method to initiate Newborn community replicates and measure community functions (e.g., cell sorting during Newborn formation; many repeated measurements of community function). Despite this, some levels of nonheritable variations in community function are inevitable due to, e.g., nongenetic phenotypic variations among cells [74] or stochasticity in cell birth and death. If "noises" (variations among replicate communities) are small compared to "signals" (variations among communities of different genotypes that affect the community function of interest), then one can test and possibly adopt less precise procedures (e.g., cell culture pipetting during Newborn formation; fewer repeated measurements of community function). The second checking method involves empirically estimating the heritability of community function, especially if significant variations in community function naturally arise within the first few cycles (due to, e.g., preexisting mutations). In this case, one could experimentally evaluate whether community functions of the previous cycle are correlated with community functions of the current cycle across independent lineages (similar to Fig 4). Given the ubiquitous nature of nonheritable variations in community function, the top-tier strategy could be useful (Figs 5 and 6).

Our work also contributes to how we might think about community selection in the natural environment. Microbes can co-evolve with each other and with their host [75, 76, 77]. Some have proposed that complex microbial communities such as the gut microbiota could serve as a unit of selection [19]. Our work suggests that if selection for a costly microbial community function should occur in nature, then certain mechanisms may need to be in place. These mechanisms include (i) suppressing nonheritable variations in community function, and (ii) exerting an appropriate strength of intercommunity selection.

### Future directions

Our work touched upon only the tip of the iceberg of community selection. We expect that certain rules will be insensitive to details of a community. For example, community selection can be facilitated by reducing nonheritable variations in community function, or by mitigating the effects of nonheritable variations via top-tier strategies. Still, much more awaits further investigation. Here, we outline a few:

1. Explore the best strategies for choosing and reproducing Adult communities. We have chosen top 10% communities to contribute an equal number of Newborns, but alternative strategies (e.g., allowing higher-functioning Adults to contribute more Newborns) may work better.
2. Examine the impact of migration (community mixing) on community selection. Here, we did not allow migration. Excessive migration could deter community selection by allowing fast-growing noncontributors to spread. However, by combining the best genotypes of multiple member species, migration could speed up community selection, much like the effects of sexual recombination on the evolution of finite populations [78].
3. Investigate how interaction structure might affect selection efficacy. We have shown that our conclusions hold for 2-species communities engaging in commensalism or mutualism. We have also shown that our conclusions hold regardless of whether the 2 species can evolve to coexist or not. The next step would be to test other types of interactions and complex interaction networks. For example, when species mutually inhibit each other, multistability could arise. In this case, species dominance [79] and thus community function could be highly sensitive to stochastic fluctuations in Newborn species composition. How might multistability affect community selection [117,118]?
  A complex community might also have multiple local optima of community function. This can arise when, e.g., the maximal community function requires multiple species with partially redundant activities. Consider the community function of waste degradation. Suppose that one species is good at degrading waste at high concentration while the other is good at degrading waste at low concentration. Then, maximal waste degradation requires both species. If any one species is lost by chance during Newborn formation, then community function would be stuck at local suboptima. In this case, community migration (mixing) could recover the lost species and help community selection reach a global optimum.
4. Develop a general theory to understand the rate of community function improvement. Community function improvement depends on many experimental parameters, as we have demonstrated here. Ultimately, the rate of improvement will depend on variation and heredity of community function, which are affected by intracommunity and intercommunity selection. How might experimental parameters affect variation and heredity of community function under selection? This aspect might be explored through expanding population genetics theories which have so far focused on individual selection [114].
5. Experimentally test model predictions. Effective community selection will require a fast and precise assay for community function. If community function is sensitive to species biomass composition in Newborns, then species should ideally be distinguishable by flow cytometry (e.g., different fluorescence or different scattering patterns) so that fixed biomass of each species can be sorted into Newborns. Note that cell sorting only needs to be performed on several high-functioning communities and thus would not be cost prohibitive if the total number of Newborn communities is moderate.
6. Discover drivers of community function. Once high-functioning communities are obtained through selection, one could compare metagenomes of evolved communities with those of ancestral communities. This could illuminate genes and species interactions that are important for community function.

## Methods

### 1. Equations of H-M community

*H*, the biomass of H, changes as a function of growth and death,

$$\frac{dH}{dt}=g_{H}(\hat{R})H-\delta_{H}H$$ (1)

Growth rate g_*H*_ depends on the level of Resource $\hat{R}$ (hat ^ representing prescaled absolute value) as described by the Monod growth model

$$g_{H}(\hat{R})=g_{Hmax}\frac{\hat{R}}{\hat{R}+{\hat{K}}_{HR}}$$

where ${\hat{K}}_{HR}$ is the $\hat{R}$ at which *g*_*Hmax*_/2 is achieved. *δ*_*H*_ is the death rate of H. Note that because agricultural waste is in excess, its level does not enter the equation.

*M*, the biomass of M, changes as a function of growth and death,

$$\frac{dM}{dt}=(1-f_{P})g_{M}(\hat{R},\hat{B})M-\delta_{M}M$$ (2)

Total potential growth rate of M *g*_*M*_ depends on the levels of Resource and Byproduct ($\hat{R}$ and $\hat{B}$) according to the Mankad-Bungay model [30] which has received experimental support (S4 Fig)

$$g_{M}(\hat{R},\hat{B})=g_{Mmax}\frac{{\hat{R}}_{M}{\hat{B}}_{M}}{{\hat{R}}_{M}+{\hat{B}}_{M}}\left(\frac{1}{{\hat{R}}_{M}+1}+\frac{1}{{\hat{B}}_{M}+1}\right)$$

where ${\hat{R}}_{M}=\hat{R}/{\hat{K}}_{MR}$ and ${\hat{B}}_{M}=\hat{B}/{\hat{K}}_{MB}$ (S3 Fig). Out of total potential growth rate of M, 1 − *f*_*P*_ fraction is channeled to biomass increase, while *f*_*P*_ fraction is channeled to making Product

$$\frac{d\hat{P}}{dt}={\tilde{r}}_{P}f_{P}g_{M}(\hat{R},\hat{B})M$$ (3)

where ${\tilde{r}}_{P}$ is the amount of Product made at the cost of one M biomass (tilde “~” represents scaling factor; see below and Table 1).

Resource $\hat{R}$ is consumed proportionally to the growth of M and H; Byproduct $\hat{B}$ is released proportionally to H growth and consumed proportionally to M growth

$$\frac{d\hat{R}}{dt}=-{\hat{c}}_{RM}g_{M}(\hat{R},\hat{B})M-{\hat{c}}_{RH}g_{H}(\hat{R})H$$ (4)

$$\frac{d\hat{B}}{dt}={\tilde{r}}_{B}g_{H}(\hat{R})H-{\hat{c}}_{BM}g_{M}(\hat{R},\hat{B})M$$ (5)

Here, ${\hat{c}}_{RM}$ and ${\hat{c}}_{RH}$ are the amounts of $\hat{R}$ consumed per potential M biomass and H biomass, respectively. ${\hat{c}}_{BM}$ is the amount of $\hat{B}$ consumed per potential M biomass. ${\tilde{r}}_{B}$ is the amount of $\hat{B}$ released per H biomass grown. Our model assumes that Byproduct or Product is generated proportionally to H or M biomass grown, which is reasonable given the stoichiometry of metabolic reactions and experimental support [80]. The volume of community is set to 1, and thus cell or metabolite quantities (which are considered here) are numerically identical to cell or metabolite concentrations.

In the equations above, scaling factors are marked by "~" and will become 1 after scaling. Variables and parameters with hats will be scaled and lose their hats afterwards. Variables and parameters without hats will not be scaled. We scale Resource-related variable ($\hat{R}$) and parameters (${\hat{K}}_{MR},{\hat{K}}_{HR},{\hat{c}}_{RM}$, and ${\hat{c}}_{RH}$) against $\overset{\sim }{R}(0)$ (Resource supplied to Newborn), Byproduct-related variable ($\hat{B}$) and parameters (${\hat{K}}_{MB}$ and ${\hat{c}}_{BM}$) against ${\tilde{r}}_{B}$ (amount of Byproduct released per H biomass grown), and Product-related variable ($\hat{P}$) against ${\tilde{r}}_{P}$ (amount of Product made at the cost of one M biomass). For biologists who usually think of quantities with units, the purpose of scaling (and getting rid of units) is to reduce the number of parameters. For example, H biomass growth rate can be rewritten as

$$\begin{array}{ll} g_{H}\left(\hat{R}\right) & =g_{Hmax}\frac{\hat{R}}{\hat{R}+{\hat{K}}_{HR}}\\ & =g_{Hmax}\left(\frac{\hat{R}}{\tilde{R}\left(0\right)}\right)/\left(\frac{\hat{R}}{\tilde{R}\left(0\right)}+\frac{{\hat{K}}_{HR}}{\tilde{R}\left(0\right)}\right)\\ & =g_{Hmax}\frac{R}{\left(R+K_{HR}\right)}\\ & =g_{H}\left(R\right) \end{array}$$

where $R=\hat{R}/\overset{\sim }{R}(0)$ and $K_{HR}={\hat{K}}_{HR}/\overset{\sim }{R}(0)$. Thus, the unscaled $g_{H}(\hat{R})$ and the scaled *g*_*H*_(*R*) share identical forms (S3 Fig). After scaling, the value of $\overset{\sim }{R}(0)$ becomes irrelevant (1 with no unit). Similarly, because ${\hat{R}}_{M}=\frac{\hat{R}}{\overset{\sim }{R}(0)}/\frac{{\hat{K}}_{MR}}{\overset{\sim }{R}(0)}=\frac{R}{K_{MR}}=R_{M}$ and ${\hat{B}}_{M}=\frac{\hat{B}}{{\tilde{r}}_{B}}/\frac{{\hat{K}}_{MB}}{{\tilde{r}}_{B}}=\frac{B}{K_{MB}}=B_{M},g_{M}(\hat{R},\hat{B})=g_{M}(R,B)$ (S3 Fig).

Thus, scaled equations are as follows:

$$\frac{dH}{dt}=g_{H}(R)H-\delta_{H}H$$ (6)

$$\frac{dM}{dt}=(1-f_{P})g_{M}(R,B)M-\delta_{M}M$$ (7)

$$\begin{array}{ll} \frac{dP}{dt} & =\frac{d\hat{P}}{{\tilde{r}}_{P}dt}\\ & =f_{P}g_{M}\left(\hat{R},\hat{B}\right)M\\ & =f_{P}g_{M}\left(R,B\right)M \end{array}$$ (8)

$$\begin{array}{ll} \frac{dR}{dt} & =\frac{d\hat{R}/\tilde{R}\left(0\right)}{dt}\\ & =-\frac{{\hat{c}}_{RM}}{\tilde{R}\left(0\right)}g_{M}\left(\hat{R},\hat{B}\right)M-\frac{{\hat{c}}_{RH}}{\tilde{R}\left(0\right)}g_{H}\left(\hat{R}\right)H\\ & =-c_{RM}g_{M}\left(R,B\right)M-c_{RH}g_{H}\left(R\right)H \end{array}$$ (9)

$$\begin{array}{ll} \frac{dB}{dt} & =\frac{d\hat{B}/{\tilde{r}}_{B}}{dt}\\ & =g_{H}\left(\hat{R}\right)H-\frac{{\hat{c}}_{BM}}{{\tilde{r}}_{B}}g_{M}\left(\hat{R},\hat{B}\right)M\\ & =g_{H}\left(R\right)H-c_{BM}g_{M}\left(R,B\right)M \end{array}$$ (10)

We have not scaled time here, although time can also be scaled by, e.g., the community maturation time. Here, time has the unit of unit time (e.g., hour), and to avoid repetition, we often drop the time unit. Scaling factors and values of species phenotypes after scaling are in Table 1. Additional symbols, including state variables and selection scheme parameters, are summarized in Table 2.

**Table 1:** Parameters for ancestral and preadapted H and M.

Symbols	Definition	Ancestral	Preadapted
${\tilde{r}}_{B}$	Amount of $\hat{B}$ released per H biomass grown	Scaling factor, 1	No change
${\tilde{r}}_{P}$	Amount of $\hat{P}$ released at the cost of one M biomass	Scaling factor, 1	No change
$\overset{\sim }{R}(0)$	Initial amount of Resource in Newborn	Scaling factor, 1	
*f*_*P*_	Fraction of M growth diverted to producing Product (M’s cost)	0.10	0.13^#^
*K*_*MR*_	Fold of $\overset{\sim }{R}(0)$ at which *g*_*Mmax*_/2 is achieved in excess B	1	1/3^†^
*K*_*MB*_	Amount of $\hat{B}$ at which *g*_*Mmax*_/2 is achieved in excess R, scaled against ${\tilde{r}}_{B}$	$\frac{5}{3}\times 10^{2}$	$\frac{1}{3}\times 10^{2}$^†^
*K*_*HR*_	Fold of $\overset{\sim }{R}(0)$ at which *g*_*Hmax*_/2 is achieved	1	1/5^†^
*g*_*Mmax*_	Maximal biomass growth rate of M	0.58/unit time	0.7/unit time*
*g*_*Hmax*_	Maximal biomass growth rate of H	0.25/unit time	0.3/unit time*
*δ*_*M*_	Death rate of M	3.5 × 10^−3^/unit time	No change
*δ*_*H*_	Death rate of H	1.5 × 10^−3^/unit time	No change
*c*_*RM*_	Fraction of $\overset{\sim }{R}(0)$ consumed per M biomass grown	10^−4^	No change
*c*_*RH*_	Fraction of $\overset{\sim }{R}(0)$ consumed per H biomass grown	10^−4^	No change
*c*_*BM*_	Amount of $\hat{B}$ consumed per M biomass grown, scaled against ${\tilde{r}}_{B}$	$\frac{1}{3}$	No change
*P*_*mut*_	Mutation probability per cell division for each mutable phenotype	2 × 10^−5^~2 × 10^−3^	

Tilde “~” means scaling factor, and hat “^” means prescaled absolute quantity (Methods, Section 1).

*Evolutionary upper bound for maximal growth rates for most simulations except Fig 6, S17 and S24B Figs where *g*_*Hmax*_ was allowed to increase to 0.8/unit time. In the latter case, the initial R(0) was increased to 10 units of $\overset{\sim }{R}(0)$.

^†^Evolutionary lower bound for *K*_*SpeciesMetabolite*_, which corresponds to evolutionary upper bound for Species’s affinity for Metabolite (1/*K*_*SpeciesMetabolite*_).

^#^*f*_*P*_ optimal for M-group function (S25B Fig).

Parameters in the "Preadapted" column are used as the phenotypes at the beginning of most simulations unless otherwise specified. By “preadapt”, we mean that all growth parameters are at their evolutionary upper bounds, and that *f*_*P*_ is at the ancestral state (0.1~0.13). Methods Section 2 explains our parameter choices (including why some parameters are held constant during evolution).

**Table 2:** Additional symbols used in the simulation.

Symbols	Definition
*T*	Community maturation time, corresponding to the duration of a selection cycle
*t*	Time within a selection cycle, 0 ≤ *t* ≤ *T*
*M*(*t*), *H*(*t*)	The biomass of M or H in a community at time *t*
*BM*(*t*) = *M*(*t*) + *H*(*t*)	The total biomass in a community at time *t*
*ϕ*_*M*_(*t*)	The fraction of M biomass at time *t*
*BM*_*target*_	Preset target total biomass of Newborns during community reproduction
*I*_*M*_(*t*), *I*_*H*_(*t*)	The integer number of M or H cells in a community at time *t*
*φ*_*M*_(*t*)	The fraction of M individuals at time *t*
*L*_*M*_(*t*), *L*_*H*_(*t*)	The biomass (length) of an individual M or H cell at time *t*, 1 ≤ *L*_*M*_(*t*), *L*_*H*_(*t*) ≤ 2
$\bar{L}$	The average biomass (length) of an individual M or H cell, set to 1.5
*P*(*t*)	The amount of Product P in a community at time *t*, scaled by ${\tilde{r}}_{P}$
*R*(*t*)	The amount of Resource R remaining in a community at time *t*, scaled by $\tilde{R}(0)$
*B*(*t*)	The amount of Byproduct B in a community at time *t*, scaled by ${\tilde{r}}_{B}$
*n*_*D*_	The fold dilution when reproducing an Adult community
*n*_*chosen*_	Number of Adult communities chosen to reproduce
*n*_*tot*_	Total number of communities under selection in each cycle

Below, we calcuate the steady-state species ratio in the H-M community. From Eq 10:

$$\int_{0}^{T}\frac{dB}{dt}dt=\int_{0}^{T}g_{H}(R)Hdt-\int_{0}^{T}c_{BM}g_{M}(R,B)Mdt.$$ (11)

If we approximate Eqs 6–7 by ignoring the death rates so that $\frac{dH}{dt}\approx g_{H}(R)H$ and $\frac{dM}{dt}\approx (1-f_{P})g_{M}(R,B)M$, Eq 11 becomes

$$B(T)\approx \int_{0}^{T}\frac{dH}{dt}dt-\frac{c_{BM}}{1-f_{P}}\int_{0}^{T}\frac{dM}{dt}dt.$$ (12)

In our simulations, with our parameter choices, if *f*_*P*_ is not too large (*f*_*P*_ < 0.4), *B*(*T*) ≈ 0. If *T* is large enough so that both M and H have multiplied significantly and *H*(*T*) ≫ *H*(0) and *M*(*T*) ≫ *M*(*0*), Eq 12 becomes

$$H(T)-H(0)-\frac{c_{BM}}{1-f_{P}}(M(T)-M(0))\approx H(T)-\frac{c_{BM}}{1-f_{P}}M(T)\approx 0,$$

and the M:H ratio at time *T* is

$$\frac{M(T)}{H(T)}\approx \frac{1-f_{P}}{c_{BM}}.$$ (13)

Thus when Byproduct *B*(*T*) ≈ 0, *ϕ*_*M*,*SS*_, the steady-state fraction of M biomass, is then

$$\phi_{M,SS}\approx \frac{1-f_{P}}{1-f_{P}+c_{BM}}.$$ (14)

For *f*_*P*_ < 0.4, Eq 14 is applicable and predicts the steady-state *ϕ*_*M*,*SS*_ well (see S26 Fig). Note that significant deviation occurs when *f*_*P*_ > 0.4. This is because when *f*_*P*_ is large, M’s biomass does not grow fast enough to deplete B so that we cannot approximate *B*(*T*) ≈ 0 anymore.

### 2. Parameter choices

Our parameter choices are based on experimental measurements from a variety of organisms. Additionally, we chose growth parameters (maximal growth rates and affinities for metabolites) of ancestral and evolved H and M so that (i) the 2 species can coexist at a moderate ratio for a range of *f*_*P*_ over selection cycles and (ii) improving all growth parameters up to their evolutionary upper bounds generally improves community function (Methods Section 3). This way, we could simplify our simulation by fixing growth parameters at their respective evolutionary upper bounds. With only 1 mutable parameter (*f*_*P*_), we can identify the optimal $f_{P}^{*}$ associated with maximal community function (Fig 2B).

For ancestral H, we set *g*_*Hmax*_ = 0.25 (equivalent to 2.8-hour doubling time if we choose hour as the time unit), *K*_*HR*_ = 1 and *c*_*RH*_ = 10^−4^ (both with unit of $\overset{\sim }{R}(0)$) (Table 1). This way, ancestral H can grow by about 10-fold by the end of *T* = 17. These parameters are biologically realistic. For example, for a *lys- S*. *cerevisiae* strain with lysine as Resource, unscaled Monod constant is $\hat{K}=2\;\mu \text{M}$, and consumption $\hat{c}$ is 5 fmole/cell (Ref. [83]’s Fig 2 and Ref. [64]’s S1 Data). Thus, if we choose 25 μL as the community volume $\hat{V}$ and 2 μM as the initial Resource concentration, then $\overset{\sim }{R}(0)=5\times 10^{4}$ fmole. After scaling, $K=\hat{K}\hat{V}/\overset{\sim }{R}(0)=1$ and $c=\hat{c}/\overset{\sim }{R}(0)=10^{-4}$, comparable to the values in Table 1.

To ensure the coexistence of H and M, M must grow faster than H for part of the maturation cycle since M has to wait for H’s Byproduct at the beginning of a cycle. Because we have assumed M and H to have similar affinities for R (Table 1), *g*_*Mmax*_ must exceed *g*_*Hmax*_, and M’s affinity for Byproduct (1/*K*_*MB*_) must be sufficiently large. Moreover, metabolite release and consumption need to be balanced to avoid extreme species ratios. Thus, for ancestral M, we chose *g*_*Mmax*_ = 0.58 (equivalent to a doubling time of 1.2 hours). We set $c_{BM}=\frac{1}{3}$ (units of ${\tilde{r}}_{B}$), meaning that Byproduct released during one H biomass growth is sufficient to generate 3 potential M biomass, which is biologically achievable [40, 81]. When we chose $K_{MB}=\frac{5}{3}\times 10^{2}$ (units of ${\tilde{r}}_{B}$), H and M can coexist for a range of *f*_*P*_ (Fig 2A). This value is biologically realistic. For example, suppose that H releases hypoxanthine as Byproduct. A hypoxanthine-requiring *S*. *cerevisiae* strain evolved under hypoxanthine limitation could achieve a Monod constant for hypoxanthine on the order of 0.1 μM [64]. If the volume of the community is 10 μL, then $K_{MB}=\frac{5}{3}\times 10^{2}$ (units of ${\tilde{r}}_{B}$) corresponds to an absolute release rate ${\tilde{r}}_{B}=0.1\;\mu \text{M}\times 10\;\mu \text{L}/(\frac{5}{3}\times 10^{2})=6$ fmole per releaser biomass born. At 8-hour doubling time, this translates to 6 fmole/(1 cell×8 h)≈ 0.75 fmole/cell/h, within the ballpark of experimental observation (approximately 0.3 fmole/cell/h [64]). As a comparison, a lysine-overproducing yeast strain reaches a release rate of 0.8 fmole/cell/h [64], and a leucine-overproducing strain reaches a release rate of 4.2 fmole/cell/h [81]. Death rates *δ*_*H*_ and *δ*_*M*_ were chosen to be 0.5% of H and M’s respective upper bound of maximal growth rate, which are within the ballpark of experimental observations (e.g., the death rate of a *lys-* strain in lysine-limited chemostat is 0.4% of maximal growth rate [64]).

We assume that H and M consume the same amount of Resource R per new cell (*c*_*RH*_ = *c*_*RM*_) because the biomass of various microbes share similar elemental (e.g., carbon or nitrogen) compositions [82]. Specifically, *c*_*RH*_ = *c*_*RM*_ = 10^−4^ (units of $\overset{\sim }{R}(0)$), meaning that the Resource supplied to each Newborn community can yield a maximum of 10^4^ total biomass.

In some simulations (e.g., Fig 6, S8 and S17 Figs), growth parameters (maximal growth rates *g*_*Mmax*_ and *g*_*Hmax*_ and affinities for nutrients 1/*K*_*MR*_, 1/*K*_*MB*_, and 1/*K*_*HR*_) and production cost parameter (0 ≤ *f*_*P*_ ≤ 1) were allowed to change from ancestral values during community maturation because these phenotypes have been observed to rapidly evolve within dozens to hundreds of generations [32, 33, 34, 35]. For example, several-fold improvement in nutrient affinity and 20% increase in maximal growth rate have been observed in experimental evolution [33,35]. We therefore allowed affinities 1/*K*_*MR*_, 1/*K*_*HR*_, and 1/*K*_*MB*_ to increase by up to 3-fold, 5-fold, and 5-fold, respectively, and allowed *g*_*Hmax*_ and *g*_*Mmax*_ to increase by up to 20%. These evolutionary upper bounds also ensured that evolved H and M could coexist for *f*_*P*_ < 0.5 and that Resource was, on average, not depleted by *T* to avoid stationary phase.

We also simulated community selection in which improved growth parameters could reduce community function (Fig 6, S17 and S24B Figs). In this simulation, *g*_*Hmax*_ was allowed to increase by up to 220%, and each Newborn community was supplied with Resource that can support up to 10^5^ cells (10 units of $\overset{\sim }{R}(0)$).

Although empirical studies sometimes find trade-off between maximal growth rate and nutrient affinity (e.g., [33]), for simplicity we assumed here that the two traits are independent of each other. We held metabolite consumption (*c*_*RM*_, *c*_*BM*_, *c*_*RH*_) constant because conversion of essential elements such as carbon and nitrogen into biomass is unlikely to evolve quickly and dramatically, especially when these elements are not in large excess [82]. Similarly, we held the scaling factors ${\tilde{r}}_{P}$ (Product released at the cost of one M biomass) and ${\tilde{r}}_{B}$ (Byproduct released per H biomass grown) constant, assuming that they do not change rapidly during evolution. Indeed, metabolite release rate and metabolite consumption amount per biomass (biomass measured as cell size) did not change significantly for a commonly-arising mutant in a yeast evolution experiment [83]. We held death rates (*δ*_*M*_, *δ*_*H*_) constant because they are much smaller than growth rates in general and thus any changes are likely inconsequential.

### 3. Choosing growth parameter ranges so that we can fix growth parameters to upper bounds

Improving growth parameters (maximal growth rate and affinity for metabolites) does not always lead to improved community function (Fig 6, S17 and S24B Figs). However, for most simulations, we have chosen H and M growth parameters so that improving them from their ancestral values up to evolutionary upper bounds generally improves community function (see below). H and M with growth parameters at upper bounds are called “preadapted”. When Newborn communities are assembled from preadapted H and M, 2 advantages are apparent.

First, after fixing growth parameters of H and M to their upper bounds, we can identify the locally maximal community function. Specifically, for a Newborn with total biomass *BM*(0) = 100 and fixed Resource *R*, we can calculate community function *P*(*T*) under various cost *f*_*P*_ and fraction M biomass in Newborn *ϕ*_*M*_(0), assuming that all M cells have the same *f*_*P*_. Since both numbers range between 0 and 1, we calculate *P*(*T*, *f*_*P*_ = 0.01 × *i*, *ϕ*_*M*_(0) = 0.01 × *j*) for integers *i* and *j* between 1 and 99. There is a single maximum for *P*(*T*) when *i* = 41 and *j* = 54. In other words, if M invests $f_{P}^{*}=0.41$ of its potential growth to make Product and if the fraction of M biomass in Newborn $\phi_{M}^{*}(0)=0.54$, then maximal community function P*(*T*) is achieved (Fig 2B; magenta dashed line in Fig 3).

Second, preadapted H and M are evolutionarily stable in the sense that deviations (reductions) from upper bounds will reduce both individual fitness and community function (S27 Fig), and are therefore disfavored by intracommunity selection and intercommunity selection.

Below, we present evidence that within our parameter ranges (Table 1), improving growth parameters generally improves community function. When *f*_*P*_ is optimal for community function ($f_{P}^{*}=0.41$), if we fix any 4 of the 5 growth parameters to their upper bounds, then as the remaining growth parameter improves, community function increases (magenta lines in top panels of S27 Fig). Moreover, mutants with a reduced growth parameter are outcompeted by their preadapted counterparts (magenta lines in bottom panels of S27 Fig).

When $f_{P}=f_{P,Mono}^{*}=0.13$ (optimal for M monoculture function in S25B Fig; the starting phenotype for most community selection simulations in this paper), community function and individual fitness generally increase as growth parameters improve (black dashed lines in S27 Fig). However, when M’s affinity for Resource (1/*K*_*MR*_) is reduced from upper bound, fitness improves slightly (black dashed line in S27J Fig). Mathematically speaking, this is a consequence of the Mankad-Bungay model [30] (S3B Fig). Let *R*_*M*_ = *R*/*K*_*MR*_ and *B*_*M*_ = *B*/*K*_*MB*_. Then,

$$\begin{array}{ll} \frac{\partial g_{M}}{\partial K_{MR}} & =\frac{\partial g_{Mmax}}{\partial R_{M}}\frac{\partial R_{M}}{\partial K_{MR}}=\frac{\partial \left[g_{Mmax}\frac{R_{M}B_{M}}{R_{M}+B_{M}}\left(\frac{1}{1+R_{M}}+\frac{1}{1+B_{M}}\right)\right]}{\partial R_{M}}\frac{\partial R_{M}}{\partial K_{MR}}\\ & =g_{Mmax}\frac{-R_{M}}{K_{MR}}\left[\frac{B_{M}\left(R_{M}+B_{M}\right)-R_{M}B_{M}}{{\left(R_{M}+B_{M}\right)}^{2}}\left(\frac{1}{1+R_{M}}+\frac{1}{1+B_{M}}\right)-\frac{R_{M}B_{M}}{R_{M}+B_{M}}\frac{1}{{\left(1+R_{M}\right)}^{2}}\right]\\ & =g_{Mmax}\frac{R_{M}B_{M}}{\left(R_{M}+B_{M}\right)K_{MR}}\left(\frac{R_{M}}{{\left(1+R_{M}\right)}^{2}}-\frac{B_{M}}{R_{M}+B_{M}}\left(\frac{1}{1+R_{M}}+\frac{1}{1+B_{M}}\right)\right) \end{array}$$

If *R*_*M*_ ≪ 1 ≪ *B*_*M*_ (corresponding to limiting R and abundant B),

$$\frac{R_{M}}{(1+R_{M}{)}^{2}}-\frac{B_{M}}{R_{M}+B_{M}}(\frac{1}{1+R_{M}}+\frac{1}{1+B_{M}})\approx \frac{R_{M}}{(1+R_{M}{)}^{2}}-\frac{1}{1+R_{M}}=-\frac{1}{(1+R_{M}{)}^{2}}$$ (15)

and thus $\frac{\partial g_{M}}{\partial K_{MR}}<0$. This is the familiar case in which growth rate increases as the Monod constant decreases (i.e., affinity increases). However, if *B*_*M*_ ≪ 1 ≪ *R*_*M*_

$$\frac{R_{M}}{(1+R_{M}{)}^{2}}-\frac{B_{M}}{R_{M}+B_{M}}(\frac{1}{1+R_{M}}+\frac{1}{1+B_{M}})\approx \frac{1}{R_{M}}-\frac{B_{M}}{R_{M}}(\frac{1}{1+B_{M}})=\frac{1}{R_{M}(1+B_{M})}$$ (16)

and thus $\frac{\partial g_{M}}{\partial K_{MR}}>0$. In this case, growth rate decreases as the Monod constant decreases (i.e., affinity increases). In other words, decreased affinity for the abundant nutrient improves growth rate. Transporter competition for membrane space [84] could lead to this result, since reduced affinity for abundant nutrient may increase affinity for rare nutrient.

At the beginning of each cycle, R is abundant and B is limiting (Eq 16). Therefore M cells with lower affinity for R will grow faster than those with higher affinity (S28A Fig). At the end of each cycle, the opposite is true (S28A Fig). As *f*_*P*_ decreases, M diverts more toward biomass growth, and the first stage of B limitation lasts longer. Consequently, M can gain a slightly higher overall fitness by lowering the affinity for R at low *f*_*P*_ (S28A Fig).

Regardless, decreased M affinity for Resource (1/*K*_*MR*_) only leads to a very slight increase in M fitness (S27J Fig, dashed line) and a very slight decrease in *P*(*T*) (S28B Fig). Moreover, this only occurs at low *f*_*P*_ at the beginning of community selection and thus may be neglected. Indeed, if we start all growth parameters at their upper bounds and *f*_*P*_ = 0.13, and perform community selection while allowing all parameters to vary (S29 Fig), then 1/*K*_*MR*_ decreases somewhat, yet the dynamics of *f*_*P*_ is similar to when we only allow *f*_*P*_ to change (compare S29D Fig with Fig 3A).

### 4. Mutation rate and the distribution of mutation effects

Literature values of mutation rate and the distribution of mutation effects are highly variable. Below, we briefly review the literature and discuss rationales of our choices.

Among mutations, a fraction is neutral in that they do not affect the phenotype of interest. For example, the vast majority of synonymous mutations are neutral [85]. Furthermore, mutations with small effects may appear neutral, which can depend on the effective population size and selection condition. For example, at low population size due to genetic drift (i.e., changes in allele frequencies due to chance), a beneficial or deleterious mutation may not be selected for or selected against and is thus neutral with respect to selection [86, 87]. As another example, the same mutation in an antibiotic-degrading gene can be neutral under low antibiotic concentrations but deleterious under high antibiotic concentrations [88]. We term all mutations with zero effects on phenotypes as "neutral" mutations.

Since a larger fraction of neutral mutations is equivalent to a lower rate of phenotype-altering mutations, our simulations define "mutation rate" as the rate of non-neutral mutations that either enhance a phenotype ("enhancing mutations") or diminish a phenotype ("diminishing mutations"). Enhancing mutations of maximal growth rates (*g*_*Hmax*_ and *g*_*Mmax*_) and of nutrient affinities (1/*K*_*HR*_, 1/*K*_*MR*_, 1/*K*_*MB*_) enhance the fitness of an individual ("beneficial mutations"). In contrast, enhancing mutations in *f*_*P*_ diminish the fitness of an individual ("deleterious mutations").

Depending on the phenotype, the rate of phenotype-altering mutations is highly variable. Mutations that cause qualitative phenotypic changes (e.g., drug resistance) occur at a rate of 10^−8^~10^−6^ per genome per generation in bacteria and yeast [89, 90]. In contrast, mutations affecting quantitative traits such as growth rate occur much more frequently. For example in yeast, mutations that increase growth rate by ≥ 2% occur at a rate of ∼ 10^−4^ per genome per generation (calculated from Fig 3 of Ref. [91]), and mutations that reduce growth rate occur at a rate of 10^−4^ ∼ 10^−3^ per genome per generation [38, 92]. Moreover, mutation rate can be elevated by as much as 100-fold in hypermutators where DNA repair is dysfunctional [92, 93, 94]. In our simulations, we assume a high, but biologically feasible, rate of 2 × 10^−3^ phenotype-altering mutations per cell per generation per phenotype to speed up computation. At this rate, an average community would sample approximately 20 new mutations per phenotype during maturation. We have also simulated with a 100-fold lower mutation rate. As expected, evolutionary dynamics slowed down, but all of our conclusions still held (S18 Fig).

Among phenotype-altering mutations, around 10% to 40% create null (loss-of-function) mutants, as illustrated by experimental studies on protein, viruses, and yeast [36, 37, 38]. Thus, we assumed that 50% of phenotype-altering mutations were null (i.e., resulting in zero maximal growth rate, zero affinity for metabolite, or zero *f*_*P*_). Among non-null mutations, the relative abundances of enhancing versus diminishing mutations are highly variable in different experiments. It can be impacted by effective population size. For example, with a large effective population size, the survival rate of beneficial mutations is 1,000-fold lower due to clonal interference (competition between beneficial mutations) [95]. The relative abundance of enhancing versus diminishing mutations also strongly depends on the starting phenotype [36, 86, 88]. For example, with ampicillin as a substrate, the wild-type TEM-1 *β*-lactamase is a "perfect" enzyme. Consequently, mutations were either neutral or diminishing, and few enhanced enzyme activity [88]. In contrast, with a novel substrate such as cefotaxime, the enzyme had undetectable activity, and diminishing mutations were not detected, whereas 2% of tested mutations were enhancing [88]. When modeling H-M communities, we assumed that the ancestral H and M had intermediate phenotypes that can be enhanced or diminished.

We based our distribution of mutation effects on experimental studies in which a large number of enhancing and diminishing mutants have been quantified in an unbiased fashion. An example is a study from the Dunham lab in which the fitness effects of thousands of *S*. *cerevisiae* mutations were quantified under various nutrient limitations [39]. Specifically for each nutrient limitation, the authors first measured $\Delta s_{WT}=(w_{WT}-{\bar{w}}_{WT})/{\bar{w}}_{WT}=w_{WT}/{\bar{w}}_{WT}-1$, the deviation in relative fitness of thousands of bar-coded wild-type control strains from the wild-type mean fitness ${\bar{w}}_{WT}$ (i.e., selection coefficients). Due to experimental noise, *Δs*_*WT*_ is distributed with zero mean and nonzero variance. Then, the authors measured thousands of *Δs*_*MT*_, each corresponding to the relative fitness change of a bar-coded mutant strain with respect to the mean of wild-type fitness (i.e., $\Delta s_{MT}=(w_{MT}-{\bar{w}}_{WT})/{\bar{w}}_{WT}$). From these 2 distributions, we used convolution to derive *μ*_*ΔS*_, the probability density function (PDF) of relative fitness change caused by mutations *Δs* = *Δs*_*MT*_ − *Δs*_*WT*_ (S6 Fig), in the following manner.

First, we calculated *μ*_*m*_(*Δs*_*MT*_), the discrete PDF of the relative fitness change of mutant strains, with bin width 0.04. In other words, *μ*_*m*_(*Δs*_*MT*_) = counts in the bin of [*Δs*_*MT*_ − 0.02, *Δs*_*MT*_ + 0.02] ÷ total counts ÷ 0.04 where *Δs*_*MT*_ ranges from −0.6 and 0.6, which is sufficient to cover the range of experimental outcome. The Poissonian uncertainty of *μ*_*m*_ is $\delta \mu_{m}(\Delta s_{MT})=\sqrt{\text{counts}\;\text{per}\;\text{bin}}$ ÷ total counts ÷ 0.04. Repeating this process for the wild-type collection, we obtained the PDF of the relative fitness change of wild-type strains *μ*_*w*_(*Δs*_*WT*_). Next, from *μ*_*w*_(*Δs*_*WT*_) and *μ*_*m*_(*Δs*_*MT*_), we derived *μ*_*Δs*_(*Δs*), the PDF of *Δs* with bin width 0.04:

$$\mu_{\Delta s}(\Delta s=i\times 0.04)=0.04\times \sum_{j=-\infty }^{+\infty }\mu_{w}(j\times 0.04)\mu_{m}((i+j)\times 0.04)$$ (17)

assuming that *Δs*_*MT*_ and *Δs*_*WT*_ are independent from each other. Here, *i* is an integer from −15 to 15. The uncertainty for *μ*_*Δs*_ was calculated by propagation of error. That is, if *f* is a function of *x*_*i*_ (*i* = 1, 2, …, *n*), then *s*_*f*_, the error of *f*, is $s_{f}^{2}=\sum {\left(\frac{\partial f}{\partial x_{i}}s_{x_{i}}\right)}^{2}$, where $s_{x_{i}}$ is the error or uncertainty of *x*_*i*_. Thus,

$$\delta \mu_{\Delta s}(i)=0.04\times \sqrt{\sum_{j}{\left[(\delta \mu_{w}(j)\mu_{m}(i+j)\right)}^{2}+{\left(\mu_{w}(j)\delta \mu_{m}(i+j)\right)}^{2}]}$$ (18)

where *μ*_*w*_(*j*) is short-hand notation for *μ*_*w*_(*Δs*_*WT*_ = *j* × 0.04) and so on. Our calculated *μ*_*Δs*_(*Δs*) with error bar of *δμ*_*Δs*_ is shown in S6 Fig.

Our re-analysis demonstrated that distributions of mutation fitness effects *μ*_*Δs*_(*Δs*) are largely conserved regardless of nutrient conditions and mutation types (S6 Fig). In all cases, the relative fitness changes caused by beneficial (fitness-enhancing) and deleterious (fitness-diminishing) mutations can be approximated by a bilateral exponential distribution with means s_+_ and s_−_ for the positive and negative halves, respectively. After normalizing the total probability to 1, we have

$$\mu_{\Delta s}(\Delta s)=(\begin{array}{cc} \frac{1}{s_{+}+s_{-}(1-e^{-1/s_{-}})}e^{-\Delta s/s_{+}} & \text{if}\Delta s\geq 0\\ \frac{1}{s_{+}+s_{-}(1-e^{-1/s_{-}})}e^{\Delta s/s_{-}} & \text{if}-1<\Delta s<0 \end{array}$$ (19)

We fitted the Dunham lab haploid data (since microbes are often haploid) to Eq 19, using *μ*_*Δs*_(*i*)/*δμ*_*Δs*_(*i*) as the weight for nonlinear least squared regression (green lines in S6 Fig). We obtained s_+_ = 0.050 ± 0.002 and s_−_ = 0.067 ± 0.003.

Exponential distribution described the fitness effects of deleterious mutations in an RNA virus remarkably well [36]. Based on extreme value theory, the fitness effects of beneficial mutations were predicted to follow an exponential distribution [96,97], which has gained experimental support from bacterium and virus [98, 99, 100] (although see [91, 101] for counterexamples). Evolutionary models based on exponential distributions of fitness effects have shown good agreement with experimental data [95, 102].

We have also simulated smaller average mutation effects based on measurements of spontaneous or chemically induced (instead of deletion) mutations. For example, the fitness effects of nonlethal deleterious mutations in *S*. *cerevisiae* were mostly between 1% to 5% [38], and the mean selection coefficient of beneficial mutations in *E*. *coli* was approximately 1% to 2% [95, 98]. Thus, as an alternative, we simulated with s_+_ = s_−_ = 0.02. We obtained the same conclusions (S19A Fig).

### 5. Modeling epistasis on *f*_*P*_

Epistasis, in which the effect of a new mutation depends on prior mutations ("genetic background"), is known to affect evolutionary dynamics. Epistatic effects have been quantified in various ways. Experiments on viruses, bacteria, yeast, and proteins have demonstrated that if 2 mutations were both deleterious or both random, viable double mutants experienced epistatic effects that distributed nearly symmetrically around a value close to zero [103, 104, 105, 106]. In other words, a significant fraction of mutation pairs show no epistasis, and a small fraction show positive or negative epistasis (i.e., a double mutant displays a stronger or weaker phenotype than expected from additive effects of the 2 mutations). Epistasis between 2 beneficial mutations can vary from being predominantly negative [104] to being symmetrically distributed around zero (Fig 2 of [107]). Furthermore, a beneficial mutation tends to confer a lower beneficial effect if the background already has high fitness ("diminishing returns") [107, 108, 109].

A mathematical model by Wiser and colleagues incorporates diminishing-return epistasis [102]. In this model, beneficial mutations of advantage *s* in the ancestral background are exponentially distributed with PDF *αe*^−αs^, where 1/*α >* 0 is the mean advantage. After a mutation with advantage *s* has occurred, the mean advantage of the next mutation would be reduced to 1/[*α*(1 + *gs*)], where *g* > 0 is the "diminishing returns parameter." Wiser and colleagues estimate *g* ≈ 6. This model quantitatively fits the fitness dynamics of evolving *E*. *coli* populations.

Based on the above experimental and theoretical literature, we modeled epistasis on *f*_*P*_ in the following manner. Let the relative mutation effect on current *f*_*P*_ be *Δf*_*P*_ = (*f*_*P*,*mut*_ − *f*_*P*_)/*f*_*P*_ (note *Δf*_*P*_ ≥ −1). Then, *μ*(*Δf*_*P*_, *f*_*P*_), the PDF of *Δf*_*P*_ at the current *f*_*P*_ value, is described by a form similar to Eq 19:

$$\mu (\Delta f_{P},f_{P})=(\begin{array}{cc} \frac{1}{s_{+}(f_{P})+s_{-}(f_{P})(1-e^{-1/s_{-}(f_{P})})}e^{-\Delta f_{P}/s_{+}(f_{P})} & \text{if}\;\Delta f_{P}\geq 0\\ \frac{1}{s_{+}(f_{P})+s_{-}(f_{P})(1-e^{-1/s_{-}(f_{P})})}e^{\Delta f_{P}/s_{-}(f_{P})} & \text{if}-1<\Delta f_{P}<0 \end{array}$$ (20)

Here, *s*_+_(*f*_*P*_) and *s*_−_(*f*_*P*_) are, respectively, the mean *Δf*_*P*_ for enhancing and diminishing mutations at current *f*_*P*_. We assigned *s*_+_(*f*_*P*_) = *s*_+*init*_/(1 + *g* × (*f*_*P*_/*f*_*P*,*init*_ − 1)), where *f*_*P*,*init*_ is the *f*_*P*_ of the initial background in a community selection simulation ($f_{P,init}=f_{P,Mono}^{*}=0.13$), *s*_+*init*_ is the mean enhancing *Δf*_*P*_ occurring in the initial background, and 0 < *g* < 1 is the epistatic factor. Similarly, *s*_−_(*f*_*P*_) = *s*_*−init*_ × (1 + *g* × (*f*_*P*_/*f*_*P*,*init*_ − 1)) is the mean |*Δf*_*P*_| for diminishing mutations at current *f*_*P*_. In the initial background, because *f*_*P*_ = *f*_*P*,*init*_, we have *s*_+_(*f*_*P*_) = *s*_+*init*_ and *s*_−_(*f*_*P*_) = *s*_*−init*_ (*s*_+*init*_ = 0.050 and *s*_*−init*_ = 0.067 in S6 Fig). Consistent with the diminishing returns principle, for subsequent mutations that alter *f*_*P*_, if current *f*_*P*_ > *f*_*P*,*init*_, then a new enhancing mutation became less likely and its mean effect smaller, while a new diminishing mutation became more likely and its mean effect bigger (ensured by *g* > 0; S20 Fig right panel). Similarly, if current *f*_*P*_ < *f*_*P*,*init*_, then a new enhancing mutation became more likely and its mean effect bigger, while a diminishing mutation became less likely and its mean effect smaller (ensured by 0 < *g* < 1; S20 Fig left panel). In summary, our model captured not only diminishing returns epistasis, but also our understanding of mutational effects on protein stability [86].

### 6. Simulation code of community selection

As described in the main text, our simulations tracked the biomass and phenotypes of individual cells as well as the amounts of Resource, Byproduct, and Product in each community throughout community selection. Cell biomass growth, cell division, and changes in chemical concentrations were calculated deterministically. Stochastic processes, including cell death, mutation, and the partitioning of cells of a chosen Adult community into Newborn communities were simulated using the Monte Carlo method.

Specifically, each simulation was initialized with a total of *n*_*tot*_ = 100 Newborn communities with identical configuration:

- Each community had 100 cells of biomass 1. Thus, total biomass *BM*(0) = 100.
- Each community had 40 H cells and 60 M cells with identical *f*_*P*_ and biomass of 1 per cell. Thus, M biomass *M*(0) = 60 and fraction of M biomass *ϕ*_*M*_(0) = 0.6.

Our community selection simulations did not consider mutations arising during pregrowth prior to inoculating Newborns of the first cycle, because incorporating pregrowth had little impact on evolution dynamics (S30 Fig). Thus, initially, all M cells have the same phenotype, and all H cells have the same phenotype.

At the beginning of each selection cycle, a random number was used to seed the random number generator for each Newborn community. This number was saved so that the maturation of each Newborn community can be replayed. In most simulations, the initial amount of Resource was 1 unit of $\overset{\sim }{R}(0)$ unless otherwise specified, the initial Byproduct was *B*(0) = 0, and the initial Product was *P*(0) = 0.

The maturation time *T* was divided into time steps of *Δτ* = 0.05. Resource *R*(*t*) and Byproduct *B*(*t*) during each time interval [*τ*, *τ* + *Δτ*] were calculated by solving the following equations (similar to Eqs 9–10) using the initial condition *R*(*τ*) and *B*(*τ*) via the ode23s solver in Matlab (MathWorks; www.mathworks.com):

$$\frac{dR}{dt}=-c_{RM}g_{M}(R,B)M(\tau )-c_{RH}g_{H}(R)H(\tau )$$ (21)

$$\frac{dB}{dt}=g_{H}(R)H(\tau )-c_{BM}g_{M}(R,B)M(\tau )$$ (22)

where *M*(*τ*) and *H*(*τ*) were the total biomass of M and total biomass of H at time *τ* (treated as constants during time interval [*τ*, *τ* + *Δτ*]), respectively. The solutions from Eqs 21–22 were used in the integrals below to calculate the biomass growth of H and M cells.

Suppose that H and M were rod-shaped organisms with a fixed diameter. Thus, the biomass of an H cell at time *τ* could be written as the length variable *L*_*H*_(*τ*). The continuous growth of *L*_*H*_ between *τ* and *τ* + *Δτ* could be described as

$$\frac{dL_{H}}{dt}=g_{H}(R)L_{H}$$

or

$$\text{ln}\frac{L_{H}(\tau +\Delta \tau )}{L_{H}(\tau )}=\int_{\tau }^{\tau +\Delta \tau }g_{H}(R)dt.$$

Thus,

$$L_{H}(\tau +\Delta \tau )=L_{H}(\tau )\text{exp}\left(\int_{\tau }^{\tau +\Delta \tau }g_{H}(R)dt\right).$$ (23)

Similarly, let the length of an M cell be *L*_*M*_(*τ*). The continuous growth of M could be described as

$$\frac{dL_{M}}{dt}=(1-f_{P})g_{M}(R,B)L_{M}.$$

Thus for an M cell, its length *L*_*M*_(*τ* + *Δτ*) could be described as

$$L_{M}(\tau +\Delta \tau )=L_{M}(\tau )\text{exp}\left(\int_{\tau }^{\tau +\Delta \tau }(1-f_{P})g_{M}(R,B)dt\right).$$ (24)

From Eqs 7–8, within *Δτ* since death is modeled separately, we have

$$\begin{array}{ll} \frac{dP}{dt} & =f_{P}g_{M}\left(R,B\right)M\\ & =\frac{f_{P}}{1-f_{P}}\frac{dM}{dt} \end{array}$$

and therefore

$$P(\tau +\Delta \tau )=P(\tau )+\frac{f_{P}}{1-f_{P}}(M(\tau +\Delta \tau )-M(\tau ))$$ (25)

where *M*(*τ* + *Δτ*) = Σ*L*_*M*_(*τ* + *Δτ*) represented the sum of the biomass (or lengths) of all M cells at *τ* + *Δτ*.

At the end of each *Δτ*, each H and M cell had a probability of *δ*_*H*_*Δτ* and *δ*_*M*_*Δτ* to die, respectively. This was simulated by assigning a random number between 0 and 1 for each cell. Cells assigned with a random number less than *δ*_*H*_*Δτ* or *δ*_*M*_*Δτ* then got eliminated. For surviving cells, if a cell’s length ≥ 2, this cell would divide into 2 cells with half the original length.

After division, each mutable phenotype of each cell had a probability of *P*_*mut*_ to be modified by a mutation (Methods, Section 4). As an example, let’s consider mutations in *f*_*P*_. If a mutation occurred, then *f*_*P*_ would be multiplied by (1 + *Δf*_*P*_), where *Δf*_*P*_ was determined as below.

First, a uniform random number *u*_1_ between 0 and 1 was generated. If *u*_1_ ≤ 0.5, *Δf*_*P*_ = −1, which represented 50% chance of a null mutation (*f*_*P*_ = 0). If 0.5 < *u*_1_ ≤ 1, *Δf*_*P*_ followed the distribution defined by Eq 20 with *s*_+_(*f*_*P*_) = 0.05 for *f*_*P*_-enhancing mutations and *s*_−_(*f*_*P*_) = 0.067 for *f*_*P*_-diminishing mutations when epistasis was not considered (Methods, Section 4). In the simulation, *Δf*_*P*_ was generated via inverse transform sampling. Specifically, *C*(*Δf*_*P*_), the cumulative distribution function (CDF) of *Δf*_*P*_, could be found by integrating Eq 19 from −1 to *Δf*_*P*_:

$$\begin{array}{ll} C\left(\Delta f_{P}\right) & =\int_{-1}^{\Delta f_{P}}\mu_{\Delta s}\left(x\right)dx\\ & =(\begin{array}{cc} \frac{s_{-}}{s_{+}+s_{-}\left(1-e^{-1/s_{-}}\right)}\left(e^{\Delta f_{P}/s_{-}}-e^{-1/s_{-}}\right) & \text{if}\;\Delta f_{P}\leq 0\\ 1-\frac{s_{+}}{s_{+}+s_{-}\left(1-e^{-1/s_{-}}\right)}e^{-\Delta f_{P}/s_{+}} & \text{if}\;\Delta f_{P}\geq 0 \end{array} \end{array}$$ (26)

The 2 parts of Eq 26 overlap at $C(\Delta f_{P}=0)=s_{-}(1-e^{-1/s_{-}})/[s_{+}+s_{-}(1-e^{-1/s_{-}})]$.

In order to generate *Δf*_*P*_ satisfying the distribution in Eq 19, a uniform random number *u*_2_ between 0 and 1 was generated, and we set *C*(*Δf*_*P*_) = *u*_2_. Inverting Eq 26 yielded

$$\Delta f_{P}=(\begin{array}{cc} s_{-}\text{ln}\left(u_{2}(s_{+}+s_{-}(1-e^{-1/s_{-}}))/s_{-}+e^{-1/s_{-}}\right) & u_{2}\leq \frac{s_{-}(1-e^{-1/s_{-}})}{s_{+}+s_{-}(1-e^{-1/s_{-}})}\\ -s_{+}\text{ln}\left((1-u_{2})(s_{+}+s_{-}(1-e^{-1/s_{-}}))/s_{+}\right) & u_{2}>\frac{s_{-}(1-e^{-1/s_{-}})}{s_{+}+s_{-}(1-e^{-1/s_{-}})} \end{array}$$ (27)

When *s*_+_, *s*_−_ ≪ 1, the above equation can be simplified as

$$\Delta f_{P}\approx (\begin{array}{cc} s_{-}\text{ln}\left(u_{2}(s_{+}+s_{-})/s_{-}\right) & u_{2}\leq \frac{s_{-}}{s_{+}+s_{-}}\\ -s_{+}\text{ln}\left((1-u_{2})(s_{+}+s_{-})/s_{+}\right) & u_{2}>\frac{s_{-}}{s_{+}+s_{-}} \end{array}.$$

When epistasis was considered, *s*_+_(*f*_*P*_) = *s*_+*init*_/(1 + *g* × (*f*_*P*_/*f*_*P*,*init*_ −1)) and *s*_−_(*f*_*P*_) = *s*_−*init*_ × (1 + *g* × (*f*_*P*_/*f*_*P*,*init*_ −1)) were used in Eq 27 to calculate *Δf*_*P*_ for each cell (Methods Section 5).

If a mutation increased or decreased the phenotypic parameter beyond its bound (Table 1), the phenotypic parameter was set to the bound value.

The above growth, death, division, and mutation cycle was repeated from time 0 to *T*. Note that because the size of each M and H cell is larger than or equal to 1, the integer numbers of M and H cells, *I*_*M*_ and *I*_*H*_, are generally smaller than the numerical values of biomass *M* and *H*, respectively. At the end of *T*, Adult communities were sorted according to their *P*(*T*) values. The Adult communities with high *P*(*T*) (or a randomly chosen Adult in control simulations in S9 Fig) were chosen for reproduction. Communities were never mixed.

In the top-dog strategy, we started with the Adult with the highest community function. We first calculated the fold by which this Adult would be diluted as $n_{D}=\lfloor \frac{M(T)+H(T)}{BM_{target}}\rfloor$. Here, *BM*_*target*_ = 100 was the preset target for Newborn total biomass, and ⌊*x*⌋ is the floor (round down) function that generates the largest integer that is smaller than *x*. Note that rounding down causes negligible errors. For example, with 7,000 total biomass in an Adult, fold dilution $n_{D}=\frac{7000}{100}=70$ and each Newborn would have an average 100 total biomass. In comparison, with 6999 total biomass in an Adult, *n*_*D*_ = 69 and each Newborn would have an average 101.4 total biomass. This difference of 1% is much smaller than stochastic fluctuations caused by pippetting. If *n*_*D*_ was less than *n*_*tot*_, the total number of communities within a cycle, then all *n*_*D*_ Newborn communities were kept, and the Adult with the next highest function was partitioned to obtain an additional batch of Newborns. From the last batch of Newborns, we would randomly choose enough to obtain *n*_*tot*_ Newborns. The next cycle then began.

During reproduction of the top-tier strategy, *n*_*chosen*_ Adults with the highest functions would each contribute *n*_*tot*_/*n*_*chosen*_ Newborns for the next cycle. We have always picked *n*_*chosen*_ so that *n*_*tot*_/*n*_*chosen*_ is an integer.

Before community reproduction, the current random number generator state was saved so that the random partitioning of Adult communities could be replayed.

To mimic partitioning Adult communities via pipetting into Newborn communities with an average total biomass of *BM*_*target*_, we first calculated the fold of dilution *n*_*D*_ as described above. If the Adult community had *I*_*H*_(*T*) H cells and *I*_*M*_(*T*) M cells, *I*_*H*_(*T*) + *I*_*M*_(*T*) random integers between 1 and *n*_*D*_ were uniformly generated so that each M and H cell was assigned a random integer between 1 and *n*_*D*_. All cells assigned with the same random integer were then assigned to the same Newborn, generating *n*_*D*_ Newborn communities. This partition regimen can be experimentally implemented by pipetting 1/*n*_*D*_ volume of an Adult community into a new well.

To fix *BM*(0) to *BM*_*target*_ and *ϕ*_*M*_(0) to *ϕ*_*M*_(*T*) of the parent Adult (cell sorting), the code randomly assigned M cells from the chosen Adult until the total biomass of M came closest to *BM*_*target*_*ϕ*_*M*_(*T*) without exceeding it. H cells were assigned similarly. Because each M and H cells had a length between 1 and 2, the biomass of M could vary between *BM*_*target*_*ϕ*_*M*_(*T*)−2 and *BM*_*target*_*ϕ*_*M*_(*T*), and the biomass of H could vary between *BM*_*target*_(1 − *ϕ*_*M*_(*T*))−2 and *BM*_*target*_(1 − *ϕ*_*M*_(*T*)). Variations in *BM*(0) and *ϕ*_*M*_(0) were sufficiently small so that community selection worked (Fig 3D and 3E). We also simulated sorting cells such that H and M cell numbers (instead of biomass) were fixed in Newborns. Specifically, ⌊*BM*_*target*_*φ*_*M*_(*T*)/1.5⌋ M cells and ⌊*BM*_*target*_(1 − *φ*_*M*_(*T*))/1.5⌋ H cells were sorted into each Newborn community, where we assumed that the average biomass of a cell was 1.5, and *φ*_*M*_(*T*) = *I*_*M*_(*T*)/(*I*_*M*_(*T*) + *I*_*H*_(*T*)) was calculated from cell numbers in the parent Adult community. We obtained the same conclusion (S13C Fig).

To fix Newborn total biomass *BM*(0) to the target total biomass *BM*_*target*_ while allowing *ϕ*_*M*_(0) to fluctuate (S12 Fig first and third columns), H and M cells were randomly assigned to a Newborn community until *BM*(0) came closest to *BM*_*target*_ without exceeding it (otherwise, *P*(*T*) might exceed the theoretical maximum). For example, suppose that a certain number of M and H cells had been sorted into a Newborn so that the total biomass was 98.6. If the next cell, either M or H, had a biomass of 1.3, this cell would go into the community so that the total biomass would be 98.6 + 1.3 = 99.9. However, if a cell of mass 1.6 happened to be picked, this cell would not go into this community so that this Newborn had a total biomass of 98.6 and the cell of mass 1.6 would go to the next Newborn. Thus, each Newborn might not have exactly the biomass of *BM*_*target*_, but rather between *BM*_*target*_ −2 and *BM*_*target*_. Experimentally, total biomass can be determined from the optical density, or from the total fluorescence if cells are fluorescently labeled [61]. To fix the total cell number (instead of total biomass) in a Newborn, the code randomly assigned a total of ⌊*BM*_*target*_/1.5⌋ cells into each Newborn, assuming an average cell biomass of 1.5. We obtained the same conclusion, as shown in S13A Fig.

To fix *ϕ*_*M*_(0) to *ϕ*_*M*_(*T*) of the chosen Adult community from the previous cycle while allowing *BM*(0) to fluctuate (S12 Fig second and fourth columns), the code first calculated dilution fold *n*_*D*_ in the same fashion as mentioned above. If the Adult community had *I*_*H*_(*T*) H cells and *I*_*M*_(*T*) M cells, *I*_*M*_(*T*) random integers between [1, *n*_*D*_] were then generated for each M cell. All M cells assigned the same random integer joined the same Newborn community. The code then randomly dispensed H cells into each Newborn until the total biomass of H came closest to *M*(0)(1 − *ϕ*_*M*_(*T*))/*ϕ*_*M*_(*T*) without exceeding it, where *M*(0) was the biomass of all M cells in this Newborn community. Again, because each M and H had a biomass (or length) between 1 and 2, *ϕ*_*M*_(0) of each Newborn community might not be exactly *ϕ*_*M*_(*T*) of the chosen Adult community. We also performed simulations in which the ratio between M and H cell numbers in the Newborn community, *I*_*M*_(0)/*I*_*H*_(0), was set to *I*_*M*_(*T*)/*I*_*H*_(*T*) of the parent Adult community and obtained the same conclusion (S13B Fig).

### 7. Problems associated with an alternative definition of community function and an alternative method of reproducing Adults

Here, we describe problems associated with an alternative definition of community function and an alternative method of community reproduction.

An alternative definition of community function is Product per M biomass in an Adult community: *P*(*T*)/*M*(*T*). To illustrate problems with this definition, let’s calculate *P*(*T*)/*M*(*T*) assuming that cell death is negligible. From Eqs 7 and 8,

$$\frac{dM}{dt}=(1-f_{P})g_{M}M$$

$$\frac{dP}{dt}=f_{P}g_{M}M$$

where biomass growth rate *g*_*M*_ is a function of *B* and *R*. Thus,

$$\frac{dM}{(1-f_{P})dt}=\frac{dP}{f_{P}dt}$$

and we have

$$P(T)=\frac{f_{P}}{1-f_{P}}(M(T)-M(0))\approx \frac{f_{P}}{1-f_{P}}M(T)$$

if *M*(*T*) ≫ *M*(0) (true if *T* is long enough for cells to double at least 3 or 4 times). In this case,

$$P(T)\approx \frac{f_{P}}{1-f_{P}}M(0)\text{exp}\left((1-f_{P})\int_{T}g_{M}dt\right)$$ (28)

If we define community function as $P(T)/M(T)\approx \frac{f_{P}}{1-f_{P}}$, then higher community function requires higher $\frac{f_{P}}{1-f_{P}}$ or higher *f*_*P*_. However, if we select for very high *f*_*P*_, then M can go extinct (Fig 2 and S2 Fig).

In our community selection scheme, the average total biomass of Newborn communities was set to a constant *BM*_*target*_. Alternatively, each Adult community can be partitioned into a constant number of Newborn communities (i.e., fixed-fold dilution). If Resource is not limiting, then there is no competition between H and M, and *P*(*T*) increases as *M*(0) and *H*(0) increase. Therefore, selection for higher *P*(*T*) results in selection for higher Newborn total biomass (instead of higher *f*_*P*_, S23 Fig). This will continue until Resource becomes limiting, and then communities will get into the stationary phase.

### 8. $f_{P}^{*}$ is smaller for M group than for H-M community

For groups or communities with a certain *∫*_*T*_ *g*_*M*_*dt*, we can calculate *f*_*P*_ optimal for community function from Eq 28 by setting

$$\frac{dP(T)}{df_{p}}\approx M(0)\frac{d}{df_{p}}\left[\frac{f_{P}}{1-f_{P}}\text{exp}\left((1-f_{P})\int_{T}g_{M}dt\right)\right]=0.$$

We have

$$\frac{1}{(1-f_{P}{)}^{2}}\text{exp}\left((1-f_{P})\int_{T}g_{M}dt\right)-\frac{f_{P}}{1-f_{P}}\int_{T}g_{M}dt\text{exp}\left((1-f_{P})\int_{T}g_{M}dt\right)=0$$

or

$$1/\int_{T}g_{M}dt=f_{P}(1-f_{P}).$$

If *∫*_*T*_ *g*_*M*_*dt* ≫ 1, *f*_*P*_ is very small, then the optimal *f*_*P*_ for *P*(*T*) is

$$f_{P}^{*}\approx {\left(\int_{T}g_{M}dt\right)}^{-1}.$$ (29)

*∫*_*T*_ *g*_*M*_*dt* is larger in monoculture than in community because M grows faster in monoculture than in community. This is because B is supplied in excess in monoculture, whereas in community, H-supplied Byproduct is initially limiting. Thus, according to Eq 29, $f_{P}^{*}\approx 1/\int_{T}g_{M}dt$ is smaller for monoculture than for community.

### 9. Stochastic fluctuations during community reproduction

The total number of cells in a Newborn community is approximately $BM(0)/\bar{L}$, where $\bar{L}$ is the average biomass (or length) of M and H cells. This number fluctuates in a Poissonian fashion with a standard deviation of $\sqrt{BM_{target}/\bar{L}}$. As a result, the biomass of a Newborn communities fluctuates around *BM*_*target*_ with a standard deviation of $\sqrt{BM_{target}/\bar{L}}\times \bar{L}=\sqrt{BM_{target}\bar{L}}$.

Similarly, *M*(0) and *H*(0) fluctuate independently with a standard deviation of $\sqrt{E[M(0)]\bar{L}}=\sqrt{BM_{target}\phi_{M}(T)\bar{L}}$ and $\sqrt{E[H(0)]\bar{L}}=\sqrt{BM_{target}(1-\phi_{M}(T))\bar{L}}$, respectively, where “E” means the expected value and *ϕ*_*M*_(*T*) is the fraction of M biomass in the parent Adult community. Therefore, *M*(0)/*H*(0) fluctuates with a variance of

$$\text{Var}[M(0)/H(0)]={\left(\frac{E[M(0)]}{E[H(0)]}\right)}^{2}\left[\frac{\text{Var}[M(0)]}{(E[M(0)]{)}^{2}}-2\frac{\text{Cov}[M(0),H(0)]}{E[M(0)]E[H(0)]}+\frac{\text{Var}[H(0)]}{(E[H(0)]{)}^{2}}\right]$$

$$=\frac{\phi_{M}(T)}{(1-\phi_{M}(T){)}^{3}}\frac{\bar{L}}{BM_{target}}$$

where "Cov" means covariance (equal to zero) and "Var" means variance.

### 10. Mutualistic H-M community

In the mutualistic H-M community, Byproduct inhibits the growth of H. According to Luli and colleagues [110], the growth rate of *E*. *coli* decreases exponentially as the exogenously added acetate concentration increases. Thus, we only need to modify the growth of H by a factor of exp(−*B*/*B*_0_) where *B* is the concentration of Byproduct and *B*_0_ is the concentration of Byproduct at which H’s growth rate is reduced by *e*^−1^~0.37:

$$\frac{dH}{dt}=\text{exp}\left(-\frac{B}{B_{0}}\right)\frac{g_{Hmax}R}{R+K_{HR}}H-\delta_{H}H.$$

The larger *B*_0_, the less inhibitory effect Byproduct has on H, and when *B*_0_ → +∞, Byproduct does not inhibit the growth of H. For simulations in S22 Fig, we set *B*_0_ = 2*K*_*MB*_.

## Supporting information

S1 Fig Artificial selection is more challenging for multispecies communities than for individuals or monospecies groups. Artificial selection can be applied to any population of entities [111]. An entity can be an individual (A), a monospecies group (B), or a multispecies community (C). Unlike natural selection, which selects for fastest-growing cells, artificial selection generally selects for traits that are costly to individuals. In each selection cycle, a population of "Newborn" entities grow for maturation time *T* to become "Adults." Adults expressing a higher level of the trait of interest (darker shade) are chosen by the experimentalist to reproduce. An individual reproduces by making copies of itself, while an Adult group or community can reproduce by randomly splitting into multiple Newborns of the next selection cycle. Successful artificial selection requires that (i) entities display trait variations; (ii) trait variations can be selected to result in differential entity survival and reproduction; and (iii) entity trait is sufficiently heritable from one selection cycle to the next [112]. In all 3 types of selection, entity variations can be introduced by mutations and recombinations in individuals. However, heredity can be low in community selection. (A) Artificial selection of individuals has been successful [21, 22, 23, 113], since a trait is largely heritable so long as mutation and recombination are sufficiently rare. (B, C) In group selection and community selection, if maturation time *T* is small so that newly arising genotypes cannot rise to high frequencies within a selection cycle, then Adult trait is mostly determined by Newborn composition (the biomass of each genotype in each member species). In this case, variation can be defined as the dissimilarity in Newborn compositions within a selection cycle, while heredity can be defined as the similarity of compositions between Newborns connected through lineage across consecutive selection cycles (tubes with same-colored outlines in Fig 4A). (B) Artificial selection of monospecies groups has been successful [18, 46, 48]. Suppose cooperators but not cheaters pay a fitness cost to generate a product (dark shade). Artificial selection for groups producing high total product favors cooperator-dominated groups, although within a group, cheaters grow faster than cooperators. If a Newborn group has a large number of cells (top), all Newborns will harbor similar fractions of cheaters, and thus intergroup variation will be small [71]. During maturation, cheater frequency will increase, thereby diminishing heredity. Low variation and low heredity interfere with group selection. In contrast, when Newborn groups are initiated at a small size such as one individual (bottom), a Newborn group will comprise either a cooperator or a cheater, thereby ensuring variation. Furthermore, even if cheaters were to arise during maturation of the group, a fraction of Newborns of the next cycle will by chance inherit a cooperator, thereby ensuring some level of heredity. Thus, group selection can work when Newborn size is small. (C) Artificial selection of multispecies communities may be hindered by insufficient heredity. During maturation, the relative abundance of genotypes and species can rapidly change due to ecological interactions and evolution, which compromises heredity. During community reproduction, stochastic fluctuations in Newborn species and genotype composition further reduce heredity.
(TIFF)

S2 FigProblems of defining community function as *P*(*T*)/*M*(*T*).When community function was defined by *P*(*T*)/*M*(*T*), average *f*_*P*_ of the chosen communities rapidly increased to such a high level that M was outcompeted by H, as demonstrated by Fig 2A bottom panel. Consequently, selection abruptly came to a stop. Black, cyan, and gray curves are independent simulation trials. $\bar{P}(T)$ was averaged across chosen Adults. ${\bar{f}}_{P}(T)$ was obtained by first averaging among M within each chosen Adult, and then averaging across all chosen Adults. The simulation codes can be found in S5 Code, and the data can be found in S4 Data.(TIF)

S3 Fig Growth models of H and M. (A) *H* growth follows Monod kinetics, reaching half maximal growth rate when *R* = *K*_*HR*_. (B) *M* growth follows dual-substrate Mankad-Bungay kinetics. When Resource *R* is in great excess (*R*_*M*_ ≫ *B*_*M*_) or Byproduct *B* is in great excess (*B*_*M*_ ≫ *R*_*M*_), we recover monosubstrate Monod kinetics (panel A). Here, for simplicity, symbols represent absolute quantities.
(TIF)

S4 FigA comparison of dual-substrate models.Suppose that cell growth rate depends on each substrate S_1_ and S_2_ in a Monod-like, saturable fashion. When S_2_ is in excess, the *S*_1_ at which half maximal growth rate is achieved is *K*_1_. When S_1_ is in excess, the *S*_2_ at which half maximal growth rate is achieved is *K*_2_. (A) In the "Double Monod" model, growth rate depends on the 2 limiting substrates in a multiplicative fashion. (B) In the model proposed by Mankad and Bungay, growth rate takes a different form. In both models, when one substrate is in excess, growth rate depends on the other substrate in a Monod-like fashion. However, when $\frac{S_{1}}{K_{1}}=\frac{S_{2}}{K_{2}}=1$, the growth rate is predicted to be *g*_*max*_/2 by the Mankad and Bunday model, and *g*_*max*_/4 by the Double Monod model. The Mankad and Bungay model outperforms the Double Monod model in describing experimental data of *S*. *cerevisiae* and *E*. *coli* growing on low glucose and low nitrogen. The figures are plotted using data from reference [30].(TIF)

S5 Fig A comparison of different simulations of exponential cell growth in excess metabolites. Thick black line: analytical solution with biomass growth rate (0.7/time unit). Gray dashed line: simulation assuming that biomass increases exponentially at 0.7/time unit and that cell division occurs upon reaching a biomass threshold, an assumption used in our model. Colored dotted lines: simulations assuming that cell birth is discrete and occurs at a probability equal to the birth rate multiplied with the length of simulation time step (Δ*τ* = 0.05 time unit). When a cell birth occurs, biomass increases discretely by 1, resulting in step-wise increase in colored dotted lines at early time. The Matlab codes can be found in S6 Code.
(TIF)

S6 Fig PDFs of changes in relative fitness due to mutations (*μ*_*Δs*_(*Δs*)). We derived ***μ***_***Δs***_(***Δs***) from the Dunham lab data [39] where bar-coded mutant strains were competed under sulfate limitation (red), carbon limitation (blue), or phosphate limitation (black). Error bars represent uncertainty *δμ*_*Δs*_ (the lower error bar is omitted if the lower estimate is negative). In the leftmost panel, green lines show nonlinear least squared fitting of data to Eq 19 using all 3 sets of data. Note that data with larger uncertainty are given less weight and thus deviate more from the fitting. For an exponentially distributed PDF *p*(*x*) = exp(−*x*/*r*)/*r* where *x*, *r* > 0, and the average of *x* is *r*. When plotted on a semi-log scale, we get a straight line with slope −1/*r*, which gets us the average effect *r*. From the green line on the right side, we obtain the average effect of enhancing mutations *s*_+_ = 0.050 ± 0.002, and from the green line on the left side, we obtain the average effect of diminishing mutations *s*_−_ = 0.067 ± 0.003. The probability of a mutation altering a phenotype by ±*α* is the area of the hatched region drawn in the leftmost panel. The Matlab codes can be found in S7 Code. PDF, probability density function.
(TIF)

S7 Fig Large Newborn group size or long maturation time allows noncontributors to accumulate to similar levels in all groups, thereby reducing intergroup variation. For simplicity, we modeled the growth of Newborn groups of M cells. Both wild-type and mutant cells followed exponential growth. The growth rate of wild-type cells was 0.87 times that of mutants. From a Newborn biomass *BM*(0) of 10^2^ (top panels) or 10^4^ (bottom panels) wild-type M cells, M population multiplied for 6 (left panels) or 100 (right panels) generations. Immediately following cell division, wild-type daughter cells mutated to noncontributors with a probability of 10^−3^. The fraction of biomass made up by mutants at each wild-type doubling is shown. The simulation codes can be found in S8 Code.
(TIF)

S8 FigImproved individual growth can promote community function.Here, we allowed mutations to alter M’s *f*_*P*_ and H and M’s growth parameters (Table 1). Communities were chosen using the top-dog strategy. (A–C) Community reproduction via pipetting (i.e., Newborn biomass and species composition can fluctuate). Community function *P*(*T*) increased upon community selection (A). Since *f*_*P*_ remained unchanged (panel B), this increase in *P*(*T*) must be due to improved growth parameters (panel C). (D–F) Community reproduction via biomass sorting (i.e., fixed Newborn total biomass and species composition). Community function improved to a much higher level (panel D). In both strategies, the 5 growth parameters increased to their respective evolutionary upper bounds (green dashed lines). Magenta dashed lines: optimal *f*_*P*_ for community function and maximal community function *P*(*T*) when all 5 growth parameters are fixed at their evolutionary upper bounds and *ϕ*_*M*_(0) is also optimal for *P*(*T*). Black, cyan, and gray curves show independent simulations. $\bar{P}(T)$ is averaged across chosen Adults. ${\bar{g}}_{Mmax},{\bar{g}}_{Hmax}$, and ${\bar{f}}_{P}$ are obtained by averaging within each chosen Adult and then averaging across chosen Adults. *K*_*SpeciesMetabolite*_ are averaged within each chosen Adult, then averaged across chosen Adults, and finally inverted to represent average affinity. Note different horizontal axis scales. The maximal growth rates (*g*_*Mmax*_ and *g*_*Hmax*_) have the unit of 1/time. Affinity for Resource (1/*K*_*MR*_, 1/*K*_*HR*_) has the unit of $1/\tilde{R}(0)$, where $\tilde{R}(0)$ is the initial amount of Resource in Newborn. Affinity for Byproduct (1/*K*_*MB*_) has the unit of $1/{\tilde{r}}_{B}$, where ${\tilde{r}}_{B}$ is the amount of Byproduct released per H biomass produced. Product P has the unit of ${\tilde{r}}_{P}$, the amount of Product released at the cost of 1 M biomass. More details on parameters and variables can be found in Tables 1 and 2. The simulation codes can be found in S9 Code, and the data can be found in S5 Data.(TIF)

S9 FigCommunity function declines to zero in the absence of intercommunity selection for higher community function.An Adult was randomly chosen to reproduce as many Newborns as possible, and a second Adult was randomly chosen to reproduce more Newborns until 100 Newborns were obtained. Natural selection favored zero *f*_*P*_ (panel A). Consequently, *P*(*T*) decreased to zero (panel B). Here, reproduction was done by pipetting. Black, cyan, and gray curves are independent simulation trials. $\bar{P}(T)$ was averaged across the randomly chosen Adults. ${\bar{f}}_{P}(T)$ was obtained by first averaging among M within each randomly chosen Adult and then averaging across the chosen Adults. The simulation codes can be found in S10 Code, and the data can be found in S6 Data.(TIF)

S10 FigP*(*T*) is a local optimum because it cannot be further improved by small changes.We started each Newborn community with total biomass *BM*(0) = 100, all 5 growth parameters at their evolutionary upper bounds, and $f_{P}^{*}=0.41$ and $\phi_{M}^{*}(0)=0.54$ to achieve P*(*T*). We then allowed all 5 growth parameters and *f*_*P*_ to mutate while applying community selection. To ensure effective community selection (Fig 3D–3F), the strategy of top-dog with cell sorting was implemented. We found that all 5 growth parameters remained at their respective evolutionary upper bounds. At the end of the first cycle (Cycle = 1 in insets), even though ${\bar{f}}_{P}$ did not change, $\bar{P}(T)$ had already declined from the original magenta dashed line. This is because species interactions have driven *ϕ*_*M*_(0) from the optimal $\phi_{M}^{*}(0)$ (= 0.54) to near the steady-state value (*ϕ*_*M*_ = 0.64, compare with *ϕ*_*M*,*SS*_ represented by the blue dashed line in Fig 2A top panel). Later, over hundreds of cycles, ${\bar{f}}_{P}$ gradually increased, while $\bar{P}(T)$ was still below maximal. This is because species composition gravitated toward steady-state *ϕ*_*M*,*SS*_, which deviated from the optimal $\phi_{M}^{*}(0)$. Other legends are the same as S8 Fig. The simulation codes can be found in S11 Code, and the data can be found in S7 Data.(TIF)

S11 Fig Variations in community function can arise from nonheritable variations in Newborn compositions. An average Newborn community (solid lines) has a total biomass of 100 with 75% M. (A) A "lucky" Newborn community (dotted lines), by stochastic fluctuations, has a higher total biomass of 130 and the average 75% M. Even though the lucky and the average communities share identical *f*_*P*_ = 0.1, biomass of M in the lucky Newborn can grow to a higher value (left), deplete more Resource (middle), and make more Product (right) by the end of short *T* (*T* = 17). (B) A "lucky" Newborn community (dotted lines), by stochastic fluctuations, has 65% (instead of 75%) M and the average total biomass of 100. Even though the lucky and the average communities share identical *f*_*P*_ = 0.1, higher fraction of Helper H biomass in the lucky community results in faster accumulation of Byproduct. Consequently, M can enjoy a shorter growth lag, grow to a larger size (left), deplete more Resource (middle), and make more Product (right) by the end of short *T* (*T* = 17). In both cases, the difference between the lucky (dotted) and the average (solid) communities diminished at longer *T* (*T* = 20) compared to shorter *T* (*T* = 17, dash dot line). The Matlab codes can be found in S12 Code.
(TIF)

S12 FigFixing either Newborn total biomass or Newborn species composition did not significantly improve community selection compared to fixing neither.When using the top-dog strategy (A) or the top 10% strategy (B), fixing only Newborn total biomass (*BM*(0)) or fixing only Newborn species composition (*ϕ*_*M*_(0)) yielded similar dynamics as fixing neither (pipetting; Fig 3A–3C and Fig 3G–3I, respectively). Black, cyan, and gray curves are 3 independent simulation trials. $\bar{P}(T)$ was averaged across all chosen Adults. ${\bar{f}}_{P}(T)$ was obtained by first averaging among M within each chosen Adult and then averaging across all chosen Adults. The simulation codes can be found in S2 Code, and the data can be found in S8 Data.(TIF)

S13 FigFixing H and M cell numbers during community reproduction improves community function.(A) The total cell number in Newborn communities was fixed to ⌊*BM*_*target*_/1.5⌋ where ⌊*x*⌋ means rounding down *x* to the nearest integer. (B) The ratio between M and H cell numbers in Newborn communities were fixed to *I*_*M*_(*T*)/*I*_*H*_(*T*), where *I*_*M*_(*T*) and *I*_*H*_(*T*) were the number of M and H cells in the chosen Adult community from the previous cycle, respectively. (C) The total cell numbers of Newborn communities were fixed to ⌊*BM*_*target*_/1.5⌋, and the ratio between M and H cell numbers were fixed to *I*_*M*_(*T*)/*I*_*H*_(*T*). See Methods Section 6 for details of simulating community reproduction. Black, cyan, and gray curves are independent simulation trials. $\bar{P}(T)$ was averaged across the 2 chosen Adults. ${\bar{f}}_{P}(T)$ was obtained by first averaging among M within each chosen Adult and then averaging across the 2 chosen Adults. The simulation codes can be found in S13 Code, and the data can be found in S9 Data.(TIF)

S14 FigLong-term selection dynamics of the top 10% strategy with pipetting.Simulation setup was identical to that in Fig 3G–3H, except that selection here lasted more cycles. Compared to Fig 3A–3C (top-dog, pipetting), the top 10% strategy was more effective. However, compared to Fig 3D–3F (top-dog, cell sorting), the top 10% strategy was less effective, even over 10^4^ cycles. Black, cyan, and gray curves are independent simulation trials. $\bar{P}(T)$ was averaged across the chosen Adults. ${\bar{f}}_{P}(T)$ was obtained by first averaging among M within each chosen Adult and then averaging across all chosen Adults. The simulation codes can be found in S2 Code, and the data can be found in S1 Data.(TIF)

S15 FigTop-tier strategies promoted community selection under a wide range of selection strengths.In a top-tier strategy (“top 2%” to “top 50%”), top *n*_*chosen*_(= 2~50) Adults each contributed 100/*n*_*chosen*_ Newborns into the next cycle. Here, Adults were reproduced (split) into Newborns as if via pipetting. Note that “top 2%” yielded qualitatively similar results as “top-dog”. Note also that when all Adults contributed one Newborn each (“all 100%”), intercommunity selection strength was zero, and thus natural selection quickly reduced average cost *f*_*P*_ and community function to zero. Black, cyan, and gray curves are independent simulation trials. $\bar{P}(T)$ was averaged across the chosen Adults. ${\bar{f}}_{P}(T)$ was obtained by first averaging among M within each chosen Adult and then averaging across all chosen Adults. The simulation codes can be found in S2 Code, and the data can be found in S10 Data.(TIF)

S16 Fig The top-dog strategy is superior to the top 10% strategy when nonheritable variation in community function is low. Twenty replicas of selection simulations were performed using either the top-dog strategy (black curves) or the top-tier strategy (top 10 Adults chosen to reproduce; red curves). Community reproduction was through cell sorting. Community functions improved slightly faster and to a slightly higher level using the top-dog strategy. Thus, when nonheritable variations in community function were suppressed, the top-dog strategy was superior to the top-tier strategy. The simulation codes can be found in S2 Code, and the data can be found in S11 Data.
(TIF)

S17 FigCommunity function can be improved even if it is costly to both species.Identical to Fig 6, the evolutionary upper bound for *g*_*Hmax*_ ($g_{Hmax}^{*}=0.8$) was larger than that of *g*_*Mmax*_ ($g_{Mmax}^{*}=0.7$), opposite to that in Fig 3. (A) When using the top-dog strategy with pipetting, *g*_*Hmax*_ and *g*_*Mmax*_ evolved to their respective upper bounds, and thus *g*_*Hmax*_ > *g*_*Hmax*_ (compare i and iv). This would ordinarily lead to extinction of M. However, community selection managed to maintain M at a very low level (Fig 6A bottom panel). (B–D) When using the top-dog strategy with cell sorting (panel B), the top 10% strategy with pipetting (panel C), or the top 10% strategy with cell sorting (panel D), community selection worked in the sense that both ${\bar{f}}_{P}$ and *P*(*T*) improved over cycles (Fig 6B–6D). The maximal growth rate of H *g*_*Hmax*_ did not increase to its upper bound $g_{Hmax}^{*}=0.8$, and H’s affinity for Resource even decreased from the ancestral level in some cases. Here, Resource supplied to Newborn communities could support 10^5^ total biomass to accommodate faster growth rate. Other legends are the same as S8 Fig. The simulation codes can be found in S4 Code, and the data can be found in S3 Data.(TIF)

S18 FigEvolution dynamics of chosen Adult communities at a mutation rate of 2 × 10^−5^ per cell per generation.(A, B) At short maturation time (*T* = 17, Resource was not exhausted in an average community), cell sorting improved community function. The top-tier strategy with pipetting slightly improved community function. (C, D) At long maturation time (*T* = 20, Resource was nearly exhausted in an average community), community function improved without fixing *BM*(0) or *ϕ*_*M*_(0) (top-dog with pipetting). At this mutation rate, because the population size of a community never exceeds 10^4^, a mutation occurs on average every 5 cycles, resulting in step-wise improvement in both ${\bar{f}}_{P}(T)$ and $\bar{P}(T)$. Black, cyan, and gray curves are independent simulation trials. $\bar{P}(T)$ was averaged across all chosen Adults. ${\bar{f}}_{P}(T)$ was obtained by first averaging among M within each chosen Adult and then averaging across all chosen Adults. The simulation codes can be found in S2 Code, and the data can be found in S12 Data.(TIF)

S19 FigEvolutionary dynamics of chosen Adult communities under different distributions of mutation effects.(A) Evolutionary dynamics where half of the mutations reduced *f*_*P*_ to zero, and the distribution of mutation effects of the other half is specified by Eq 19 in which *s*_+_ = *s*_−_ = 0.02 are constants. (B) Evolutionary dynamics when null mutations in *f*_*P*_ did not occur. The distribution of mutation effects is specified by Eq 19 where *s*_+_ = 0.05 and *s*_−_ = 0.067. ${\bar{f}}_{P}(T)$ as well as $\bar{P}(T)$ were more stable compared to when null mutations were present (Fig 3). Black, cyan, and gray curves are independent simulation trials. $\bar{P}(T)$ was averaged across the chosen Adults. ${\bar{f}}_{P}(T)$ was obtained by first averaging among M within each chosen Adult and then averaging across all chosen Adults. For panel A, the simulation codes can be found in S14 Code, and the data can be found in S13 Data. For panel B, the simulation codes can be found in S15 Code, and the data can be found in S14 Data.(TIF)

S20 Fig Mutation effects under epistasis. Distribution of mutation effects at different current *f*_*P*_ values (marked on top) are plotted according to Eq 20. (Top) When there is no epistasis, distribution of mutational effects on *f*_*P*_ (*Δf*_*P*_) remains identical regardless of current *f*_*P*_. (Middle and Bottom) With epistasis (see Methods Section 5 for definition of epistasis factor), mutational effects on *f*_*P*_ depend on the current value of *f*_*P*_. If current *f*_*P*_ is low (left), enhancing mutations are more likely to occur (the area to the right of *Δf*_*P*_ = 0 becomes bigger), and their mean mutational effect (= 1/slope) becomes larger. If current *f*_*P*_ is high (right), the opposite is true.
(TIF)

S21 FigEvolutionary dynamics of chosen Adults when epistasis is considered.When we incorporated different epistasis strengths (epistasis factor of 0.3 and 0.8), we obtained essentially the same conclusions as when epistasis was not considered (Fig 3). Black, cyan, and gray curves are independent simulation trials. $\bar{P}(T)$ was averaged across the chosen Adults. ${\bar{f}}_{P}(T)$ was obtained by first averaging among M within each chosen Adult and then averaging across all chosen Adults. For panel A, the simulation codes can be found in S16 Code, and the data can be found in S15 Data. For panel B, the simulation codes can be found in S16 Code, and the data can be found in S16 Data.(TIF)

S22 FigSelection dynamics of mutualistic H-M communities.In the mutualistic H-M community, H generates Byproduct that is essential for M but inhibitory to H. (A) H can grow to a high density in the presence of M (top) but not in the absence of M (bottom). (B) Similar to community selection on commensal H-M communities, selection was promoted by the top 10% strategy or cell sorting at short *T* (*T* = 20), or via extending *T* (*T* = 24). Black, cyan, and gray curves are independent simulation trials. $\bar{P}(T)$ was averaged across the chosen Adults. ${\bar{f}}_{P}(T)$ was obtained by first averaging among M within each chosen Adult and then averaging across all chosen Adults. For panel A, the Matlab codes can be found in S17 Code. For panel B, the simulation codes can be found in S18 Code, and the data can be found in S17 Data.(TIF)

S23 FigArtificial selection in excess Resource failed under fixed-fold pipetting dilution scheme.Excess Resource was supplied to each Newborn (*R*(0)/*K*_*MR*_ = 10^6^), and chosen Adults were reproduced via a fixed-fold (100-fold) pipetting dilution into Newborns. Because of pipetting, Newborns with larger total biomass will tend to be selected (Fig 4F). Community function initially increased as Newborn total biomass increased exponentially (middle and bottom panels), while nonproducing M cells with *f*_*P*_ = 0 quickly took over (top panel; S7B Fig). Black, cyan, and gray curves are independent simulation trials. $\bar{P}(T)$ was averaged across chosen Adults. ${\bar{f}}_{P}(T)$ was obtained by first averaging among M within each chosen Adult and then averaging across all chosen Adults. The simulation codes can be found in S19 Code, and the data can be found in S18 Data.(TIF)

S24 FigImproving growth parameters can improve or impair community function, depending on evolutionary upper bounds of growth parameters.Plotted here are plateaued community function after 1,500 cycles when simulation did or did not allow mutations in growth parameters or *f*_*P*_. The top-dog strategy and pipetting were used. (A) When evolutionary upper bound for *g*_*Hmax*_ ($g_{Hmax}^{*}=0.3$) was lower than that of *g*_*Mmax*_ ($g_{Mmax}^{*}=0.7$), improving growth parameters improved community function. Compared to community function where no mutations were allowed (i), community function improved when both growth parameters and *f*_*P*_ were allowed to mutate (ii). Preventing mutations in growth parameters diminished community function improvement (iii). In this case, improved growth of M and H resulted in higher community function. Evolutionary dynamics are shown in S8A–S8C Fig. (B) When evolutionary upper bound for *g*_*Hmax*_ ($g_{Hmax}^{*}=0.8$) was larger than that of *g*_*Hmax*_ ($g_{Mmax}^{*}=0.7$), improving growth parameters could decrease community function. Compared to community function in which no mutations were allowed (i), community function decreased when both growth parameters and *f*_*P*_ were allowed to mutate (ii). Preventing mutations in growth parameters diminished reduction in community function (iii). In this case, improved growth of M and H resulted in lower community function. Evolutionary dynamics are shown in S17A Fig and Fig 6A. In panel B, Resource supplied to Newborn communities could support 10^5^ total biomass to accommodate faster growth rate. Error bars are calculated form 3 independent selections. The simulation codes can be found in S2 and S4 Codes. The plot can be generated by S20 Code from S19 Data.(TIF)

S25 FigSelection dynamics of M monospecies groups.(A) Phenotypes averaged over chosen groups are plotted for 500 selection cycles. Because Resource is the same as in community selection while Byproduct is in excess, M’s affinity for Byproduct 1/*K*_*MB*_ is no longer relevant in equations (S3B Fig, *R*_*M*_ ≪ *B*_*M*_). Upper bounds of M’s maximal growth rate *g*_*Mmax*_ and M’s affinity for Resource 1/*K*_*MR*_ are marked with green dashed lines. Magenta lines mark *f*_*P*_ optimal for group function and maximal *P*(*T*) when M’s maximal growth rate *g*_*Mmax*_ and M’s affinity for Resource 1/*K*_*MR*_ are fixed at their upper bounds and when Byproduct is in excess. (B) Suppose that a Newborn M group starts with a single Manufacturer (biomass 1) supplied with excess Byproduct and the same amount of Resource as in a Newborn H-M community (Resource could support 10^4^ M biomass). Then, maximal group function is achieved at $f_{P}=f_{P,Mono}^{*}=0.13$ (middle panel), lower than the optimal *f*_*P*_ for the community function $f_{P}^{*}=0.41$ (Fig 2B). Here, the growth parameters of M are all fixed at their evolutionary upper bounds, and *P*(*T*) has the unit of ${\tilde{r}}_{P}$. For panel A, the simulation codes can be found in S21 Code, and the data can be found in S20 Data. For panel B, the Matlab codes can be found in S22 Code.(TIF)

S26 Fig Comparison between the steady-state *ϕ*_*M*_,_*SS*_ calculated from Eqs 6–10 (black curve) and from Eq 14 (red line). The Matlab codes can be found in S23 Code.
(TIF)

S27 FigWith parameters in Table 1, improved maximal growth rates and nutrient affinities generally—but do not always—improve individual fitness and community function.In all figures, solid and dashed lines, respectively, represent calculations with $f_{P}=f_{P}^{*}=0.41$ (optimal for community function; Fig 2B) and $f_{P}=f_{P,Mono}^{*}=0.13$ (the starting point for most of our simulations; optimal for M monoculture production when Byproduct is in excess—see S25B Fig). Except for the growth parameter indicated on the horizontal axis, all other growth parameters were set to their respective upper bounds. (A–D) Community function increases as the indicated growth parameter increases. For example, in (A), all growth parameters except for *g*_*Mmax*_ were set to their upper bounds. For each *g*_*Mmax*_, the steady-state *ϕ*_*M*,*SS*_ was calculated using equations in Methods Section 1. This steady-state *ϕ*_*M*,*SS*_ was then used to calculate *P*(*T*). (F–I) The ratio between mutant population (whose indicated growth parameter was 10% lower than the upper bound) and preadapted population (with all growth parameters at upper bounds) over maturation time *T* = 17. The decreasing ratio indicates that the mutant has a lower fitness compared to the growth-adapted cells. For example, in (F), a Newborn community had 70 M and 30 H. Among M cells, 90% were preadapted and had upper bound *g*_*Mmax*_ = 0.7 ("upper bound"). The remaining 10% had *g*_*Mmax*_ = 0.63, 10% less than the upper bound ("mutant"). The ratio between "mutant" and "upper bound" cells declined over maturation time, indicating that mutant M cells had a lower fitness. (E, J) When *f*_*P*_ = 0.13 (black dashed line) but not when *f*_*P*_ = 0.41 (magenta line), increasing M’s affinity for Resource (1/*K*_*MR*_) slightly decreases individual fitness and barely affects community function. The Matlab codes can be found in S24 Code.(TIF)

S28 FigAt low *f*_*P*_, M’s lower affinity for Resource can increase its growth rate.(A) The ratio between M_LowAff_ (the population size of M with low affinity for Resource $K_{MR}^{-1}=2.5\tilde{R}(0{)}^{-1}$) and M_HighAff_ (the population size of M with high affinity for Resource $K_{MR}^{-1}=3\tilde{R}(0{)}^{-1}$) are plotted over a maturation cycle when grown together in the H-M community. The *f*_*P*_ values of both populations equaled to 0.1 (solid line), 0.2 (dotted line), and 0.3 (dashed line). (B) *P*(*T*) improves over increasing affinity $K_{MR}^{-1}$ when *f*_*P*_ is 0.1 (solid line), 0.2 (dotted line), and 0.3 (dashed line). The dependence of *P*(*T*) on affinity $K_{MR}^{-1}$ is rather weak for low *f*_*P*_. For example, when $K_{MR}^{-1}$ increases from 1 to 3, *P*(*T*) increases by only 2% and 0.6% for *f*_*P*_ = 0.2 and *f*_*P*_ = 0.1, respectively. The Matlab codes can be found in S25 Code.(TIF)

S29 FigSelection dynamics of communities of preadapted H and M when allowing all parameters to vary.In the Newborn communities of the first cycle of community selection, all growth parameters of H and M were at their upper bounds and $f_{P}=f_{P,Mono}^{*}=0.13$ (S25 Fig). The top-dog strategy was used to choose Adults that were then reproduced via pipetting. When we simulated community selection while allowing all growth parameters and *f*_*P*_ to vary, M’s affinity for R $1/{\bar{K}}_{MR}$ decreased slightly because at low *f*_*P*_ = 0.13, M with a lower affinity for R (lower 1/*K*_*MR*_) has a slightly improved individual fitness (S28 Fig). Other growth parameters (${\bar{g}}_{Mmax},{\bar{g}}_{Hmax},1/{\bar{K}}_{MB}$, and $1/{\bar{K}}_{HR}$) remain mostly constant during community selection because mutants with lower-than-maximal values were selected against by intracommunity selection and by intercommunity selection (S27 Fig). Other legends are the same as in S8 Fig. The simulation codes can be found in S4 Code, and the data can be found in S21 Data.(TIF)

S30 FigDifferent methods of pregrowth had limited impact on selection dynamics.An M monoculture grew from a single non-null M cell. This M cell went through approximately 23 doublings and therefore multiplied into approximately 10^7^ cells. Every time a non-null M cell divides, the mother and daughter cells can independently mutate and become a null M cell (*f*_*P*_ = 0) at a fixed probability of 10^−3^. If a non-null M cell has *f*_*P*_ = 0.13, then it will grow at a rate 87% of that of a null cell. After approximately 23 doublings, the M monocultures have on average about 3% null mutants. Sixty randomly chosen M cells from the same monoculture or from distinct monocultures, together with 40 H cells, were used to inoculate each of the 100 Newborns for the first selection cycle. (Top panels) Histograms of the number of Newborn communities of the first cycle that are free of noncontributor M mutants when inoculated from a single M monoculture (Left panel) or from independently grown M monocultures (right panel). To generate the histograms, the pregrowth and inoculation process was repeated 1,000 times. (Middle and bottom panels) Improvement in ${\bar{f}}_{P}(T)$ and $\bar{P}(T)$ was only slightly slower when Newborn communities from the first cycle were inoculated by the same M monoculture (left panel) than by distinct monocultures (right panel). The top-dog strategy was used to choose Adults that were then reproduced via cell sorting. Black, cyan, and gray curves are independent simulation trials. $\bar{P}(T)$ was averaged across the 2 chosen Adults. ${\bar{f}}_{P}(T)$ was obtained by first averaging among M within each chosen Adult and then averaging across the chosen Adults. The simulation codes for evolution dynamics can be found in S26 Code, the simulation codes for the histograms can be found in S27 Code, and the data can be found in S22 Data.(TIF)

S1 Code Matlab codes that generated Fig 2. (ZIP)

S2 Code Matlab codes that generated Figs 3 and 4. (ZIP)

S3 Code Matlab codes that generated Fig 5. (ZIP)

S4 Code Matlab codes that generated Fig 6. (ZIP)

S5 Code Matlab codes that generated S2 Fig. (ZIP)

S6 Code Matlab codes that generated S5 Fig. (ZIP)

S7 Code Matlab codes that generated S6 Fig. (ZIP)

S8 Code Matlab codes that generated S7 Fig. (ZIP)

S9 Code Matlab codes that generated S8 Fig. (ZIP)

S10 Code Matlab codes that generated S9 Fig. (ZIP)

S11 Code Matlab codes that generated S10 Fig. (ZIP)

S12 Code Matlab codes that generated S11 Fig. (ZIP)

S13 Code Matlab codes that generated S13 Fig. (ZIP)

S14 Code Matlab codes that generated S19A Fig. (ZIP)

S15 Code Matlab codes that generated S19B Fig. (ZIP)

S16 Code Matlab codes that generated S21 Fig. (ZIP)

S17 Code Matlab codes that generated S22A Fig. (ZIP)

S18 Code Matlab codes that generated S22B Fig. (ZIP)

S19 Code Matlab codes that generated S23 Fig. (ZIP)

S20 Code Matlab codes that generated S24 Fig. (ZIP)

S21 Code Matlab codes that generated S25A Fig. (ZIP)

S22 Code Matlab codes that generated S25B Fig. (ZIP)

S23 Code Matlab codes that generated S26 Fig. (ZIP)

S24 Code Matlab codes that generated S27 Fig. (ZIP)

S25 Code Matlab codes that generated S28 Fig. (ZIP)

S26 Code Matlab codes that generated S30 Fig. (ZIP)

S27 Code Matlab codes that generated histograms in S30 Fig. (ZIP)

S1 Data Data plotted in Figs 3 and 4. (XLSX)

S2 Data Data plotted in Fig 5. (XLSX)

S3 Data Data plotted in Fig 6. (XLSX)

S4 Data Data plotted in S2 Fig. (XLSX)

S5 Data Data plotted in S8 Fig. (XLSX)

S6 Data Data plotted in S9 Fig. (XLSX)

S7 Data Data plotted in S10 Fig. (XLSX)

S8 Data Data plotted in S12 Fig. (XLSX)

S9 Data Data plotted in S13 Fig. (XLSX)

S10 Data Data plotted in S15 Fig. (XLSX)

S11 Data Data plotted in S16 Fig. (XLSX)

S12 Data Data plotted in S18 Fig. (XLSX)

S13 Data Data plotted in S19A Fig. (XLSX)

S14 Data Data plotted in S19B Fig. (XLSX)

S15 Data Data plotted in S21A Fig. (XLSX)

S16 Data Data plotted in S21B Fig. (XLSX)

S17 Data Data plotted in S22B Fig. (XLSX)

S18 Data Data plotted in S23 Fig. (XLSX)

S19 Data Data plotted in S24 Fig. (XLSX)

S20 Data Data plotted in S25 Fig. (XLSX)

S21 Data Data plotted in S29 Fig. (XLSX)

S22 Data Data plotted in S30 Fig. (XLSX)

## Abbreviations

Adult: Adult community
B: Byproduct
CRISPR: Cas, clustered regularly interspaced short palindromic repeats and the CRISPR-associated protein
H: Helper
M: Manufacturer
Newborn: Newborn community
P: Product
PDF: probability density function
R: Resource

## References

[1] Lawley TD, Clare S, Walker AW, Stares MD, Connor TR, Raisen C, et al Targeted restoration of the intestinal microbiota with a simple, defined bacteriotherapy resolves relapsing Clostridium difficile disease in mice. PLoS Pathog. 2012;8(10):e1002995 10.1371/journal.ppat.1002995 23133377 PMC3486913

[2] Widder S, Allen RJ, Pfeiffer T, Curtis TP, Wiuf C, Sloan WT, et al. Challenges in microbial ecology: building predictive understanding of community function and dynamics. The ISME Journal. 2016;. 10.1038/ismej.2016.45 PMC5113837 27022995

[3] Lindemann SR, Bernstein HC, Song HS, Fredrickson JK, Fields MW, Shou W, et al Engineering microbial consortia for controllable outputs. The ISME Journal. 2016;10(9):2077–2084. 10.1038/ismej.2016.26 26967105 PMC4989317

[4] Zhou J, Ma Q, Yi H, Wang L, Song H, Yuan YJ. Metabolome profiling reveals metabolic cooperation between Bacillus megaterium and Ketogulonicigenium vulgare during induced swarm motility. Applied and Environmental Microbiology. 2011;77(19):7023–7030. 10.1128/AEM.05123-11 21803889 PMC3187088

[5] Wheatley RE. The consequences of volatile organic compound mediated bacterial and fungal interactions. Antonie van Leeuwenhoek. 2002;81(1–4):357–364. 10.1023/A:1020592802234 12448734

[6] Ks Kim, Lee S Ryu CM. Interspecific bacterial sensing through airborne signals modulates locomotion and drug resistance. Nature Communications. 2013;4:1809 10.1038/ncomms2789 23651997

[7] Traxler MF, Watrous JD, Alexandrov T, Dorrestein PC, Kolter R. Interspecies interactions stimulate diversification of the Streptomyces coelicolor secreted metabolome. mBio. 2013;4(4). 10.1128/mBio.00459-13 PMC3747584 23963177

[8] Gunst RF, Mason RL. Fractional factorial design. Wiley Interdisciplinary Reviews: Computational Statistics. 2009;1(2):234–244.

[9] Chen Y, Lin CJ, Jones G, Fu S, Zhan H. Enhancing biodegradation of wastewater by microbial consortia with fractional factorial design. Journal of hazardous materials. 2009;171(1–3):948–953. 10.1016/j.jhazmat.2009.06.100 19632773

[10] Eng A, Borenstein E. Microbial community design: methods, applications, and opportunities. Current opinion in biotechnology. 2019;58:117–128. 10.1016/j.copbio.2019.03.002 30952088 PMC6710113

[11] Kehe J, Kulesa A, Ortiz A, Ackerman CM, Thakku SG, Sellers D, et al Massively parallel screening of synthetic microbial communities. Proc Natl Acad Sci U S A. 2019:201900102 10.1073/pnas.1900102116 31186361 PMC6600964

[12] Swenson W, Wilson DS, Elias R. Artificial ecosystem selection. Proceedings of the National Academy of Sciences. 2000;97:9110–9114. 10.1073/pnas.150237597 PMC16830 10890915

[13] Swenson W, Arendt J, Wilson DS. Artificial selection of microbial ecosystems for 3-chloroaniline biodegradation. Environ Microbiol. 2000;2(5):564–71. 11233164 10.1046/j.1462-2920.2000.00140.x

[14] Williams HTP, Lenton TM. Artificial selection of simulated microbial ecosystems. Proceedings of the National Academy of Sciences. 2007;104(21):8918–8923. 10.1073/pnas.0610038104 PMC1885603 17517642

[15] Panke-Buisse K, Poole AC, Goodrich JK, Ley RE, Kao-Kniffin J. Selection on soil microbiomes reveals reproducible impacts on plant function. The ISME journal. 2015;9(4):980 10.1038/ismej.2014.196 25350154 PMC4817706

[16] Mueller UG, Juenger T, Kardish M, Carlson A, Burns K, Smith C, et al. Artificial Microbiome-Selection to Engineer Microbiomes That Confer Salt-Tolerance to Plants. bioRxiv. 2016; p. 081521. 10.1128/mSystems.01125-21 PMC8631316 34846165

[17] Wilson DS. Complex interactions in metacommunities, with implications for biodiversity and higher levels of selection. Ecology. 1992;73(6):1984–2000.

[18] Goodnight CJ. The influence of environmental variation on group and individual selection in a cress. Evolution. 1985;39(3):545–558. 10.1111/j.1558-5646.1985.tb00394.x 28561972

[19] Day MD, Beck D, Foster JA. Microbial communities as experimental units. Bioscience. 2011;61(5):398–406. 10.1525/bio.2011.61.5.9 21731083 PMC3128510

[20] Mueller UG, Sachs JL. Engineering Microbiomes to Improve Plant and Animal Health. Trends in Microbiology. 2015;23(10):606–617. 10.1016/j.tim.2015.07.009 26422463

[21] Crameri A, Whitehorn EA, Tate E, Stemmer WP. Improved green fluorescent protein by molecular evolution using DNA shuffling. Nature Biotechnology. 1996;14(3):315–319. 10.1038/nbt0396-315 9630892

[22] Reetz MT, Carballeira JD. Iterative saturation mutagenesis (ISM) for rapid directed evolution of functional enzymes. Nature Protocols. 2007;2(4):891–903. 10.1038/nprot.2007.72 17446890

[23] Boder ET, Midelfort KS, Wittrup KD. Directed evolution of antibody fragments with monovalent femtomolar antigen-binding affinity. Proceedings of the National Academy of Sciences. 2000;97(20):10701–10705. 10.1073/pnas.170297297 PMC27086 10984501

[24] Strauss SK, Schirman D, Jona G, Brooks AN, Kunjapur AM, Ba ANN, et al Evolthon: A community endeavor to evolve lab evolution. PLoS Biol. 2019;17(3):e3000182 10.1371/journal.pbio.3000182 30925180 PMC6440615

[25] Damore JA, Gore J. Understanding microbial cooperation. Journal of theoretical biology. 2012;299:31–41. 10.1016/j.jtbi.2011.03.008 21419783 PMC3176967

[26] Momeni B, Xie L, Shou W. Lotka-Volterra pairwise modeling fails to capture diverse pairwise microbial interactions. Elife. 2017;6. 10.7554/eLife.25051 PMC5469619 28350295

[27] Minty JJ, Singer ME, Scholz SA, Bae CH, Ahn JH, Foster CE, et al Design and characterization of synthetic fungal-bacterial consortia for direct production of isobutanol from cellulosic biomass. Proceedings of the National Academy of Sciences. 2013;110(36):14592–14597. 10.1073/pnas.1218447110 23959872 PMC3767521

[28] Zhou K, Qiao K, Edgar S, Stephanopoulos G. Distributing a metabolic pathway among a microbial consortium enhances production of natural products. Nature biotechnology. 2015;. 10.1038/nbt.3095 PMC4867547 25558867

[29] Shin HD, McClendon S, Vo T, Chen RR. Escherichia coli Binary Culture Engineered for Direct Fermentation of Hemicellulose to a Biofuel. Applied and Environmental Microbiology. 2010;76(24):8150–8159. 10.1128/AEM.00908-10 20935118 PMC3008245

[30] Mankad T, Bungay H. Model for microbial growth with more than one limiting nutrient. Journal of biotechnology. 1988;7(2):161–166.

[31] Taheri-Araghi S, Bradde S, Sauls JT, Hill NS, Levin PA, Paulsson J, et al Cell-Size Control and Homeostasis in Bacteria. Current Biology. 2015;25(3):385–391. 10.1016/j.cub.2014.12.009 25544609 PMC4323405

[32] Lenski RE, Travisano M. Dynamics of adaptation and diversification: a 10,000-generation experiment with bacterial populations. Proceedings of the National Academy of Sciences. 1994;91(15):6808–6814. 10.1073/pnas.91.15.6808 PMC44287 8041701

[33] Waite AJ, Shou W. Adaptation to a new environment allows cooperators to purge cheaters stochastically. Proceedings of the National Academy of Sciences. 2012;109(47):19079–19086. 10.1073/pnas.1210190109 PMC3511115 23091010

[34] Rainey PB, Rainey K. Evolution of cooperation and conflict in experimental bacterial populations. Nature. 2003;425(6953):72 10.1038/nature01906 12955142

[35] Ibarra RU, Edwards JS, Palsson BO. Escherichia coli K-12 undergoes adaptive evolution to achieve in silico predicted optimal growth. Nature. 2002;420(6912):186 10.1038/nature01149 12432395

[36] Sanjuán R, Moya A, Elena SF. The distribution of fitness effects caused by single-nucleotide substitutions in an RNA virus. Proceedings of the National Academy of Sciences of the United States of America. 2004;101(22):8396–8401. 10.1073/pnas.0400146101 15159545 PMC420405

[37] Sarkisyan KS, Bolotin DA, Meer MV, Usmanova DR, Mishin AS, Sharonov GV, et al Local fitness landscape of the green fluorescent protein. Nature. 2016;533(7603):397–401. 10.1038/nature17995 27193686 PMC4968632

[38] Wloch DM, Szafraniec K, Borts RH, Korona R. Direct estimate of the mutation rate and the distribution of fitness effects in the yeast Saccharomyces cerevisiae. Genetics. 2001;159(2):441–452. 11606524 10.1093/genetics/159.2.441 PMC1461830

[39] Payen C, Sunshine AB, Ong GT, Pogachar JL, Zhao W, Dunham MJ. High-throughput identification of adaptive mutations in experimentally evolved yeast populations. PLoS Genet. 2016;12(10):e1006339 10.1371/journal.pgen.1006339 27727276 PMC5065121

[40] Shou W, Ram S, Vilar JMG. Synthetic cooperation in engineered yeast populations. Proceedings of the National Academy of Sciences of the United States of America. 2007;104(6):1877–1882. 10.1073/pnas.0610575104 17267602 PMC1794266

[41] Momeni B, Brileya KA, Fields MW, Shou W. Strong inter-population cooperation leads to partner intermixing in microbial communities. eLife. 2013;2: 00230 10.7554/eLife.00230 23359860 PMC3552619

[42] Hamilton WD. The genetical evolution of social behaviour I and II. Journal of Theoretical Biology. 1964;7(1):1–52. 5875341 10.1016/0022-5193(64)90038-4

[43] Maynard Smith J. Group Selection and Kin Selection. Nature. 1964;201(4924):1145–1147. 10.1038/2011145a0

[44] Price GR. Selection and Covariance. Nature. 1970;227(5257):520–521. 10.1038/227520a0 5428476

[45] Wade MJ. A Critical Review of the Models of Group Selection. The Quarterly Review of Biology. 1978;53(2):101–114.

[46] Muir WM. Group selection for adaptation to multiple-hen cages: selection program and direct responses. Poultry Science. 1996;75(4):447–458. 10.3382/ps.0750447 8786932

[47] Queller DC, Strassmann JE. Kin Selection and Social Insects. BioScience. 1998;48(3):165–175. 10.2307/1313262

[48] Wade MJ. An experimental study of kin selection. Evolution. 1980; p. 844–855. 10.1111/j.1558-5646.1980.tb04023.x 28581146

[49] Traulsen A, Nowak MA. Evolution of cooperation by multilevel selection. Proceedings of the National Academy of Sciences. 2006;103(29):10952–10955. 10.1073/pnas.0602530103 16829575 PMC1544155

[50] Lehmann L, Keller L, West S, Roze D. Group selection and kin selection: Two concepts but one process. Proc Natl Acad Sci USA. 2007;104(16):6736–6739. 10.1073/pnas.0700662104 17416674 PMC1871855

[51] Kerr B. Theoretical and experimental approaches to the evolution of altruism and the levels of selection. Experimental Evolution: Concepts, Methods, and Applications of Selection Experiments. 2009; p. 585–630.

[52] Bachmann H, Bruggeman FJ, Molenaar D, Branco dos Santos F, Teusink B. Public goods and metabolic strategies. Current Opinion in Microbiology. 2016;31:109–115. 10.1016/j.mib.2016.03.007 27054480

[53] Hammerschmidt K, Rose CJ, Kerr B, Rainey PB. Life cycles, fitness decoupling and the evolution of multicellularity. Nature. 2014;515(7525):75–79. 10.1038/nature13884 25373677

[54] Nowak MA. Five Rules for the Evolution of Cooperation. Science. 2006;314(5805):1560–1563. 10.1126/science.1133755 17158317 PMC3279745

[55] Goodnight CJ, Stevens L. Experimental studies of group selection: what do they tell us about group selection in nature? The American Naturalist. 1997;150 Suppl 1:S59–79. 10.1086/286050 18811313

[56] Price GR. Extension of covariance selection mathematics. Annals of human genetics. 1972;35(4):485–490. 5073694 10.1111/j.1469-1809.1957.tb01874.x

[57] Sachs JL, Mueller UG, Wilcox TP, Bull JJ. The evolution of cooperation. Q Rev Biol. 2004;79(2):135–160. 15232949 10.1086/383541

[58] Chao L, Levin BR. Structured habitats and the evolution of anticompetitor toxins in bacteria. Proc Natl Acad Sci U S A. 1981;78(10):6324–6328. 10.1073/pnas.78.10.6324 7031647 PMC349031

[59] Harcombe W. Novel cooperation experimentally evolved between species. Evolution. 2010;64(7):2166–2172. 10.1111/j.1558-5646.2010.00959.x 20100214

[60] Momeni B, Waite AJ, Shou W. Spatial self-organization favors heterotypic cooperation over cheating. Elife. 2013;2:e00960 10.7554/eLife.00960 24220506 PMC3823188

[61] Hart SFM, Skelding D, Waite AJ, Burton J, Xie L, Shou W. High-throughput quantification of microbial birth and death dynamics using fluorescence microscopy. Quantitative Biology 2019; 7(1): 69–81. 10.1007/s40484-018-0160-7 PMC6785046 31598381

[62] Hillesland KL, Stahl DA. Rapid evolution of stability and productivity at the origin of a microbial mutualism. Proceedings of the National Academy of Sciences. 2010;107(5):2124–2129. 10.1073/pnas.0908456107 PMC2836651 20133857

[63] Kato S, Haruta S, Cui ZJ, Ishii M, Igarashi Y. Effective cellulose degradation by a mixed-culture system composed of a cellulolytic Clostridium and aerobic non-cellulolytic bacteria. FEMS microbiology ecology. 2004;51(1):133–142. 10.1016/j.femsec.2004.07.015 16329862

[64] Hart SFM, Mi H, Green R, Xie L, Pineda JMB, Momeni B, et al Uncovering and resolving challenges of quantitative modeling in a simplified community of interacting cells. PLoS Biol. 2019;17(2):e3000135 10.1371/journal.pbio.3000135 30794534 PMC6402699

[65] Hartwell LH, Weinert TA. Checkpoints: controls that ensure the order of cell cycle events. Science. 1989;246(4930):629–634. 10.1126/science.2683079 2683079

[66] Moazed D. Small RNAs in transcriptional gene silencing and genome defence. Nature. 2009;457(7228):413 10.1038/nature07756 19158787 PMC3246369

[67] Westra ER, Swarts DC, Staals RH, Jore MM, Brouns SJ, van der Oost J. The CRISPRs, they are a-changin’: how prokaryotes generate adaptive immunity. Annual review of genetics. 2012;46:311–339. 10.1146/annurev-genet-110711-155447 23145983

[68] Wright S. Tempo and Mode in Evolution: A Critical Review. Ecology. 1945;26(4):415–419. 10.2307/1931666

[69] Doebeli M, Ispolatov Y, Simon B. Towards a mechanistic foundation of evolutionary theory. eLife. 2017;6:e23804 10.7554/eLife.23804 28198700 PMC5333952

[70] Wolk CP, Ernst A, Elhai J. Heterocyst metabolism and development In: The molecular biology of cyanobacteria. Springer; 1994 p. 769–823.

[71] Bachmann H, Fischlechner M, Rabbers I, Barfa N, Branco dos Santos F, Molenaar D, et al Availability of public goods shapes the evolution of competing metabolic strategies. Proceedings of the National Academy of Sciences of the United States of America. 2013;110(35):14302–14307. 10.1073/pnas.1308523110 23940318 PMC3761572

[72] Chuang JS, Rivoire O, Leibler S. Simpson’s paradox in a synthetic microbial system. Science (New York, NY). 2009;323(5911):272–275. 10.1126/science.1166739 19131632

[73] Xie L, Shou W. Community function landscape and steady state species composition shape the eco-evolutionary dynamics of arti1cial community selection. BioRxiv 264697 [Preprint]. 2018 [cited 2019 Jun 20]. https://www.biorxiv.org/content/10.1101/264697v1

[74] Spudich JL, Koshland DE Jr. Non-genetic individuality: chance in the single cell. Nature. 1976;262(5568):467 10.1038/262467a0 958399

[75] Chisholm ST, Coaker G, Day B, Staskawicz BJ. Host-microbe interactions: shaping the evolution of the plant immune response. Cell. 2006;124(4):803–814. 10.1016/j.cell.2006.02.008 16497589

[76] Ley RE, Hamady M, Lozupone C, Turnbaugh PJ, Ramey RR, Bircher JS, et al Evolution of mammals and their gut microbes. Science. 2008;320(5883):1647–1651. 10.1126/science.1155725 18497261 PMC2649005

[77] Foster KR, Schluter J, Coyte KZ, Rakoff-Nahoum S. The evolution of the host microbiome as an ecosystem on a leash. Nature. 2017;548(7665):43 10.1038/nature23292 28770836 PMC5749636

[78] Otto SP, Gerstein AC. Why have sex? The population genetics of sex and recombination; 2006. 10.1042/BST0340519 16856849

[79] Gonze D, Lahti L, Raes J, Faust K. Multi-stability and the origin of microbial community types. The ISME journal. 2017;11(10):2159 10.1038/ismej.2017.60 28475180 PMC5607358

[80] De Vuyst L, Callewaert R, Crabbé K. Primary metabolite kinetics of bacteriocin biosynthesis by Lactobacillus amylovorus and evidence for stimulation of bacteriocin production under unfavourable growth conditions. Microbiology. 1996;142(4):817–827. 10.1099/00221287-142-4-817 33725789

[81] Müller MJ, Neugeboren BI, Nelson DR, Murray AW. Genetic drift opposes mutualism during spatial population expansion. Proceedings of the National Academy of Sciences. 2014;111(3):1037–1042. 10.1073/pnas.1313285111 PMC3903240 24395776

[82] Egli T. Nutrition, microbial Encyclopedia of Microbiolog, (Moselio Schaechter, Editor) Oxford: Elsevier Academic Press; 2009; p. 308–324.

[83] Hart SFM, Pineda JMB, Chen CC, Green R, Shou W. Disentangling strictly self-serving mutations from win-win mutations in a mutualistic microbial community. eLife. 2019;8:e44812 10.7554/eLife.44812 31162049 PMC6548503

[84] Zhuang K, Vemuri GN, Mahadevan R. Economics of membrane occupancy and respiro-fermentation. Molecular systems biology. 2011;7(1):500. 21694717 10.1038/msb.2011.34 PMC3159977

[85] Peris JB, Davis P, Cuevas JM, Nebot MR, Sanjuán R. Distribution of fitness effects caused by single-nucleotide substitutions in bacteriophage f1. Genetics. 2010;185(2):603–609. 10.1534/genetics.110.115162 20382832 PMC2881140

[86] Serohijos AW, Shakhnovich EI. Merging molecular mechanism and evolution: theory and computation at the interface of biophysics and evolutionary population genetics. Current opinion in structural biology. 2014;26:84–91. 10.1016/j.sbi.2014.05.005 24952216 PMC4292934

[87] Eyre-Walker A, Keightley PD. The distribution of fitness effects of new mutations. Nature reviews Genetics. 2007;8(8):610 10.1038/nrg2146 17637733

[88] Stiffler MA, Hekstra DR, Ranganathan R. Evolvability as a function of purifying selection in TEM-1 *β* -lactamase. Cell. 2015;160(5):882–892. 10.1016/j.cell.2015.01.035 25723163

[89] Drake JW. A constant rate of spontaneous mutation in DNA-based microbes. Proceedings of the National Academy of Sciences. 1991;88(16):7160–7164. 10.1073/pnas.88.16.7160 PMC52253 1831267

[90] Lang GI, Murray AW. Estimating the Per-Base-Pair Mutation Rate in the Yeast Saccharomyces cerevisiae. Genetics. 2008;178(1):67–82. 10.1534/genetics.107.071506 18202359 PMC2206112

[91] Levy SF, Blundell JR, Venkataram S, Petrov DA, Fisher DS, Sherlock G. Quantitative evolutionary dynamics using high-resolution lineage tracking. Nature. 2015;519(7542):181 10.1038/nature14279 25731169 PMC4426284

[92] Zeyl C, DeVisser JAG. Estimates of the rate and distribution of fitness effects of spontaneous mutation in Saccharomyces cerevisiae. Genetics. 2001;157(1):53–61. 11139491 10.1093/genetics/157.1.53 PMC1461475

[93] Barrick JE, Yu DS, Yoon SH, Jeong H, Oh TK, Schneider D, et al Genome evolution and adaptation in a long-term experiment with Escherichia coli. Nature. 2009;461(7268):1243 10.1038/nature08480 19838166

[94] Swings T, Van den Bergh B, Wuyts S, Oeyen E, Voordeckers K, Verstrepen KJ, et al Adaptive tuning of mutation rates allows fast response to lethal stress in Escherichia coli. eLife. 2017;6(22939). 10.7554/eLife.22939 PMC5429094 28460660

[95] Perfeito L, Fernandes L, Mota C, Gordo I. Adaptive mutations in bacteria: high rate and small effects. Science. 2007;317(5839):813–815. 10.1126/science.1142284 17690297

[96] Gillespie JH. Molecular evolution over the mutational landscape. Evolution. 1984;38(5):1116–1129. 10.1111/j.1558-5646.1984.tb00380.x 28555784

[97] Orr HA. The distribution of fitness effects among beneficial mutations. Genetics. 2003;163(4):1519–1526. 12702694 10.1093/genetics/163.4.1519 PMC1462510

[98] Imhof M, Schlötterer C. Fitness effects of advantageous mutations in evolving Escherichia coli populations. Proceedings of the National Academy of Sciences. 2001;98(3):1113–1117. 10.1073/pnas.98.3.1113 PMC14717 11158603

[99] Kassen R, Bataillon T. Distribution of fitness effects among beneficial mutations before selection in experimental populations of bacteria. Nature genetics. 2006;38(4):484 10.1038/ng1751 16550173

[100] Rokyta DR, Joyce P, Caudle SB, Wichman HA. An empirical test of the mutational landscape model of adaptation using a single-stranded DNA virus. Nature genetics. 2005;37(4):441 10.1038/ng1535 15778707

[101] Rokyta DR, Beisel CJ, Joyce P, Ferris MT, Burch CL, Wichman HA. Beneficial fitness effects are not exponential for two viruses. Journal of molecular evolution. 2008;67(4):368 10.1007/s00239-008-9153-x 18779988 PMC2600421

[102] Wiser MJ, Ribeck N, Lenski RE. Long-term dynamics of adaptation in asexual populations. Science. 2013;342(6164):1364–1367. 10.1126/science.1243357 24231808

[103] Jasnos L, Korona R. Epistatic buffering of fitness loss in yeast double deletion strains. Nature genetics. 2007;39(4):550 10.1038/ng1986 17322879

[104] Sanjuán R, Moya A, Elena SF. The contribution of epistasis to the architecture of fitness in an RNA virus. Proceedings of the National Academy of Sciences of the United States of America. 2004;101(43):15376–15379. 10.1073/pnas.0404125101 15492220 PMC524436

[105] Elena SF, Lenski RE. Test of synergistic interactions among deleterious mutations in bacteria. Nature. 1997;390(6658):395 10.1038/37108 9389477

[106] Araya CL, Fowler DM, Chen W, Muniez I, Kelly JW, Fields S. A fundamental protein property, thermodynamic stability, revealed solely from large-scale measurements of protein function. Proceedings of the National Academy of Sciences. 2012;109(42):16858–16863. 10.1073/pnas.1209751109 PMC3479514 23035249

[107] Khan AI, Dinh DM, Schneider D, Lenski RE, Cooper TF. Negative epistasis between beneficial mutations in an evolving bacterial population. Science. 2011;332(6034):1193–1196. 10.1126/science.1203801 21636772

[108] Chou HH, Chiu HC, Delaney NF, Segrè D, Marx CJ. Diminishing returns epistasis among beneficial mutations decelerates adaptation. Science. 2011;332(6034):1190–1192. 10.1126/science.1203799 21636771 PMC3244271

[109] Kryazhimskiy S, Rice DP, Jerison ER, Desai MM. Global epistasis makes adaptation predictable despite sequence-level stochasticity. Science. 2014;344(6191):1519–1522. 10.1126/science.1250939 24970088 PMC4314286

[110] Luli GW, Strohl WR. Comparison of growth, acetate production, and acetate inhibition of Escherichia coli strains in batch and fed-batch fermentations. Applied and Environmental Microbiology. 1990;56(4):1004–1011. 2187400 10.1128/aem.56.4.1004-1011.1990 PMC184335

[111] Shou W. Acknowledging selection at sub-organismal levels resolves controversy on pro-cooperation mechanisms. eLife. 2015; p. e10106 10.7554/eLife.10106 26714105 PMC4798966

[112] Lewontin RC. The Units of Selection. Annual Review of Ecology and Systematics. 1970;1(1):1–18. 10.1146/annurev.es.01.110170.000245

[113] Hill WG, Caballero A. Artificial selection experiments. Annual Review of Ecology and Systematics. 1992;23(1):287–310.

[114] Robertson A. A theory of limits in artificial selection. Proceedings of the Royal Society of London B: Biological Sciences. 1960; 153: 234–249

[115] Blouin M, Karimi B, Mathieu J, Lerch TZ. Levels and limits in artificial selection of communities. Ecology Letters. 2015; 18(10):1040–1048. . 26259498 10.1111/ele.12486

[116] Wright RJ, Gibson MI, Christie-Oleza JA. Understanding microbial community dynamics to improve optimal microbiome selection. Microbiome. 2019 7(1):85. 31159875 10.1186/s40168-019-0702-x PMC6547603

[117] Penn A. Modelling artificial ecosystem selection: A preliminary investigation. European Conference on Artificial Life 2003 (p. 659-666). Springer, Berlin, Heidelberg.

[118] Penn A, Harvey I. The Role of Non-Genetic Change in the Heritability, Variation, and Response to Selection of Artificially Selected Ecosystems. Artificial Life IX: Proceedings of the Ninth International Conference on the Simulation and Synthesis of Artificial Life 2004 (Vol. 9, p. 352). MIT Press.

[119] Goodnight CJ. Experimental studies of community evolution I: The response to selection at the community level. Evolution. 1990; 44(6):1614–1624. 28564309 10.1111/j.1558-5646.1990.tb03850.x

[120] Goodnight CJ. Experimental studies of community evolution II: The ecological basis of the response to community selection. Evolution. 1990; 44(6):1625–1636. 28564304 10.1111/j.1558-5646.1990.tb03851.x

[121] Nowak MA, Bonhoeffer S, May RM. Spatial games and the maintenance of cooperation. Proceedings of the National Academy of Sciences. 1994; 91(11):4877–81. 10.1073/pnas.91.11.4877 PMC43892 8197150

