# Supplementary figures and images for: Simulations reveal challenges to artificial community selection and possible strategies for success
Source: PLoS Biol. 2019 Jun 25;17(6):e3000295. doi: 10.1371/journal.pbio.3000295 (PMC6658139; doi:10.1371/journal.pbio.3000295)

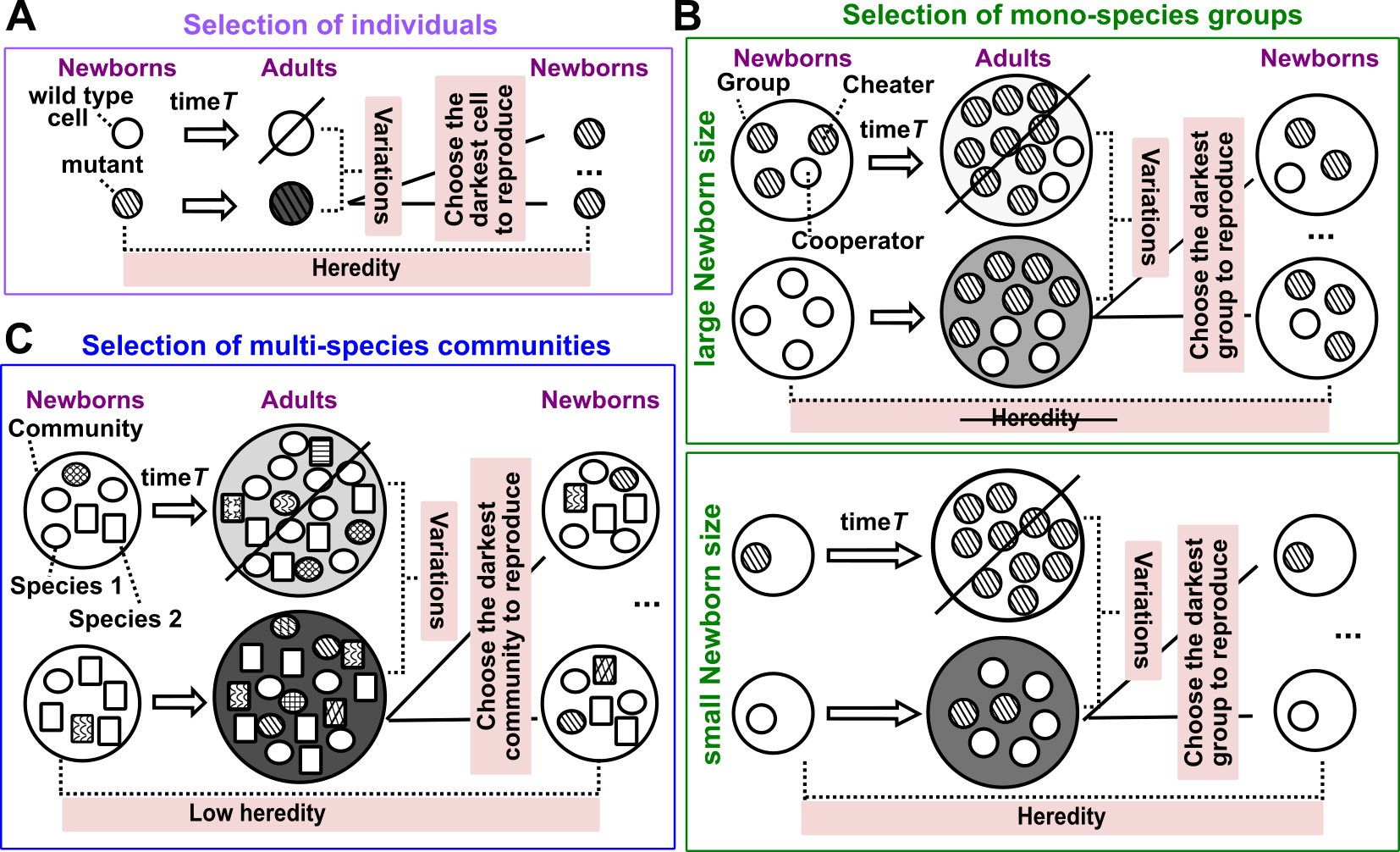

Supplement: S1 Fig — Artificial selection can be applied to any population of entities [111]. An entity can be an individual (A), a monospecies group (B), or a multispecies community (C). Unlike natural selection, which selects for fastest-growing cells, artificial selection generally selects for traits that are costly to individuals. In each selection cycle, a population of "Newborn" entities grow for maturation time T to become "Adults." Adults expressing a higher level of the trait of interest (darker shade) are chosen by the experimentalist to reproduce. An individual reproduces by making copies of itself, while an Adult group or community can reproduce by randomly splitting into multiple Newborns of the next selection cycle. Successful artificial selection requires that (i) entities display trait variations; (ii) trait variations can be selected to result in differential entity survival and reproduction; and (iii) entity trait is sufficiently heritable from one selection cycle to the next [112]. In all 3 types of selection, entity variations can be introduced by mutations and recombinations in individuals. However, heredity can be low in community selection. (A) Artificial selection of individuals has been successful [21, 22, 23, 113], since a trait is largely heritable so long as mutation and recombination are sufficiently rare. (B, C) In group selection and community selection, if maturation time T is small so that newly arising genotypes cannot rise to high frequencies within a selection cycle, then Adult trait is mostly determined by Newborn composition (the biomass of each genotype in each member species). In this case, variation can be defined as the dissimilarity in Newborn compositions within a selection cycle, while heredity can be defined as the similarity of compositions between Newborns connected through lineage across consecutive selection cycles (tubes with same-colored outlines in Fig 4A). (B) Artificial selection of monospecies groups has been successful [18, 46, 48 [file pbio.3000295.s001.tiff]

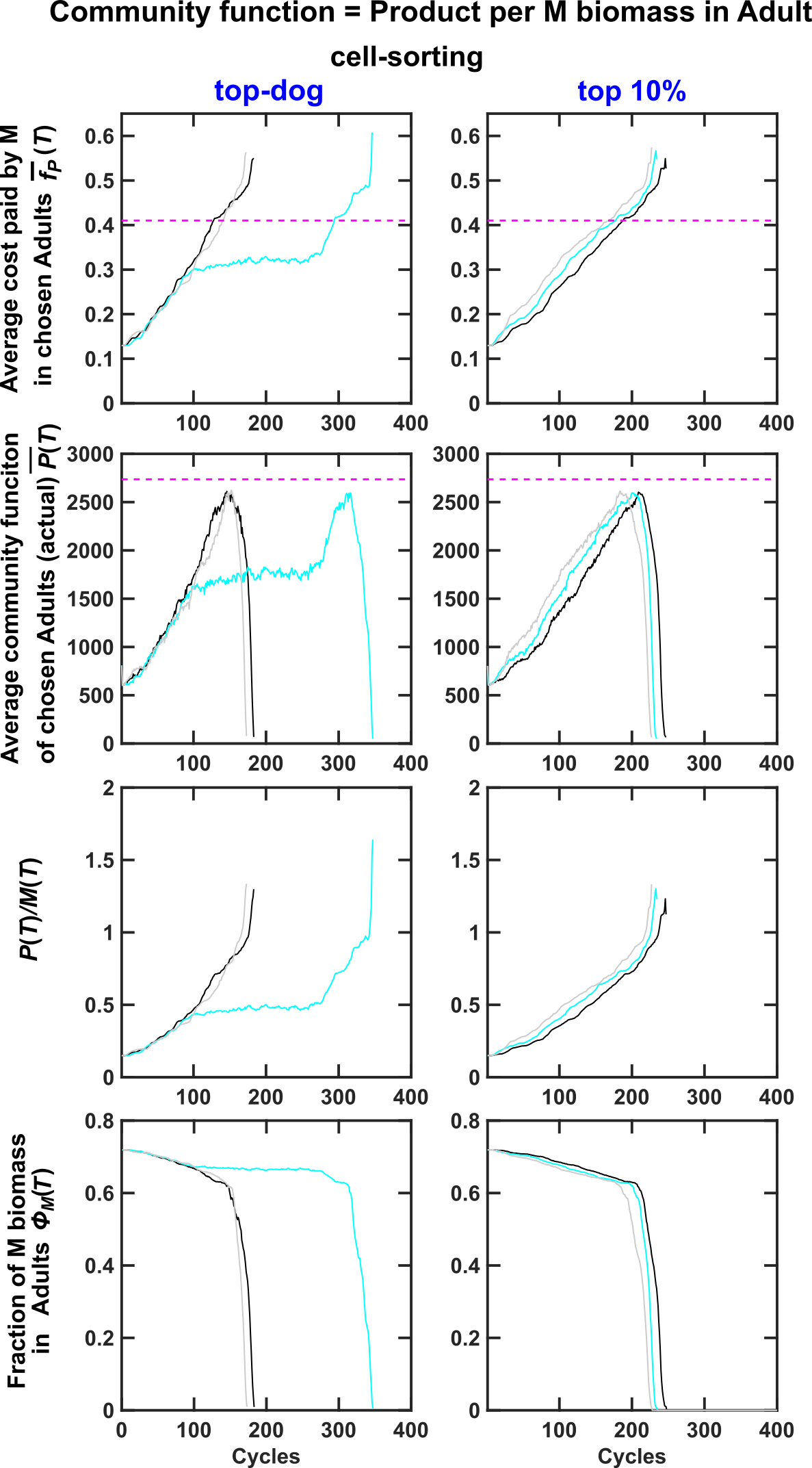

Supplement: S2 Fig — When community function was defined by P(T)/M(T), average fP of the chosen communities rapidly increased to such a high level that M was outcompeted by H, as demonstrated by Fig 2A bottom panel. Consequently, selection abruptly came to a stop. Black, cyan, and gray curves are independent simulation trials. P¯(T) was averaged across chosen Adults. f¯P(T) was obtained by first averaging among M within each chosen Adult, and then averaging across all chosen Adults. The simulation codes can be found in S5 Code, and the data can be found in S4 Data. (TIF) [file pbio.3000295.s002.tif]

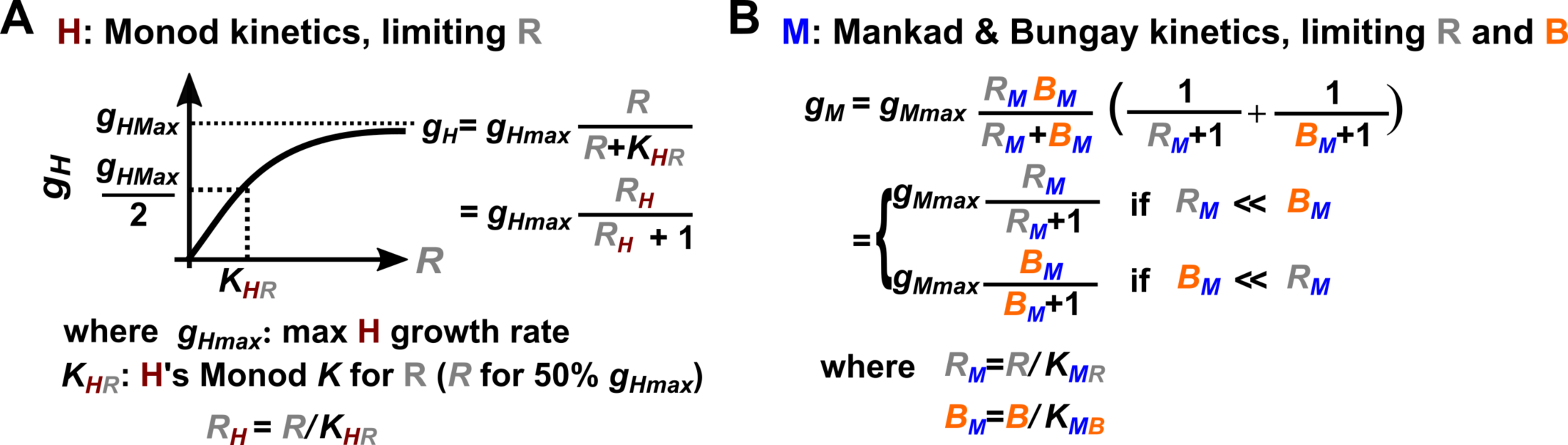

Supplement: S3 Fig — (A) H growth follows Monod kinetics, reaching half maximal growth rate when R = KHR. (B) M growth follows dual-substrate Mankad-Bungay kinetics. When Resource R is in great excess (RM ≫ BM) or Byproduct B is in great excess (BM ≫ RM), we recover monosubstrate Monod kinetics (panel A). Here, for simplicity, symbols represent absolute quantities. (TIF) [file pbio.3000295.s003.tif]

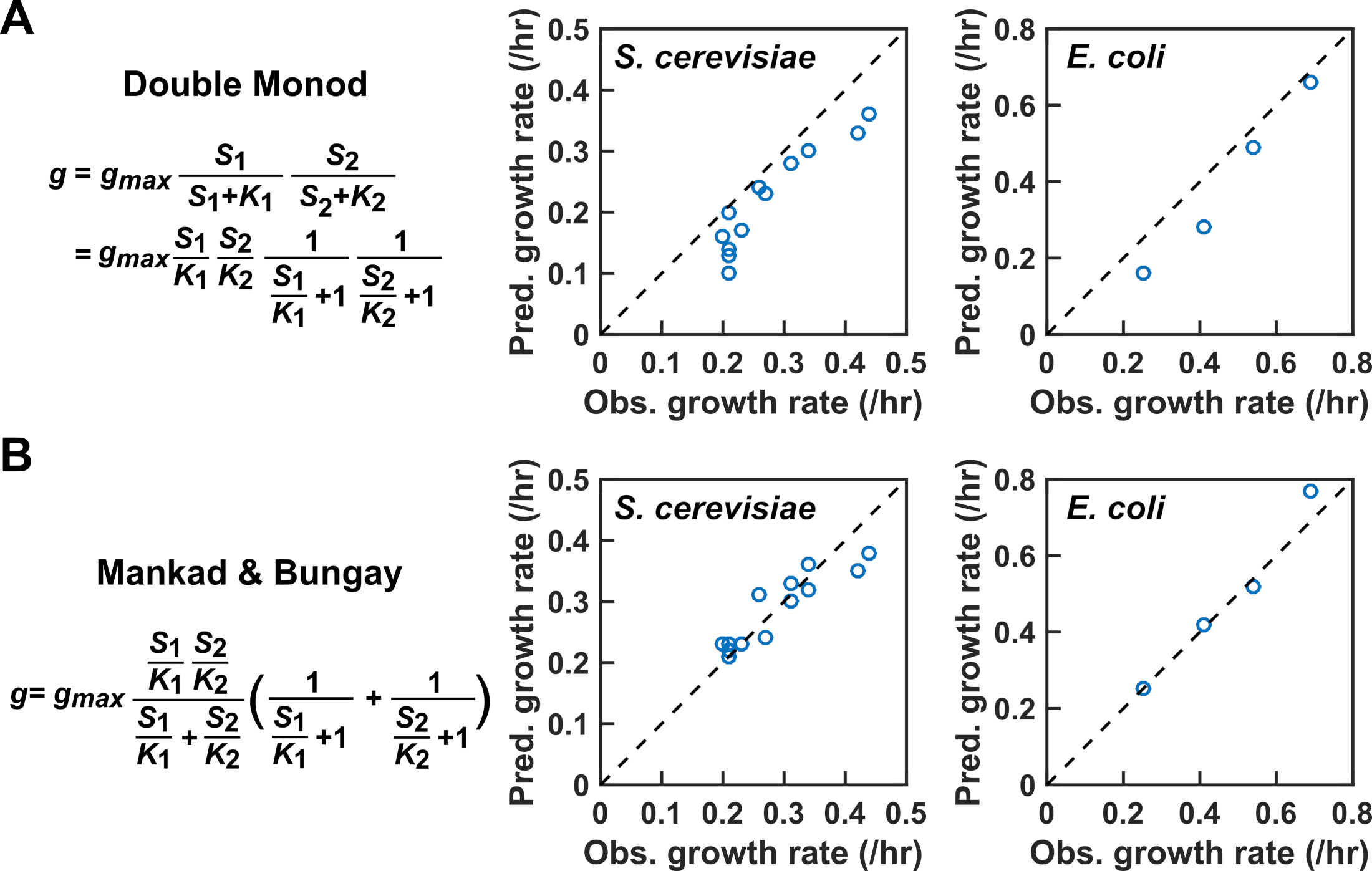

Supplement: S4 Fig — Suppose that cell growth rate depends on each substrate S1 and S2 in a Monod-like, saturable fashion. When S2 is in excess, the S1 at which half maximal growth rate is achieved is K1. When S1 is in excess, the S2 at which half maximal growth rate is achieved is K2. (A) In the "Double Monod" model, growth rate depends on the 2 limiting substrates in a multiplicative fashion. (B) In the model proposed by Mankad and Bungay, growth rate takes a different form. In both models, when one substrate is in excess, growth rate depends on the other substrate in a Monod-like fashion. However, when S1K1=S2K2=1, the growth rate is predicted to be gmax/2 by the Mankad and Bunday model, and gmax/4 by the Double Monod model. The Mankad and Bungay model outperforms the Double Monod model in describing experimental data of S. cerevisiae and E. coli growing on low glucose and low nitrogen. The figures are plotted using data from reference [30]. (TIF) [file pbio.3000295.s004.tif]

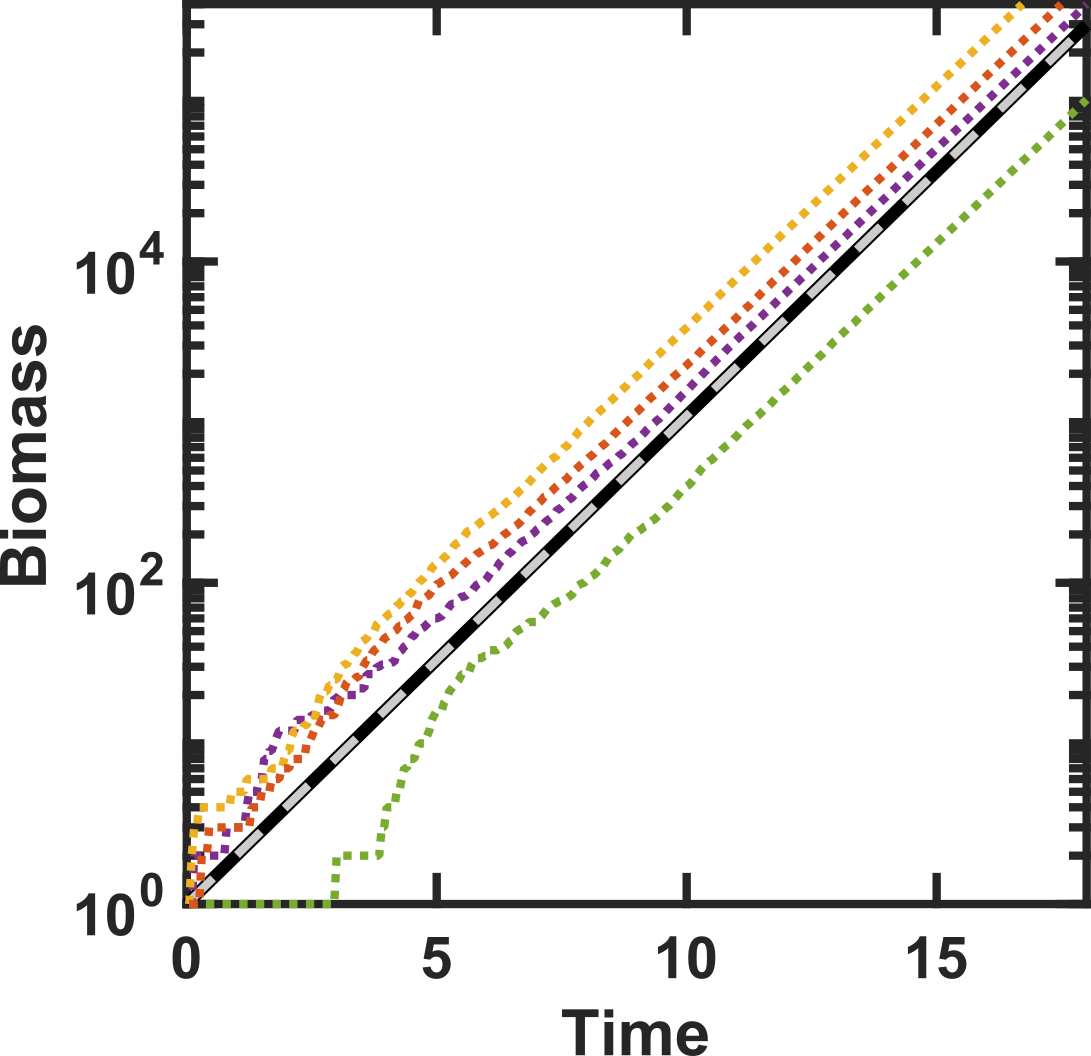

Supplement: S5 Fig — Thick black line: analytical solution with biomass growth rate (0.7/time unit). Gray dashed line: simulation assuming that biomass increases exponentially at 0.7/time unit and that cell division occurs upon reaching a biomass threshold, an assumption used in our model. Colored dotted lines: simulations assuming that cell birth is discrete and occurs at a probability equal to the birth rate multiplied with the length of simulation time step (Δτ = 0.05 time unit). When a cell birth occurs, biomass increases discretely by 1, resulting in step-wise increase in colored dotted lines at early time. The Matlab codes can be found in S6 Code. (TIF) [file pbio.3000295.s005.tif]

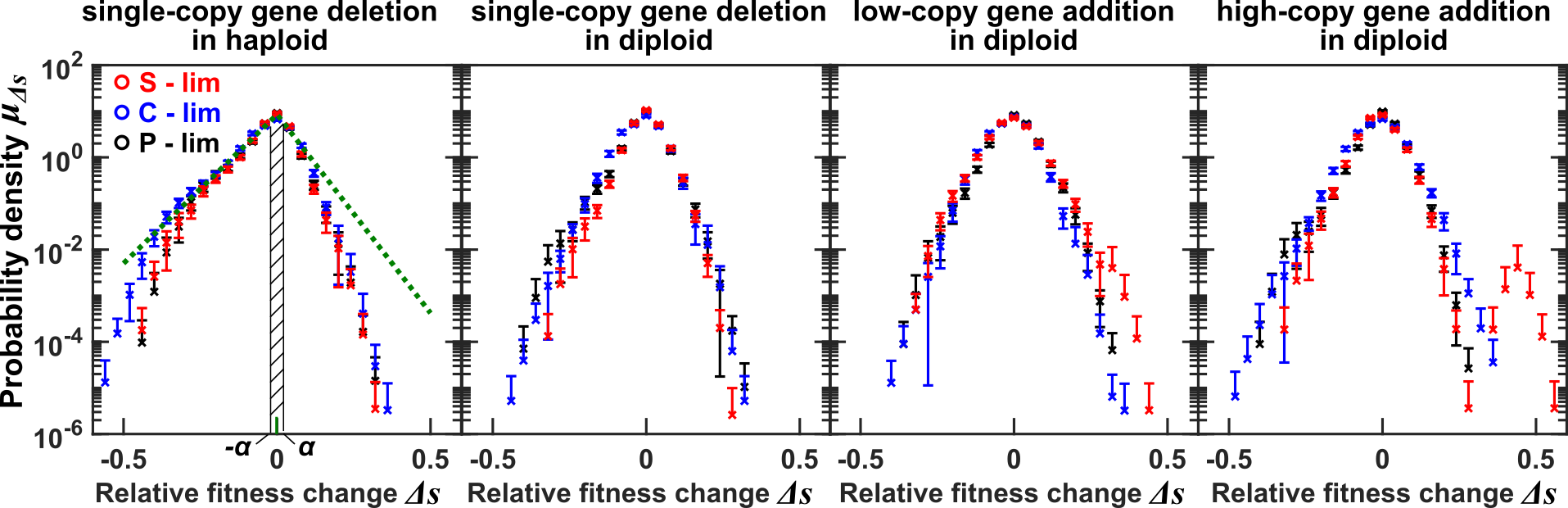

Supplement: S6 Fig — We derived μΔs(Δs) from the Dunham lab data [39] where bar-coded mutant strains were competed under sulfate limitation (red), carbon limitation (blue), or phosphate limitation (black). Error bars represent uncertainty δμΔs (the lower error bar is omitted if the lower estimate is negative). In the leftmost panel, green lines show nonlinear least squared fitting of data to Eq 19 using all 3 sets of data. Note that data with larger uncertainty are given less weight and thus deviate more from the fitting. For an exponentially distributed PDF p(x) = exp(−x/r)/r where x, r > 0, and the average of x is r. When plotted on a semi-log scale, we get a straight line with slope −1/r, which gets us the average effect r. From the green line on the right side, we obtain the average effect of enhancing mutations s+ = 0.050 ± 0.002, and from the green line on the left side, we obtain the average effect of diminishing mutations s− = 0.067 ± 0.003. The probability of a mutation altering a phenotype by ±α is the area of the hatched region drawn in the leftmost panel. The Matlab codes can be found in S7 Code. PDF, probability density function. (TIF) [file pbio.3000295.s006.tif]

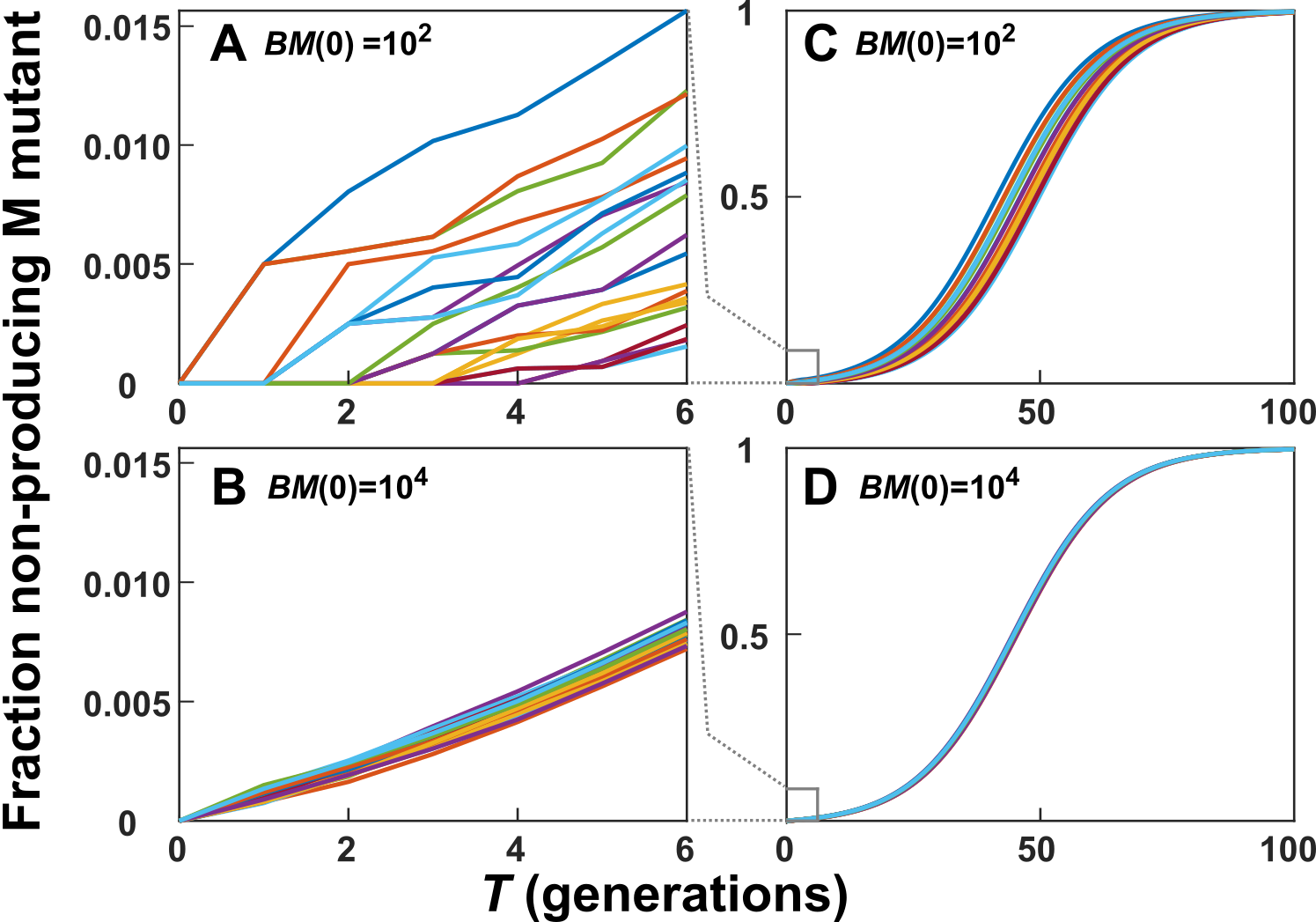

Supplement: S7 Fig — For simplicity, we modeled the growth of Newborn groups of M cells. Both wild-type and mutant cells followed exponential growth. The growth rate of wild-type cells was 0.87 times that of mutants. From a Newborn biomass BM(0) of 102 (top panels) or 104 (bottom panels) wild-type M cells, M population multiplied for 6 (left panels) or 100 (right panels) generations. Immediately following cell division, wild-type daughter cells mutated to noncontributors with a probability of 10−3. The fraction of biomass made up by mutants at each wild-type doubling is shown. The simulation codes can be found in S8 Code. (TIF) [file pbio.3000295.s007.tif]

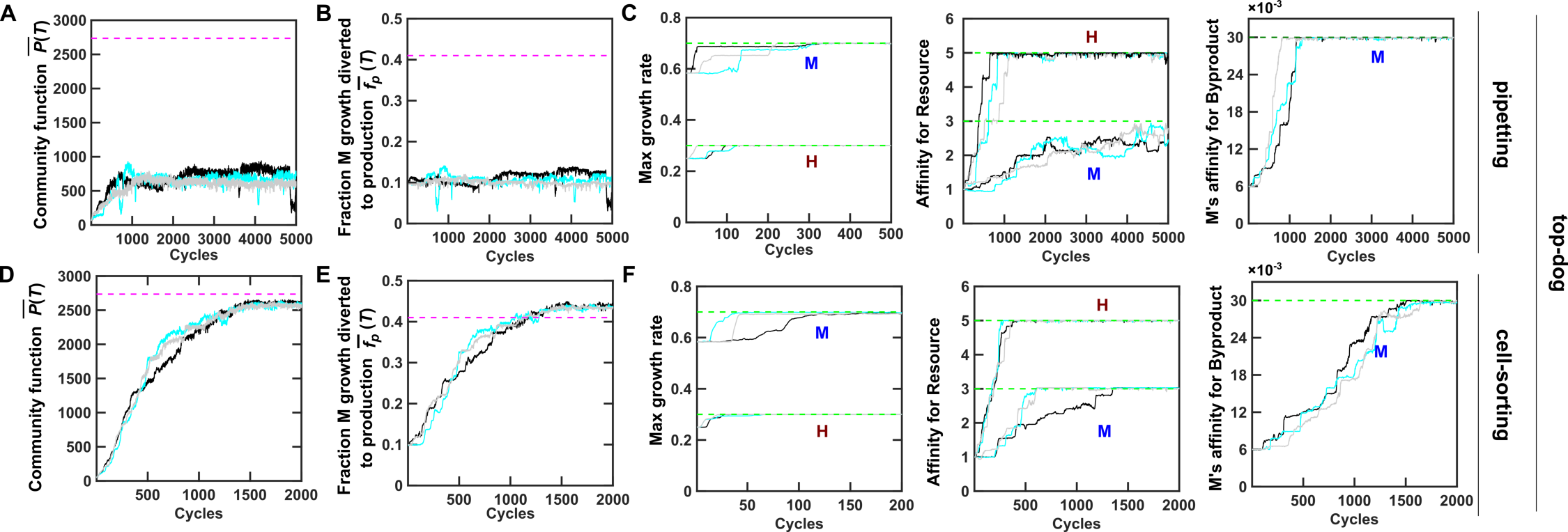

Supplement: S8 Fig — Here, we allowed mutations to alter M’s fP and H and M’s growth parameters (Table 1). Communities were chosen using the top-dog strategy. (A–C) Community reproduction via pipetting (i.e., Newborn biomass and species composition can fluctuate). Community function P(T) increased upon community selection (A). Since fP remained unchanged (panel B), this increase in P(T) must be due to improved growth parameters (panel C). (D–F) Community reproduction via biomass sorting (i.e., fixed Newborn total biomass and species composition). Community function improved to a much higher level (panel D). In both strategies, the 5 growth parameters increased to their respective evolutionary upper bounds (green dashed lines). Magenta dashed lines: optimal fP for community function and maximal community function P(T) when all 5 growth parameters are fixed at their evolutionary upper bounds and ϕM(0) is also optimal for P(T). Black, cyan, and gray curves show independent simulations. P¯(T) is averaged across chosen Adults. g¯Mmax,g¯Hmax, and f¯P are obtained by averaging within each chosen Adult and then averaging across chosen Adults. KSpeciesMetabolite are averaged within each chosen Adult, then averaged across chosen Adults, and finally inverted to represent average affinity. Note different horizontal axis scales. The maximal growth rates (gMmax and gHmax) have the unit of 1/time. Affinity for Resource (1/KMR, 1/KHR) has the unit of 1/R~(0), where R~(0) is the initial amount of Resource in Newborn. Affinity for Byproduct (1/KMB) has the unit of 1/r~B, where r~B is the amount of Byproduct released per H biomass produced. Product P has the unit of r~P, the amount of Product released at the cost of 1 M biomass. More details on parameters and variables can be found in Tables 1 and 2. The simulation codes can be found in S9 Code, and the data can be found in S5 Data. (TIF) [file pbio.3000295.s008.tif]

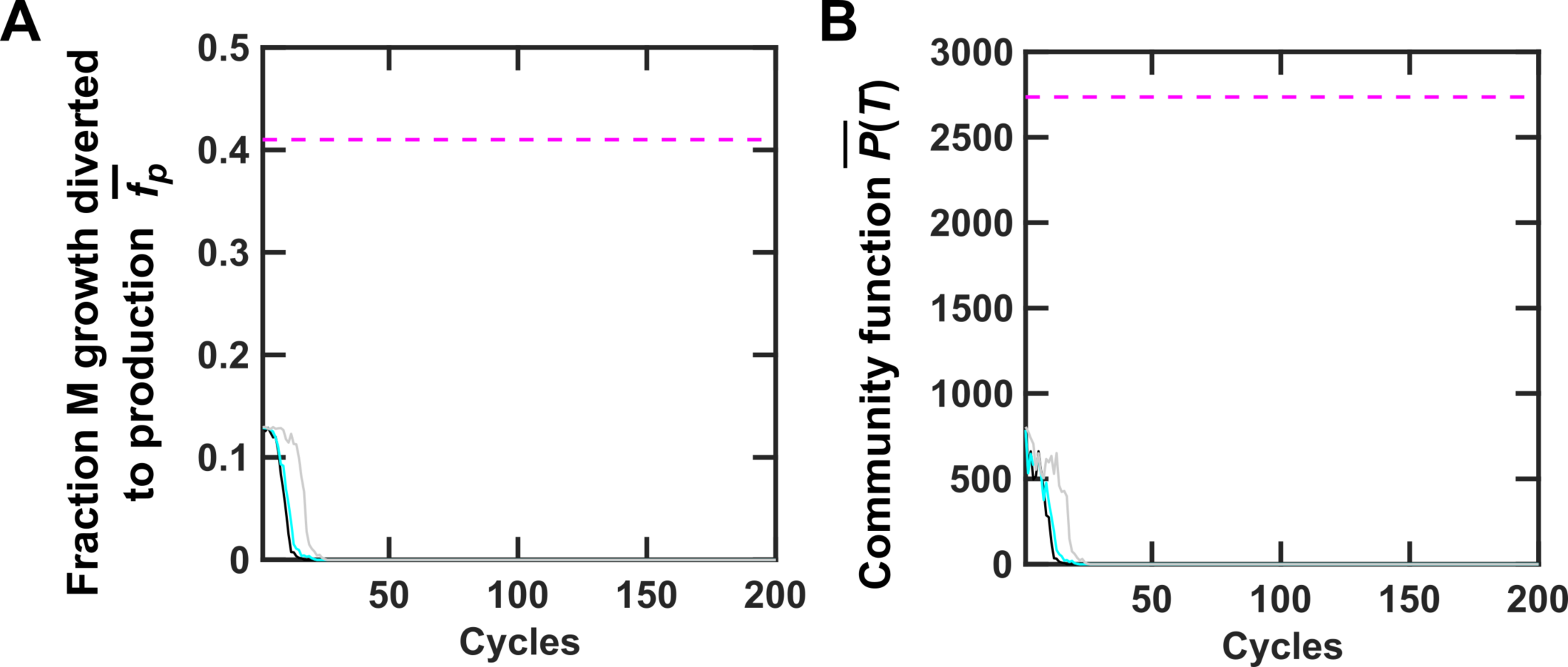

Supplement: S9 Fig — An Adult was randomly chosen to reproduce as many Newborns as possible, and a second Adult was randomly chosen to reproduce more Newborns until 100 Newborns were obtained. Natural selection favored zero fP (panel A). Consequently, P(T) decreased to zero (panel B). Here, reproduction was done by pipetting. Black, cyan, and gray curves are independent simulation trials. P¯(T) was averaged across the randomly chosen Adults. f¯P(T) was obtained by first averaging among M within each randomly chosen Adult and then averaging across the chosen Adults. The simulation codes can be found in S10 Code, and the data can be found in S6 Data. (TIF) [file pbio.3000295.s009.tif]

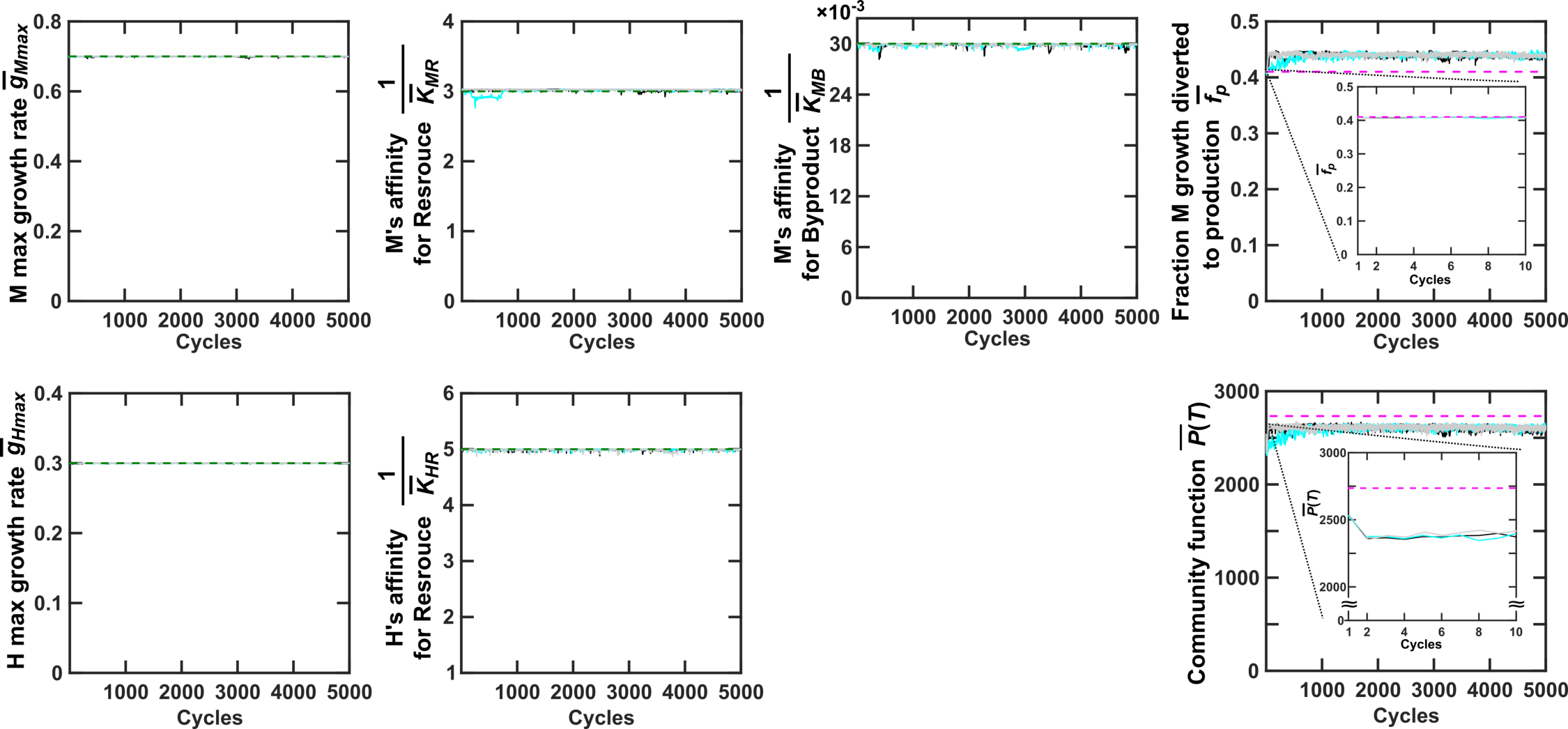

Supplement: S10 Fig — We started each Newborn community with total biomass BM(0) = 100, all 5 growth parameters at their evolutionary upper bounds, and fP*=0.41 and ϕM*(0)=0.54 to achieve P*(T). We then allowed all 5 growth parameters and fP to mutate while applying community selection. To ensure effective community selection (Fig 3D–3F), the strategy of top-dog with cell sorting was implemented. We found that all 5 growth parameters remained at their respective evolutionary upper bounds. At the end of the first cycle (Cycle = 1 in insets), even though f¯P did not change, P¯(T) had already declined from the original magenta dashed line. This is because species interactions have driven ϕM(0) from the optimal ϕM*(0) (= 0.54) to near the steady-state value (ϕM = 0.64, compare with ϕM,SS represented by the blue dashed line in Fig 2A top panel). Later, over hundreds of cycles, f¯P gradually increased, while P¯(T) was still below maximal. This is because species composition gravitated toward steady-state ϕM,SS, which deviated from the optimal ϕM*(0). Other legends are the same as S8 Fig. The simulation codes can be found in S11 Code, and the data can be found in S7 Data. (TIF) [file pbio.3000295.s010.tif]

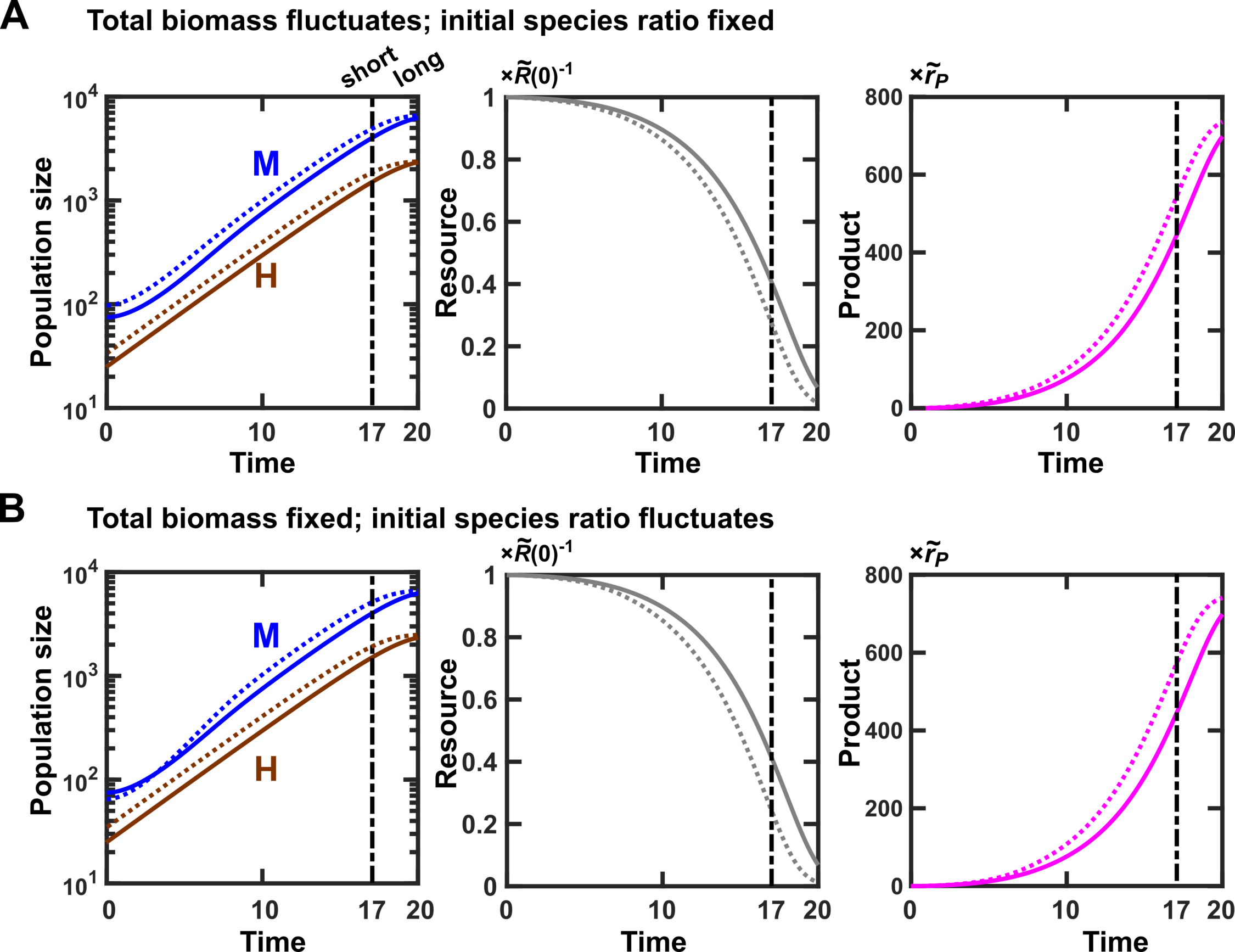

Supplement: S11 Fig — An average Newborn community (solid lines) has a total biomass of 100 with 75% M. (A) A "lucky" Newborn community (dotted lines), by stochastic fluctuations, has a higher total biomass of 130 and the average 75% M. Even though the lucky and the average communities share identical fP = 0.1, biomass of M in the lucky Newborn can grow to a higher value (left), deplete more Resource (middle), and make more Product (right) by the end of short T (T = 17). (B) A "lucky" Newborn community (dotted lines), by stochastic fluctuations, has 65% (instead of 75%) M and the average total biomass of 100. Even though the lucky and the average communities share identical fP = 0.1, higher fraction of Helper H biomass in the lucky community results in faster accumulation of Byproduct. Consequently, M can enjoy a shorter growth lag, grow to a larger size (left), deplete more Resource (middle), and make more Product (right) by the end of short T (T = 17). In both cases, the difference between the lucky (dotted) and the average (solid) communities diminished at longer T (T = 20) compared to shorter T (T = 17, dash dot line). The Matlab codes can be found in S12 Code. (TIF) [file pbio.3000295.s011.tif]

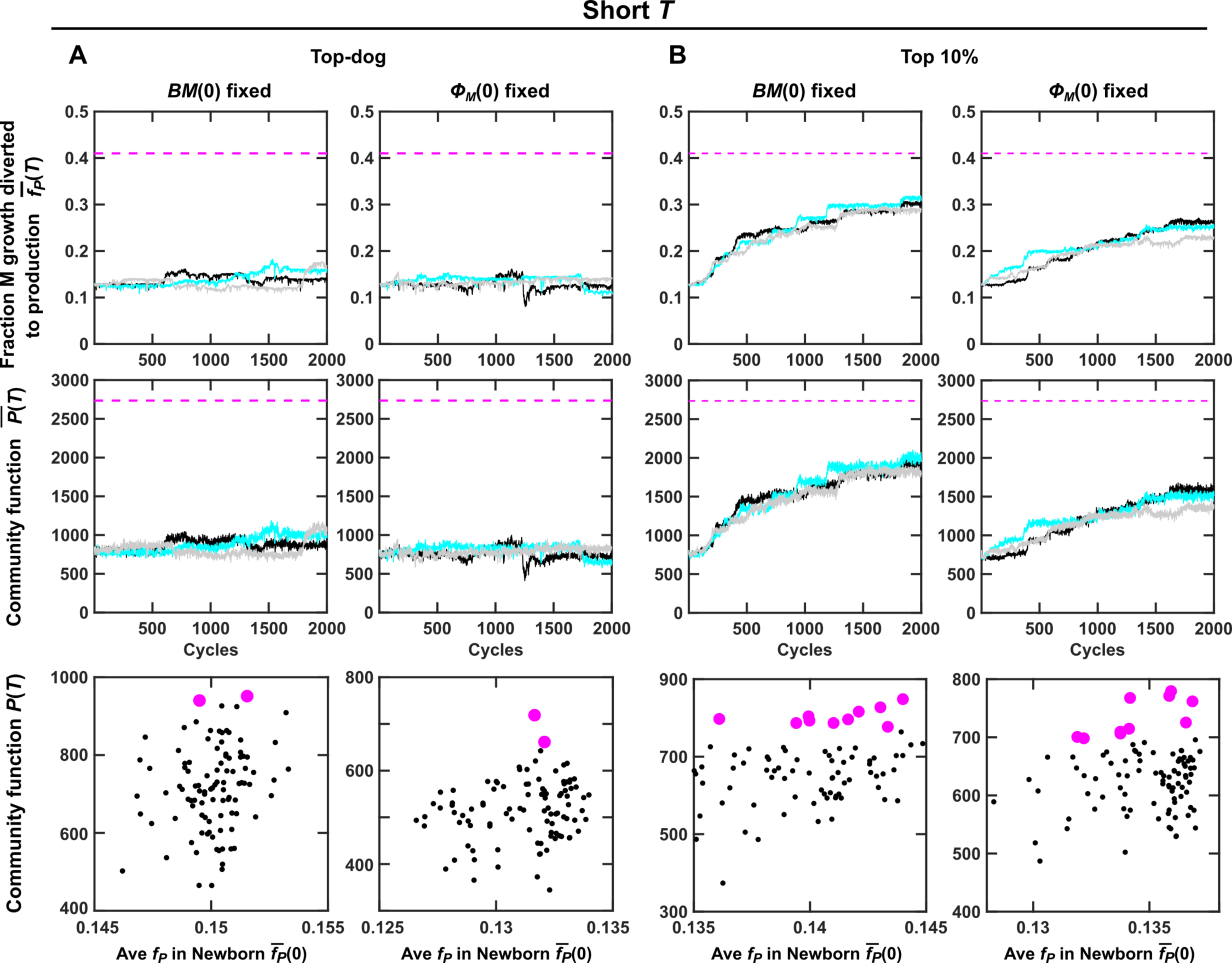

Supplement: S12 Fig — When using the top-dog strategy (A) or the top 10% strategy (B), fixing only Newborn total biomass (BM(0)) or fixing only Newborn species composition (ϕM(0)) yielded similar dynamics as fixing neither (pipetting; Fig 3A–3C and Fig 3G–3I, respectively). Black, cyan, and gray curves are 3 independent simulation trials. P¯(T) was averaged across all chosen Adults. f¯P(T) was obtained by first averaging among M within each chosen Adult and then averaging across all chosen Adults. The simulation codes can be found in S2 Code, and the data can be found in S8 Data. (TIF) [file pbio.3000295.s012.tif]

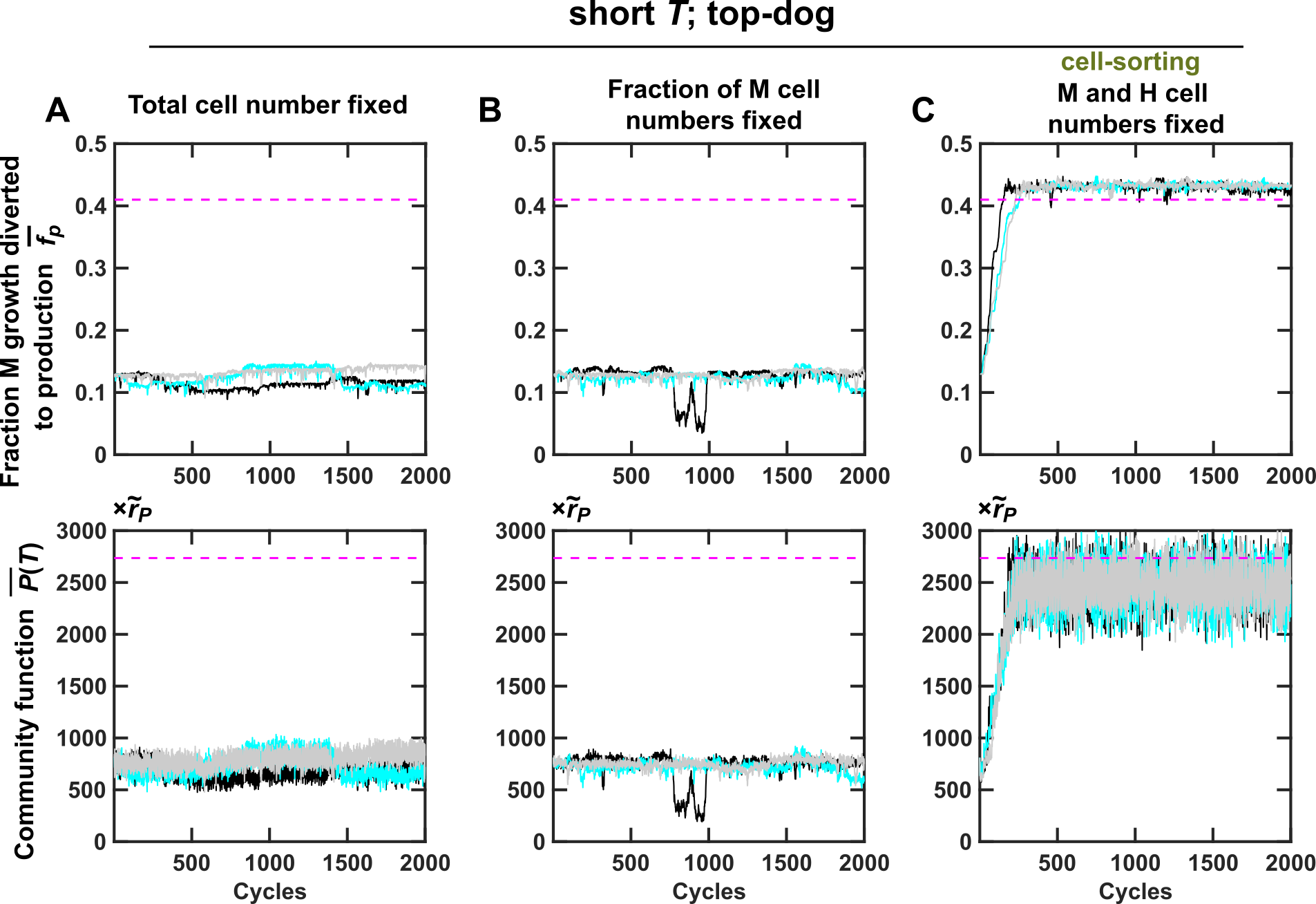

Supplement: S13 Fig — (A) The total cell number in Newborn communities was fixed to ⌊BMtarget/1.5⌋ where ⌊x⌋ means rounding down x to the nearest integer. (B) The ratio between M and H cell numbers in Newborn communities were fixed to IM(T)/IH(T), where IM(T) and IH(T) were the number of M and H cells in the chosen Adult community from the previous cycle, respectively. (C) The total cell numbers of Newborn communities were fixed to ⌊BMtarget/1.5⌋, and the ratio between M and H cell numbers were fixed to IM(T)/IH(T). See Methods Section 6 for details of simulating community reproduction. Black, cyan, and gray curves are independent simulation trials. P¯(T) was averaged across the 2 chosen Adults. f¯P(T) was obtained by first averaging among M within each chosen Adult and then averaging across the 2 chosen Adults. The simulation codes can be found in S13 Code, and the data can be found in S9 Data. (TIF) [file pbio.3000295.s013.tif]

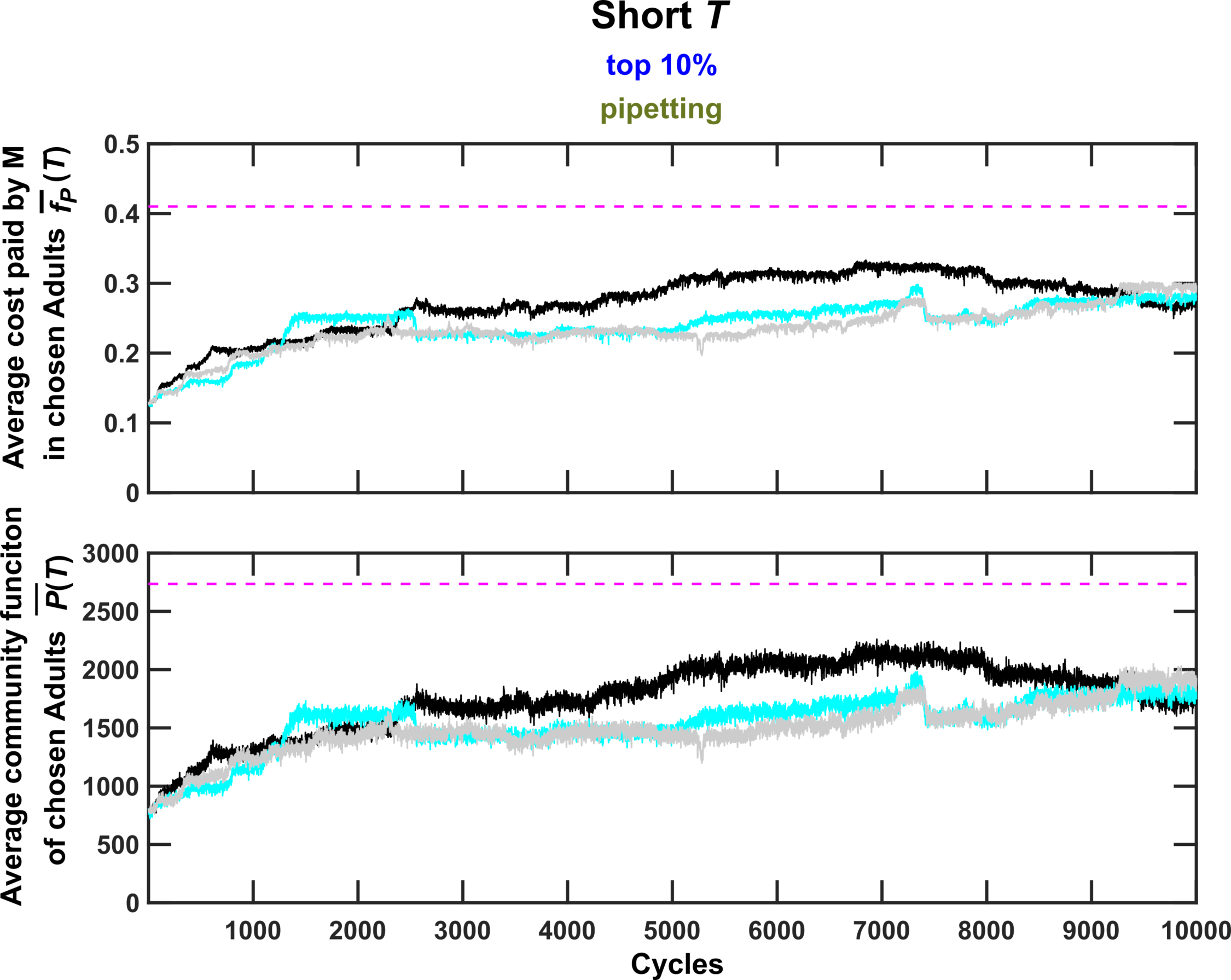

Supplement: S14 Fig — Simulation setup was identical to that in Fig 3G–3H, except that selection here lasted more cycles. Compared to Fig 3A–3C (top-dog, pipetting), the top 10% strategy was more effective. However, compared to Fig 3D–3F (top-dog, cell sorting), the top 10% strategy was less effective, even over 104 cycles. Black, cyan, and gray curves are independent simulation trials. P¯(T) was averaged across the chosen Adults. f¯P(T) was obtained by first averaging among M within each chosen Adult and then averaging across all chosen Adults. The simulation codes can be found in S2 Code, and the data can be found in S1 Data. (TIF) [file pbio.3000295.s014.tif]

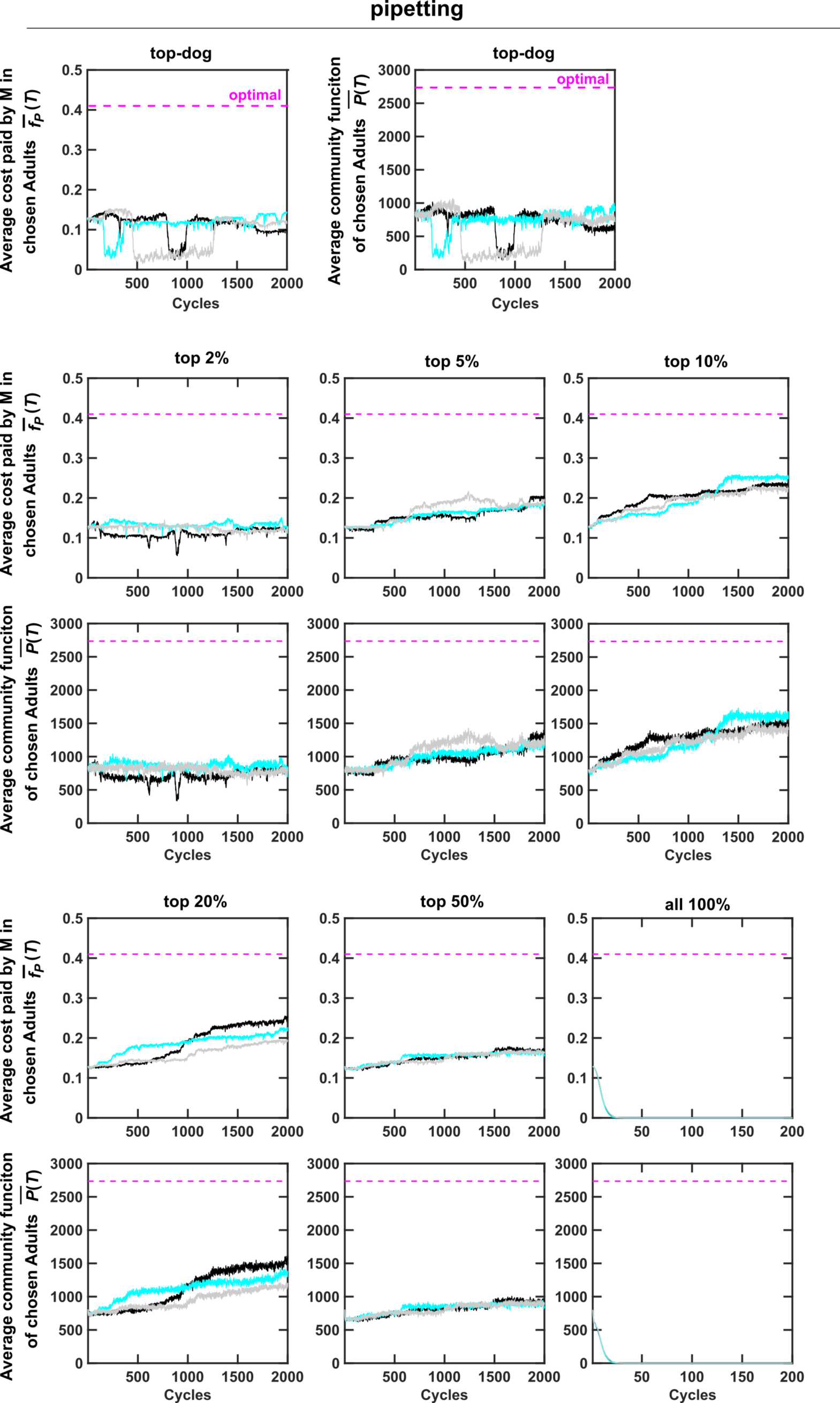

Supplement: S15 Fig — In a top-tier strategy (“top 2%” to “top 50%”), top nchosen(= 2~50) Adults each contributed 100/nchosen Newborns into the next cycle. Here, Adults were reproduced (split) into Newborns as if via pipetting. Note that “top 2%” yielded qualitatively similar results as “top-dog”. Note also that when all Adults contributed one Newborn each (“all 100%”), intercommunity selection strength was zero, and thus natural selection quickly reduced average cost fP and community function to zero. Black, cyan, and gray curves are independent simulation trials. P¯(T) was averaged across the chosen Adults. f¯P(T) was obtained by first averaging among M within each chosen Adult and then averaging across all chosen Adults. The simulation codes can be found in S2 Code, and the data can be found in S10 Data. (TIF) [file pbio.3000295.s015.tif]

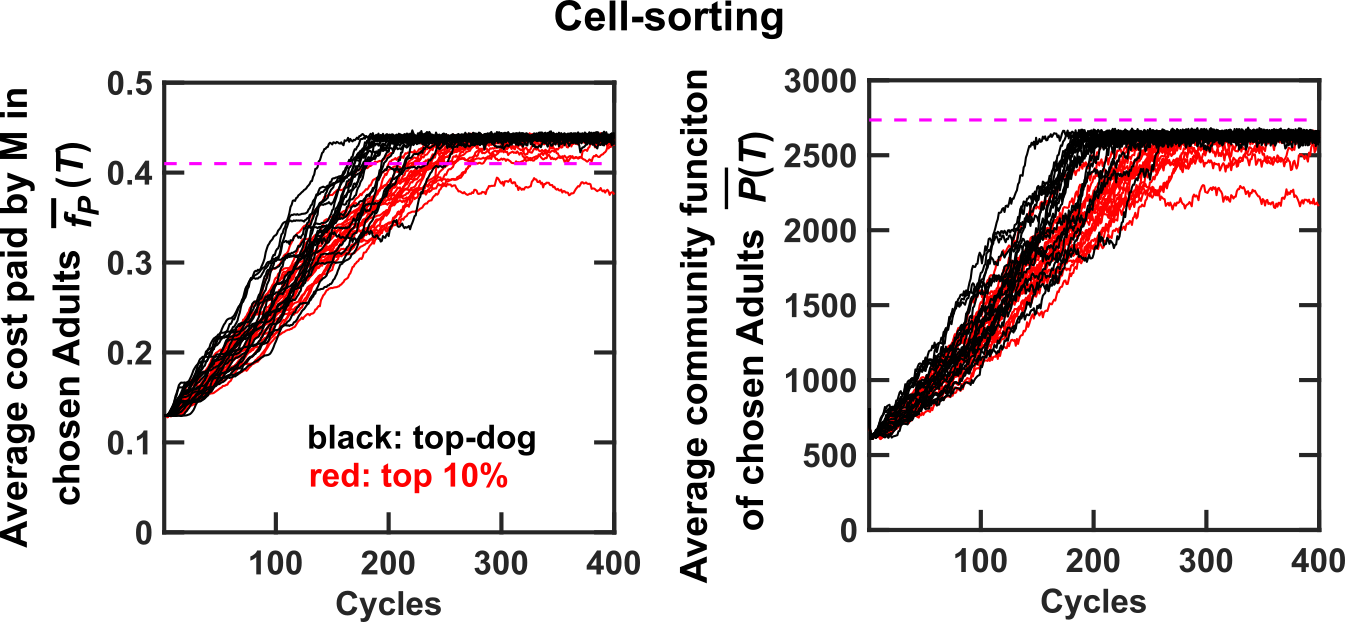

Supplement: S16 Fig — Twenty replicas of selection simulations were performed using either the top-dog strategy (black curves) or the top-tier strategy (top 10 Adults chosen to reproduce; red curves). Community reproduction was through cell sorting. Community functions improved slightly faster and to a slightly higher level using the top-dog strategy. Thus, when nonheritable variations in community function were suppressed, the top-dog strategy was superior to the top-tier strategy. The simulation codes can be found in S2 Code, and the data can be found in S11 Data. (TIF) [file pbio.3000295.s016.tif]

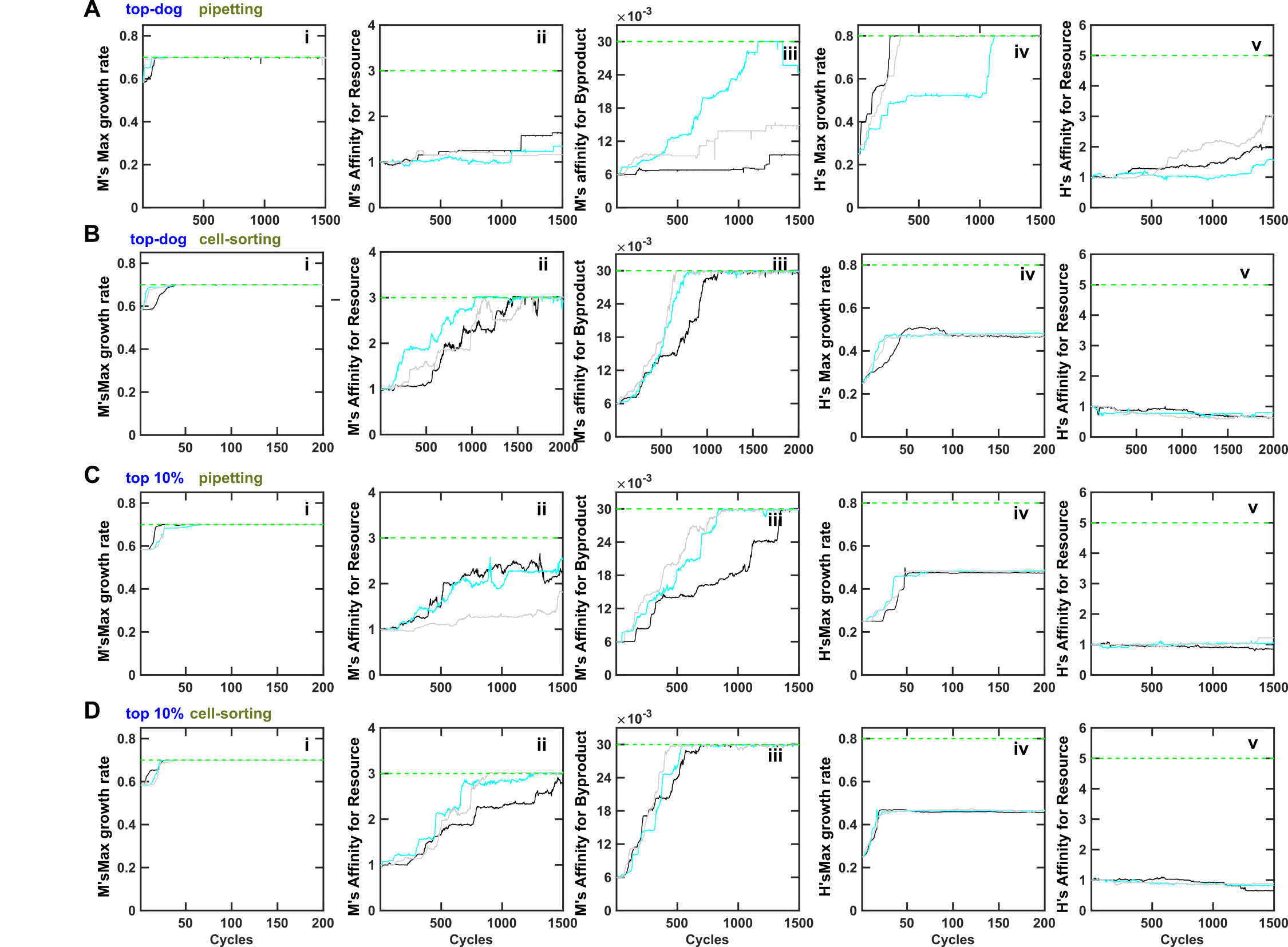

Supplement: S17 Fig — Identical to Fig 6, the evolutionary upper bound for gHmax (gHmax*=0.8) was larger than that of gMmax (gMmax*=0.7), opposite to that in Fig 3. (A) When using the top-dog strategy with pipetting, gHmax and gMmax evolved to their respective upper bounds, and thus gHmax > gHmax (compare i and iv). This would ordinarily lead to extinction of M. However, community selection managed to maintain M at a very low level (Fig 6A bottom panel). (B–D) When using the top-dog strategy with cell sorting (panel B), the top 10% strategy with pipetting (panel C), or the top 10% strategy with cell sorting (panel D), community selection worked in the sense that both f¯P and P(T) improved over cycles (Fig 6B–6D). The maximal growth rate of H gHmax did not increase to its upper bound gHmax*=0.8, and H’s affinity for Resource even decreased from the ancestral level in some cases. Here, Resource supplied to Newborn communities could support 105 total biomass to accommodate faster growth rate. Other legends are the same as S8 Fig. The simulation codes can be found in S4 Code, and the data can be found in S3 Data. (TIF) [file pbio.3000295.s017.tif]

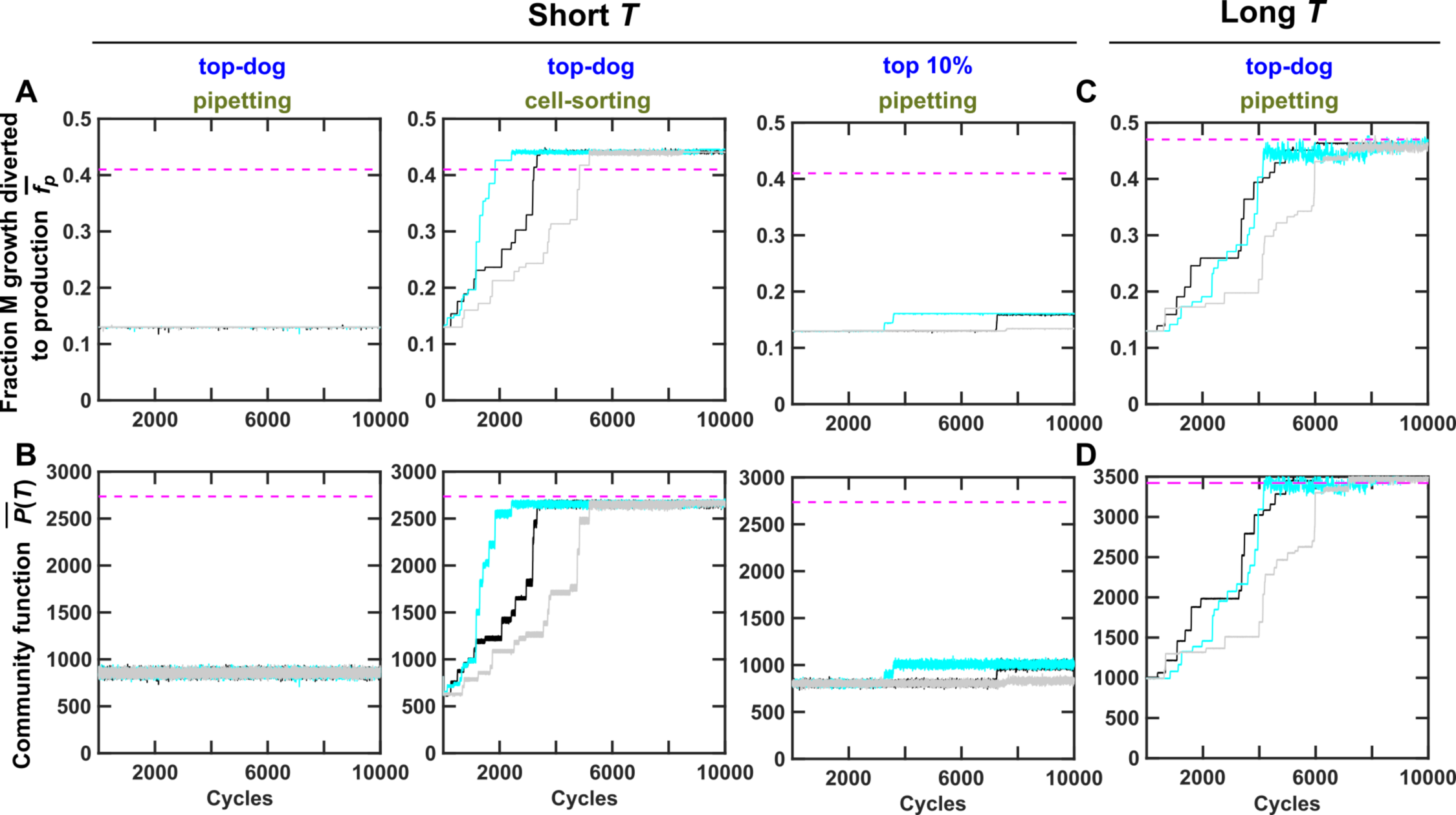

Supplement: S18 Fig — (A, B) At short maturation time (T = 17, Resource was not exhausted in an average community), cell sorting improved community function. The top-tier strategy with pipetting slightly improved community function. (C, D) At long maturation time (T = 20, Resource was nearly exhausted in an average community), community function improved without fixing BM(0) or ϕM(0) (top-dog with pipetting). At this mutation rate, because the population size of a community never exceeds 104, a mutation occurs on average every 5 cycles, resulting in step-wise improvement in both f¯P(T) and P¯(T). Black, cyan, and gray curves are independent simulation trials. P¯(T) was averaged across all chosen Adults. f¯P(T) was obtained by first averaging among M within each chosen Adult and then averaging across all chosen Adults. The simulation codes can be found in S2 Code, and the data can be found in S12 Data. (TIF) [file pbio.3000295.s018.tif]

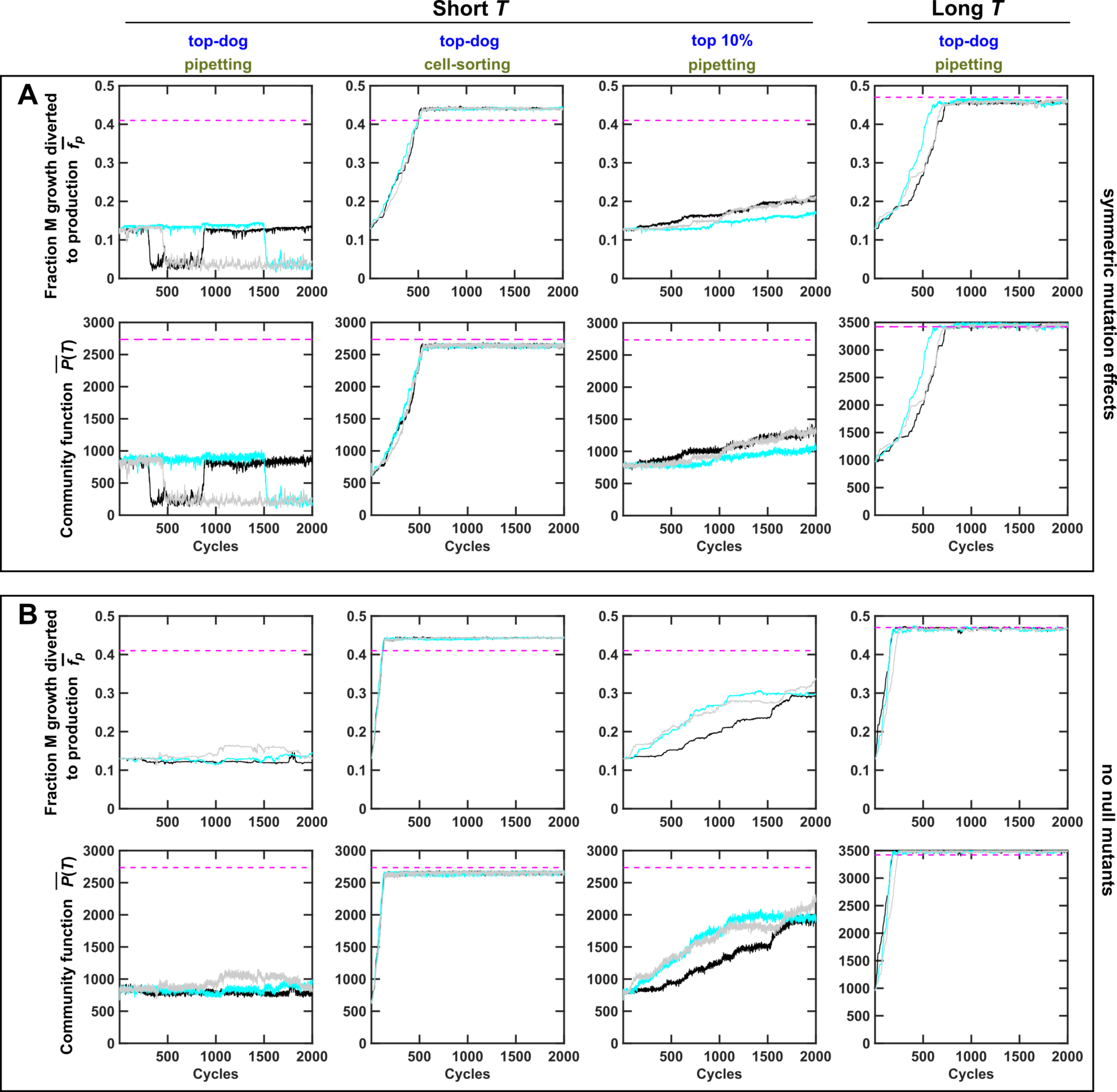

Supplement: S19 Fig — (A) Evolutionary dynamics where half of the mutations reduced fP to zero, and the distribution of mutation effects of the other half is specified by Eq 19 in which s+ = s− = 0.02 are constants. (B) Evolutionary dynamics when null mutations in fP did not occur. The distribution of mutation effects is specified by Eq 19 where s+ = 0.05 and s− = 0.067. f¯P(T) as well as P¯(T) were more stable compared to when null mutations were present (Fig 3). Black, cyan, and gray curves are independent simulation trials. P¯(T) was averaged across the chosen Adults. f¯P(T) was obtained by first averaging among M within each chosen Adult and then averaging across all chosen Adults. For panel A, the simulation codes can be found in S14 Code, and the data can be found in S13 Data. For panel B, the simulation codes can be found in S15 Code, and the data can be found in S14 Data. (TIF) [file pbio.3000295.s019.tif]

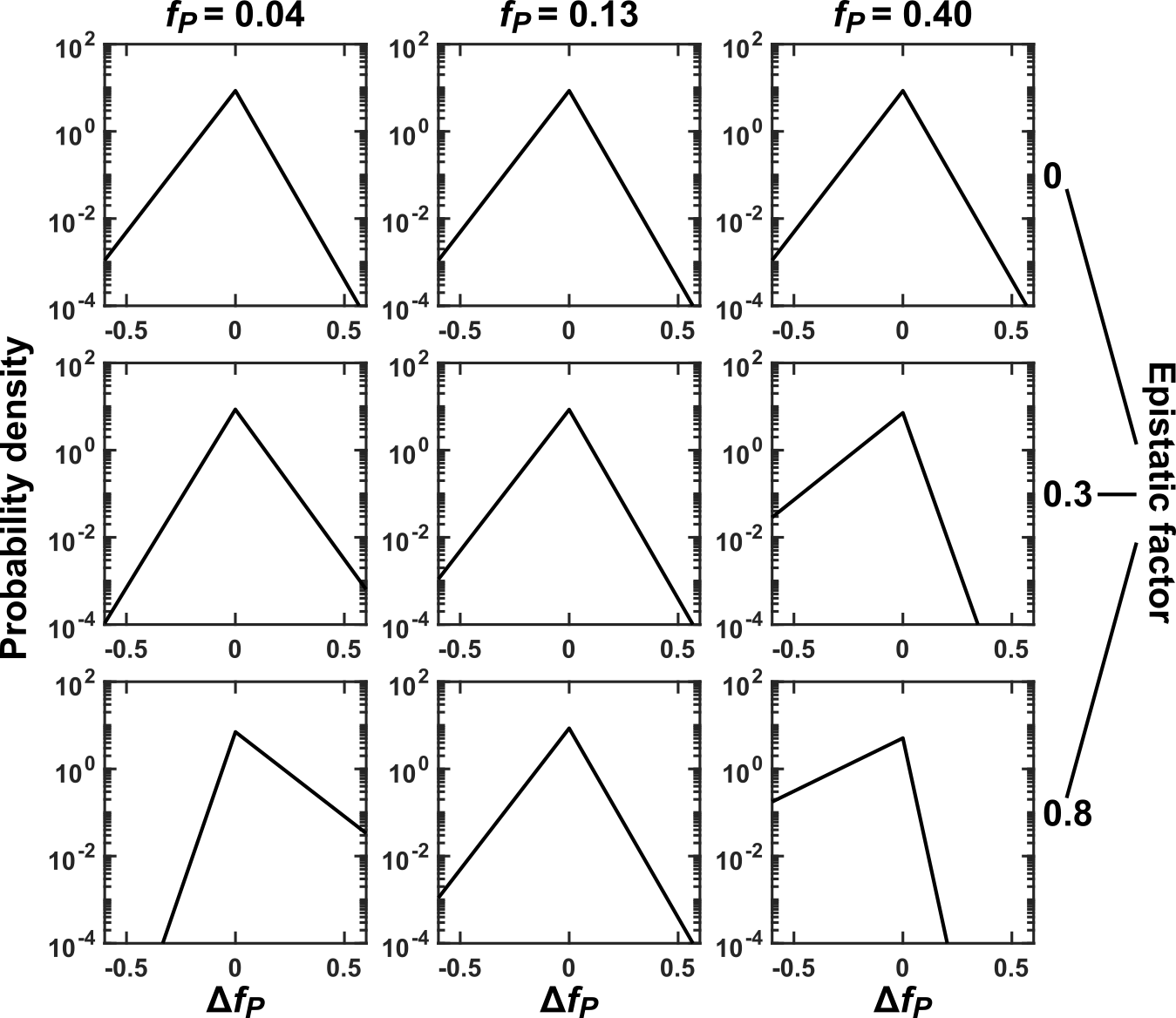

Supplement: S20 Fig — Distribution of mutation effects at different current fP values (marked on top) are plotted according to Eq 20. (Top) When there is no epistasis, distribution of mutational effects on fP (ΔfP) remains identical regardless of current fP. (Middle and Bottom) With epistasis (see Methods Section 5 for definition of epistasis factor), mutational effects on fP depend on the current value of fP. If current fP is low (left), enhancing mutations are more likely to occur (the area to the right of ΔfP = 0 becomes bigger), and their mean mutational effect (= 1/slope) becomes larger. If current fP is high (right), the opposite is true. (TIF) [file pbio.3000295.s020.tif]

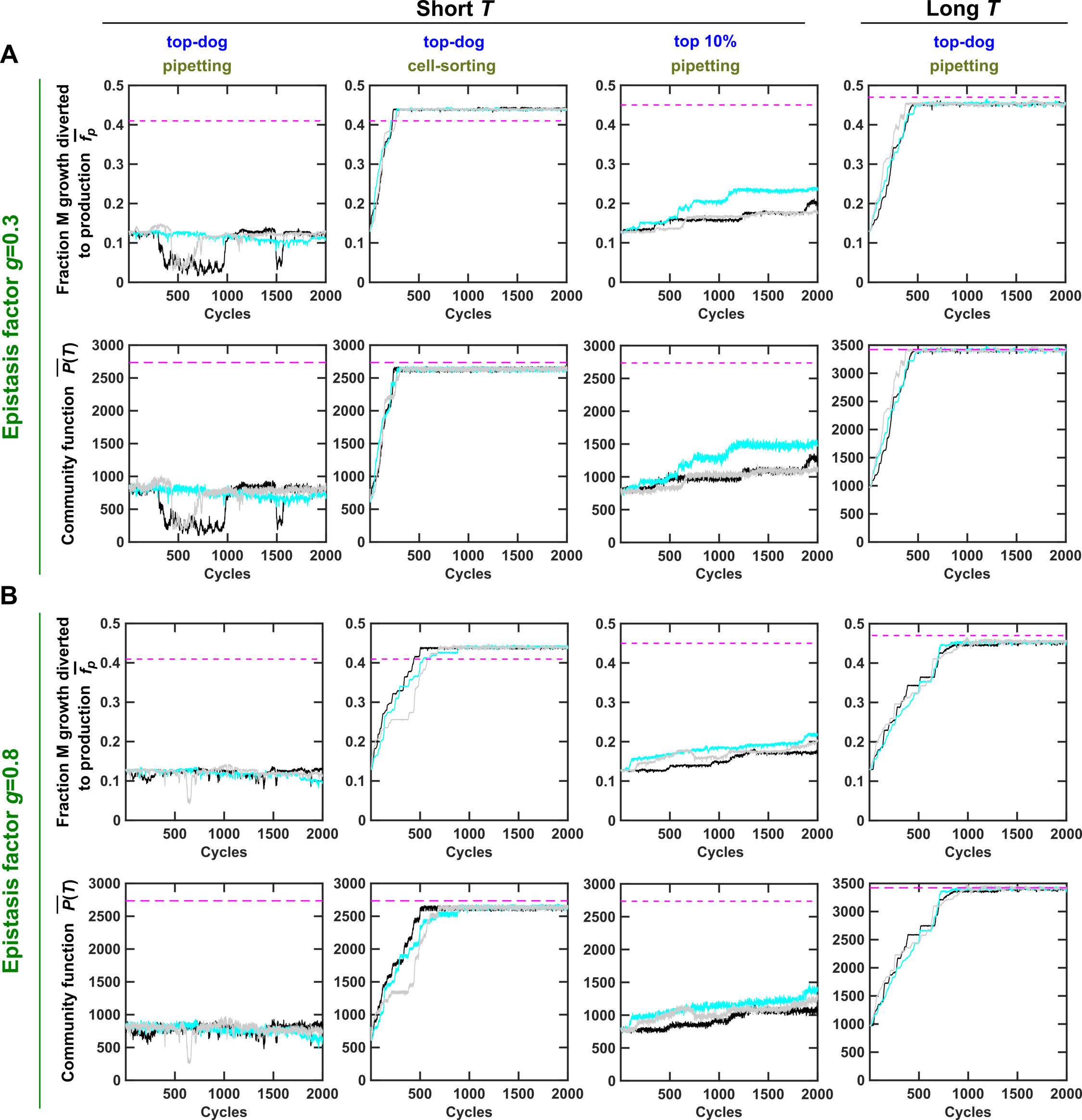

Supplement: S21 Fig — When we incorporated different epistasis strengths (epistasis factor of 0.3 and 0.8), we obtained essentially the same conclusions as when epistasis was not considered (Fig 3). Black, cyan, and gray curves are independent simulation trials. P¯(T) was averaged across the chosen Adults. f¯P(T) was obtained by first averaging among M within each chosen Adult and then averaging across all chosen Adults. For panel A, the simulation codes can be found in S16 Code, and the data can be found in S15 Data. For panel B, the simulation codes can be found in S16 Code, and the data can be found in S16 Data. (TIF) [file pbio.3000295.s021.tif]

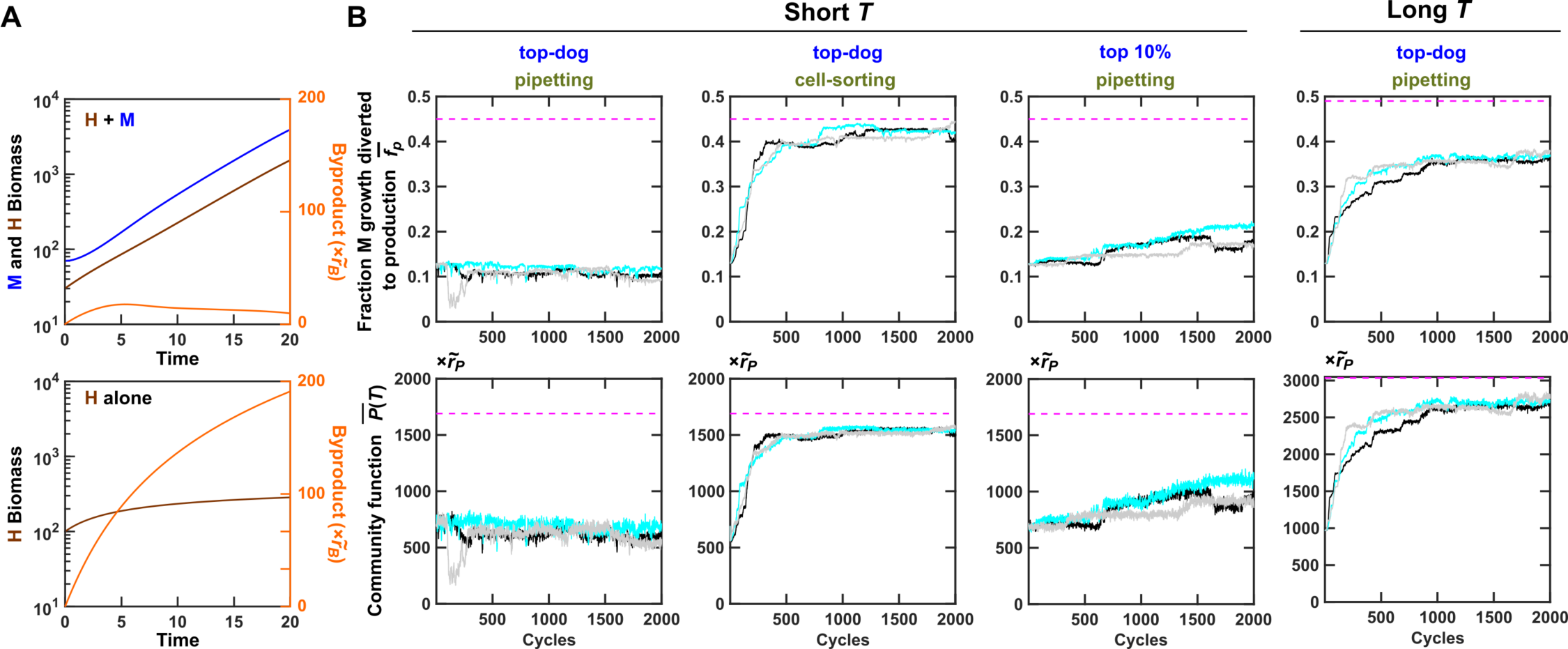

Supplement: S22 Fig — In the mutualistic H-M community, H generates Byproduct that is essential for M but inhibitory to H. (A) H can grow to a high density in the presence of M (top) but not in the absence of M (bottom). (B) Similar to community selection on commensal H-M communities, selection was promoted by the top 10% strategy or cell sorting at short T (T = 20), or via extending T (T = 24). Black, cyan, and gray curves are independent simulation trials. P¯(T) was averaged across the chosen Adults. f¯P(T) was obtained by first averaging among M within each chosen Adult and then averaging across all chosen Adults. For panel A, the Matlab codes can be found in S17 Code. For panel B, the simulation codes can be found in S18 Code, and the data can be found in S17 Data. (TIF) [file pbio.3000295.s022.tif]

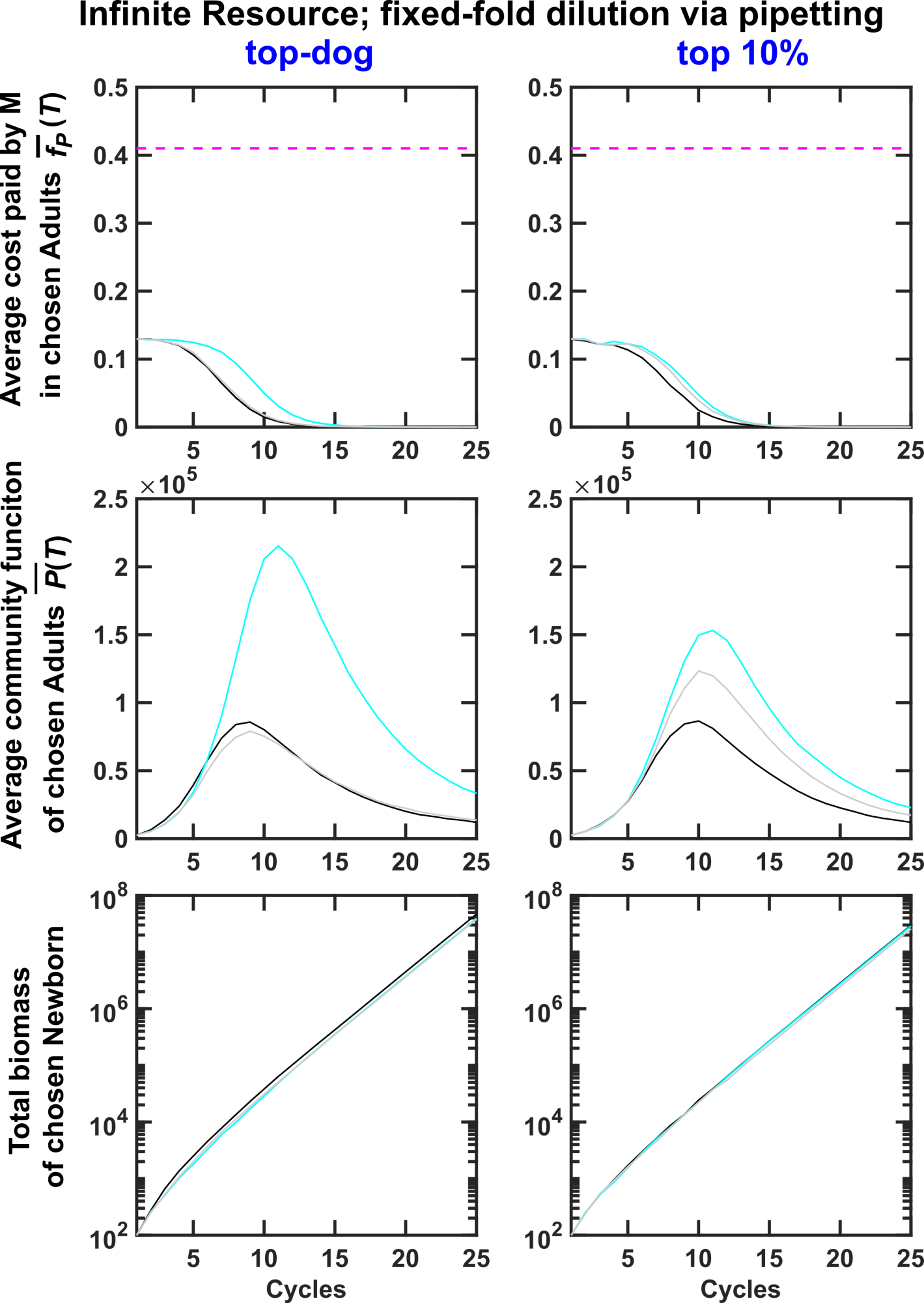

Supplement: S23 Fig — Excess Resource was supplied to each Newborn (R(0)/KMR = 106), and chosen Adults were reproduced via a fixed-fold (100-fold) pipetting dilution into Newborns. Because of pipetting, Newborns with larger total biomass will tend to be selected (Fig 4F). Community function initially increased as Newborn total biomass increased exponentially (middle and bottom panels), while nonproducing M cells with fP = 0 quickly took over (top panel; S7B Fig). Black, cyan, and gray curves are independent simulation trials. P¯(T) was averaged across chosen Adults. f¯P(T) was obtained by first averaging among M within each chosen Adult and then averaging across all chosen Adults. The simulation codes can be found in S19 Code, and the data can be found in S18 Data. (TIF) [file pbio.3000295.s023.tif]

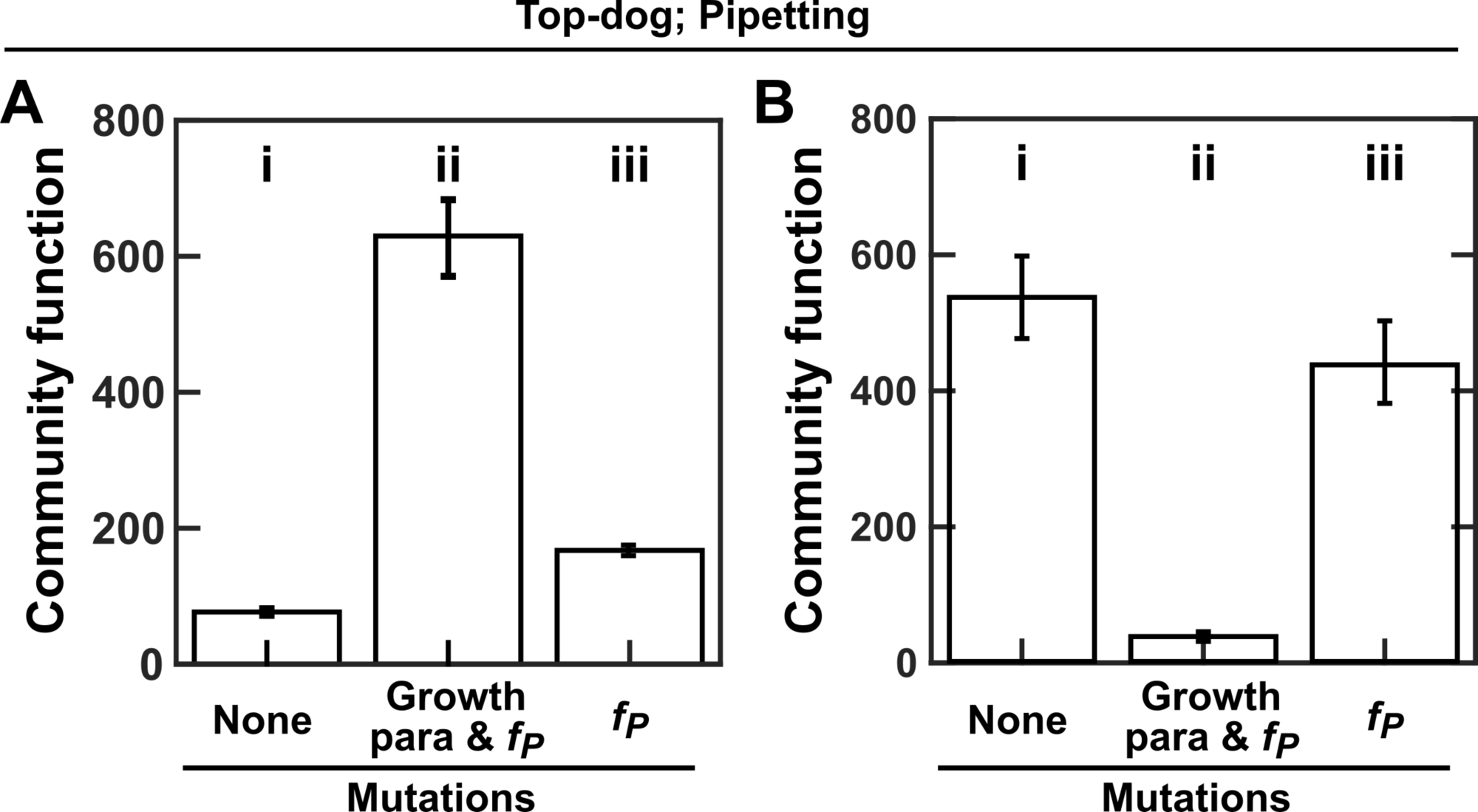

Supplement: S24 Fig — Plotted here are plateaued community function after 1,500 cycles when simulation did or did not allow mutations in growth parameters or fP. The top-dog strategy and pipetting were used. (A) When evolutionary upper bound for gHmax (gHmax*=0.3) was lower than that of gMmax (gMmax*=0.7), improving growth parameters improved community function. Compared to community function where no mutations were allowed (i), community function improved when both growth parameters and fP were allowed to mutate (ii). Preventing mutations in growth parameters diminished community function improvement (iii). In this case, improved growth of M and H resulted in higher community function. Evolutionary dynamics are shown in S8A–S8C Fig. (B) When evolutionary upper bound for gHmax (gHmax*=0.8) was larger than that of gHmax (gMmax*=0.7), improving growth parameters could decrease community function. Compared to community function in which no mutations were allowed (i), community function decreased when both growth parameters and fP were allowed to mutate (ii). Preventing mutations in growth parameters diminished reduction in community function (iii). In this case, improved growth of M and H resulted in lower community function. Evolutionary dynamics are shown in S17A Fig and Fig 6A. In panel B, Resource supplied to Newborn communities could support 105 total biomass to accommodate faster growth rate. Error bars are calculated form 3 independent selections. The simulation codes can be found in S2 and S4 Codes. The plot can be generated by S20 Code from S19 Data. (TIF) [file pbio.3000295.s024.tif]

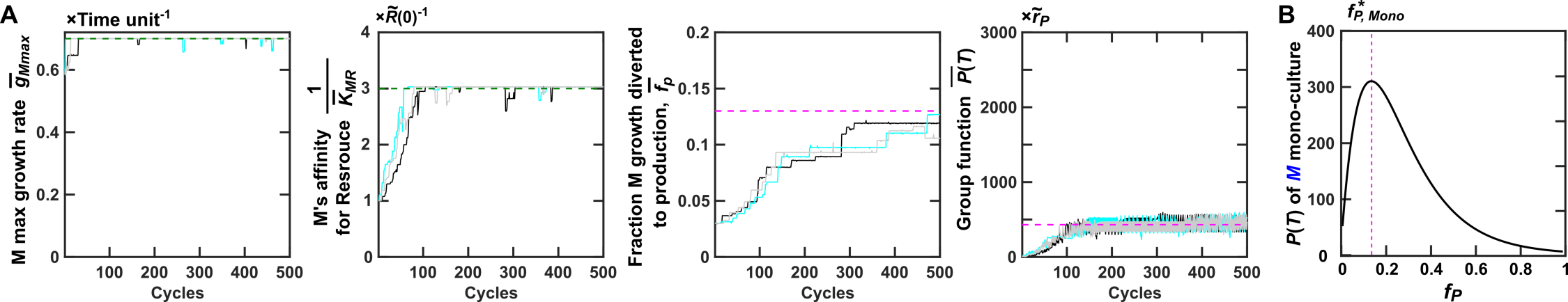

Supplement: S25 Fig — (A) Phenotypes averaged over chosen groups are plotted for 500 selection cycles. Because Resource is the same as in community selection while Byproduct is in excess, M’s affinity for Byproduct 1/KMB is no longer relevant in equations (S3B Fig, RM ≪ BM). Upper bounds of M’s maximal growth rate gMmax and M’s affinity for Resource 1/KMR are marked with green dashed lines. Magenta lines mark fP optimal for group function and maximal P(T) when M’s maximal growth rate gMmax and M’s affinity for Resource 1/KMR are fixed at their upper bounds and when Byproduct is in excess. (B) Suppose that a Newborn M group starts with a single Manufacturer (biomass 1) supplied with excess Byproduct and the same amount of Resource as in a Newborn H-M community (Resource could support 104 M biomass). Then, maximal group function is achieved at fP=fP,​Mono*=0.13 (middle panel), lower than the optimal fP for the community function fP*=0.41 (Fig 2B). Here, the growth parameters of M are all fixed at their evolutionary upper bounds, and P(T) has the unit of r~P. For panel A, the simulation codes can be found in S21 Code, and the data can be found in S20 Data. For panel B, the Matlab codes can be found in S22 Code. (TIF) [file pbio.3000295.s025.tif]

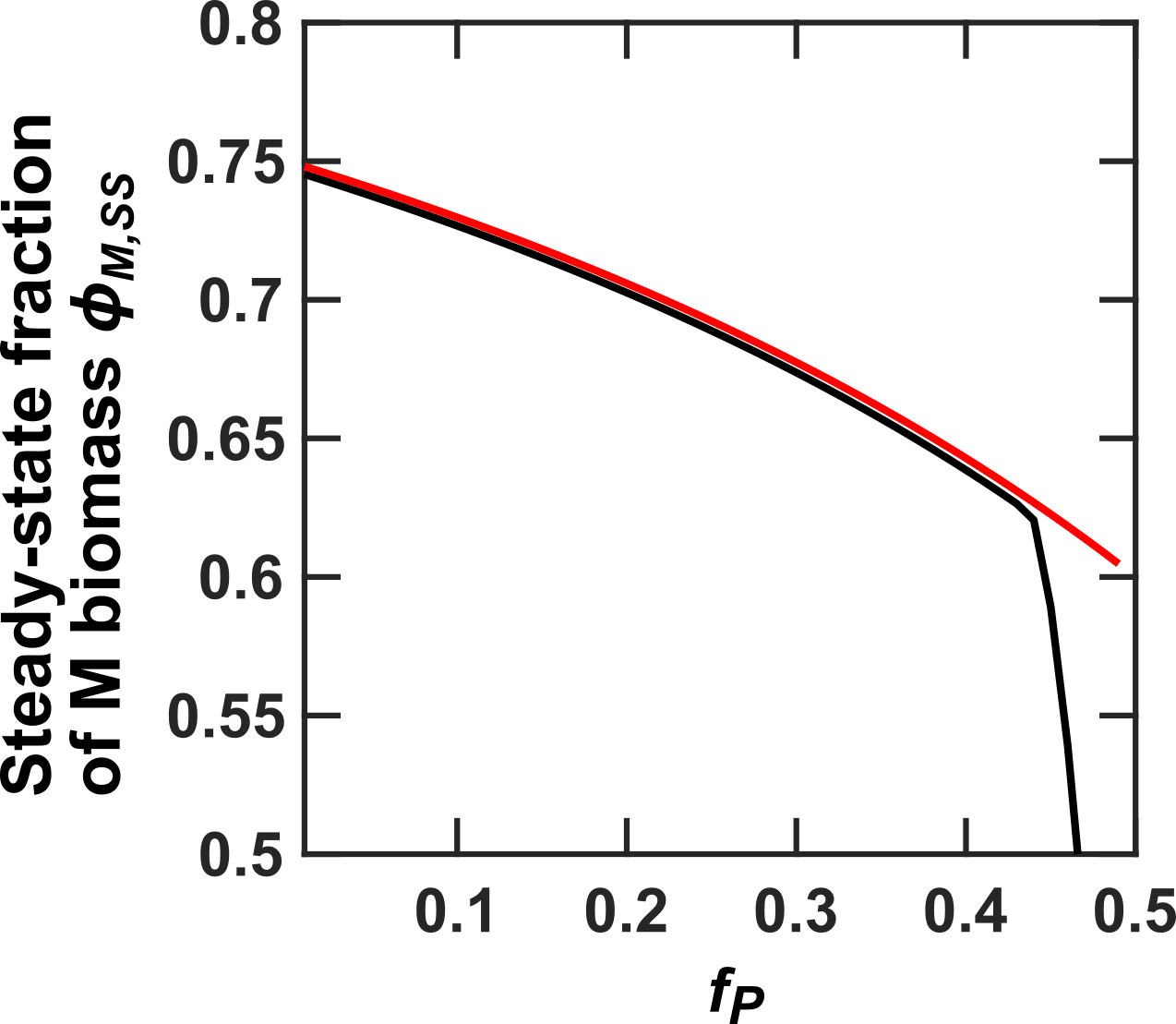

Supplement: S26 Fig — The Matlab codes can be found in S23 Code. (TIF) [file pbio.3000295.s026.tif]

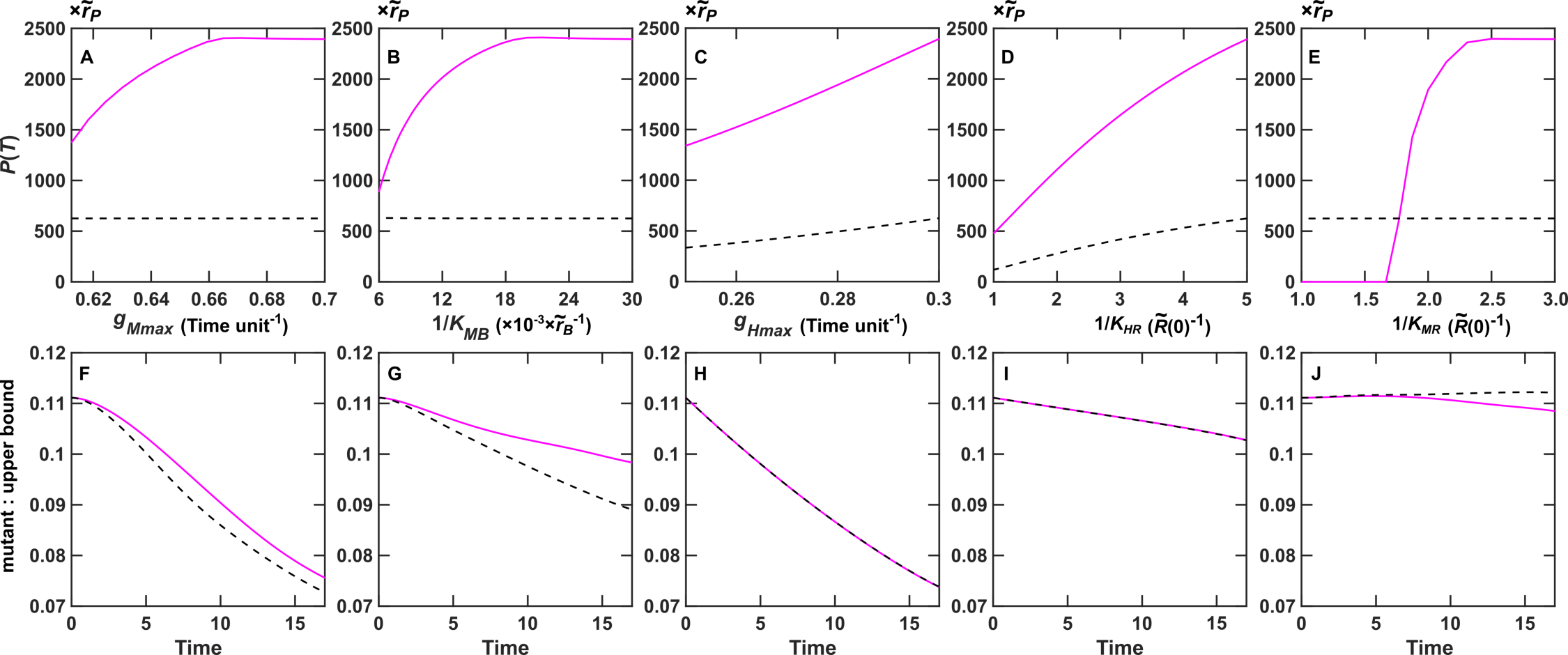

Supplement: S27 Fig — In all figures, solid and dashed lines, respectively, represent calculations with fP=fP*=0.41 (optimal for community function; Fig 2B) and fP=fP,​Mono*=0.13 (the starting point for most of our simulations; optimal for M monoculture production when Byproduct is in excess—see S25B Fig). Except for the growth parameter indicated on the horizontal axis, all other growth parameters were set to their respective upper bounds. (A–D) Community function increases as the indicated growth parameter increases. For example, in (A), all growth parameters except for gMmax were set to their upper bounds. For each gMmax, the steady-state ϕM,SS was calculated using equations in Methods Section 1. This steady-state ϕM,SS was then used to calculate P(T). (F–I) The ratio between mutant population (whose indicated growth parameter was 10% lower than the upper bound) and preadapted population (with all growth parameters at upper bounds) over maturation time T = 17. The decreasing ratio indicates that the mutant has a lower fitness compared to the growth-adapted cells. For example, in (F), a Newborn community had 70 M and 30 H. Among M cells, 90% were preadapted and had upper bound gMmax = 0.7 ("upper bound"). The remaining 10% had gMmax = 0.63, 10% less than the upper bound ("mutant"). The ratio between "mutant" and "upper bound" cells declined over maturation time, indicating that mutant M cells had a lower fitness. (E, J) When fP = 0.13 (black dashed line) but not when fP = 0.41 (magenta line), increasing M’s affinity for Resource (1/KMR) slightly decreases individual fitness and barely affects community function. The Matlab codes can be found in S24 Code. (TIF) [file pbio.3000295.s027.tif]

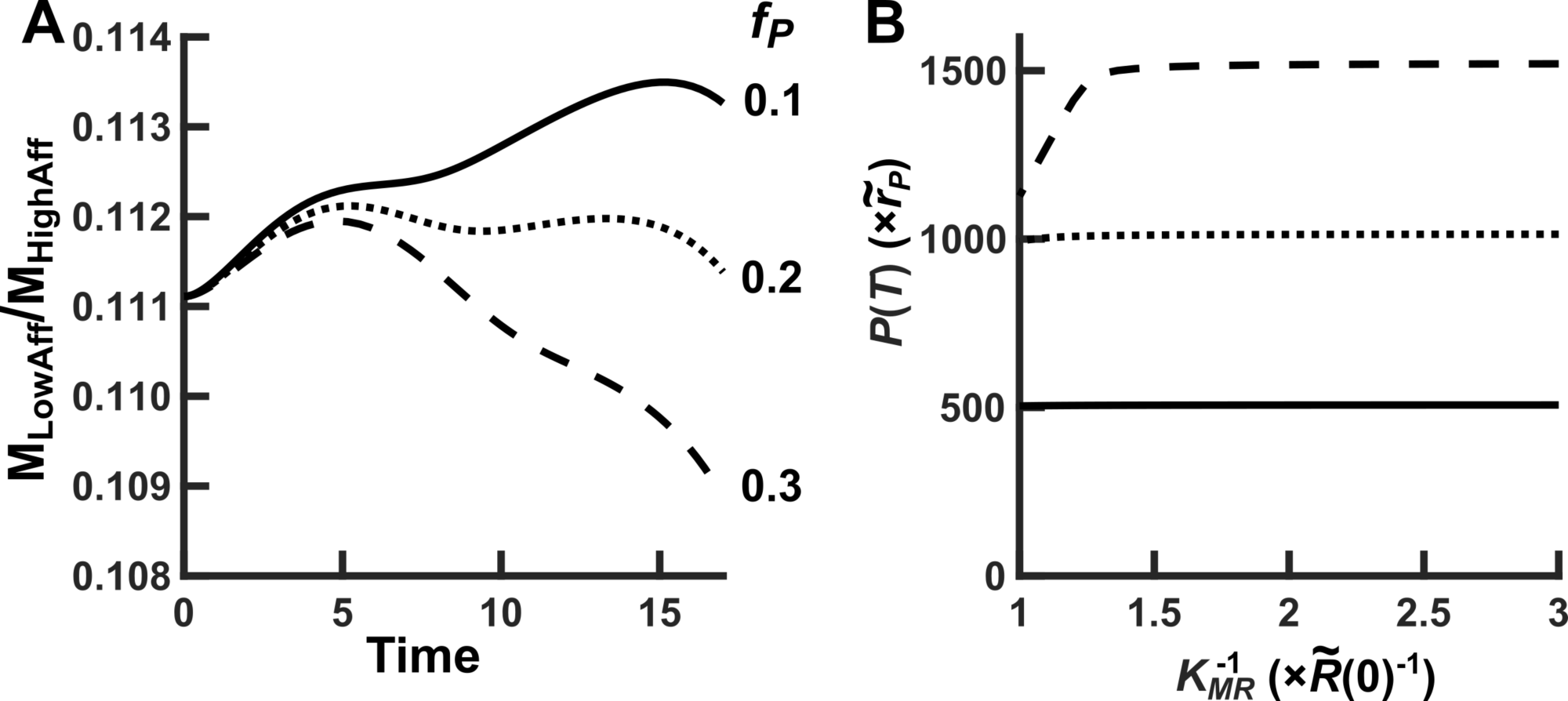

Supplement: S28 Fig — (A) The ratio between MLowAff (the population size of M with low affinity for Resource KMR-1=2.5R~(0)-1) and MHighAff (the population size of M with high affinity for Resource KMR-1=3R~(0)-1) are plotted over a maturation cycle when grown together in the H-M community. The fP values of both populations equaled to 0.1 (solid line), 0.2 (dotted line), and 0.3 (dashed line). (B) P(T) improves over increasing affinity KMR-1 when fP is 0.1 (solid line), 0.2 (dotted line), and 0.3 (dashed line). The dependence of P(T) on affinity KMR-1 is rather weak for low fP. For example, when KMR-1 increases from 1 to 3, P(T) increases by only 2% and 0.6% for fP = 0.2 and fP = 0.1, respectively. The Matlab codes can be found in S25 Code. (TIF) [file pbio.3000295.s028.tif]

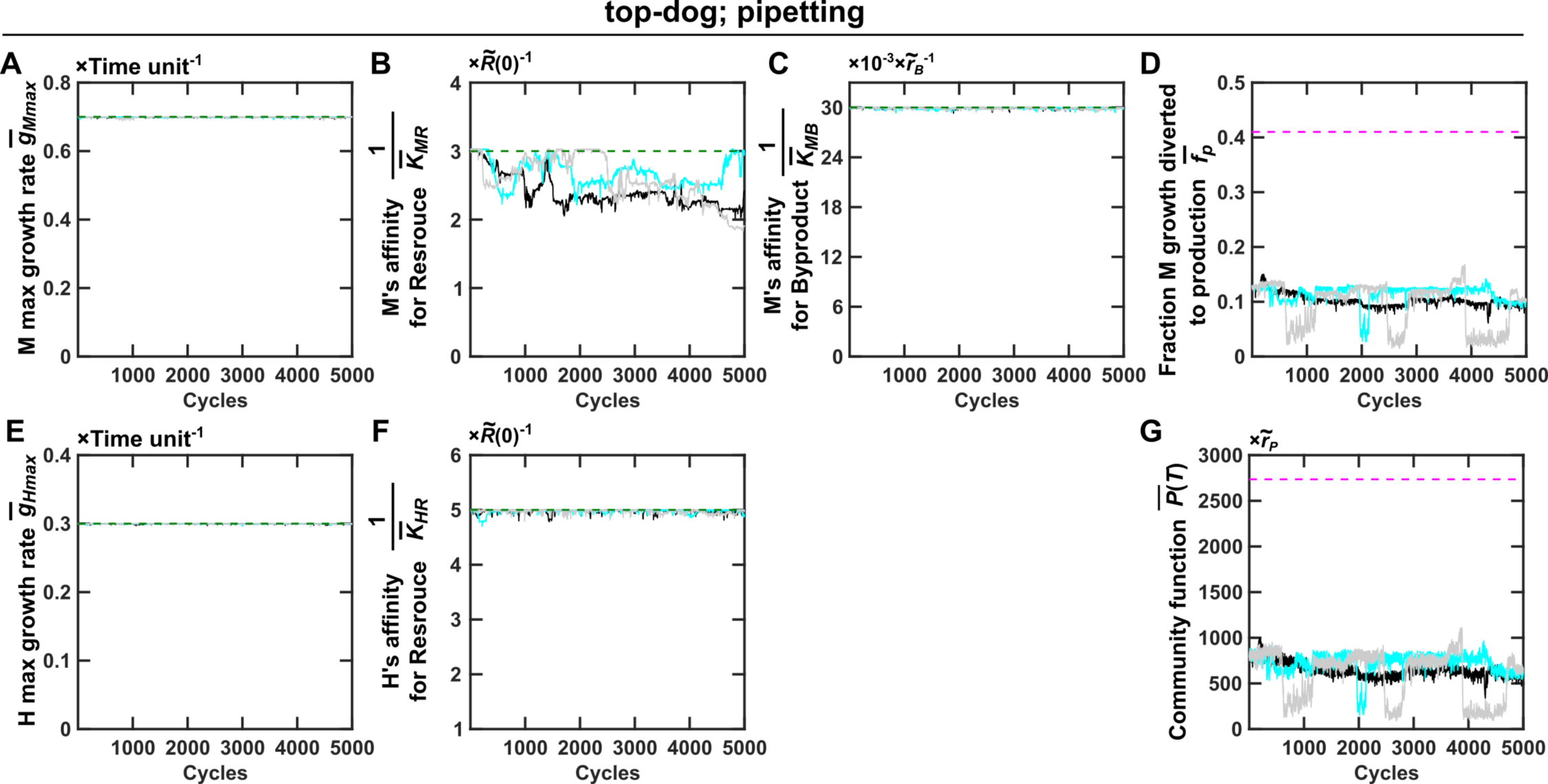

Supplement: S29 Fig — In the Newborn communities of the first cycle of community selection, all growth parameters of H and M were at their upper bounds and fP=fP,​Mono*=0.13 (S25 Fig). The top-dog strategy was used to choose Adults that were then reproduced via pipetting. When we simulated community selection while allowing all growth parameters and fP to vary, M’s affinity for R 1/K¯MR decreased slightly because at low fP = 0.13, M with a lower affinity for R (lower 1/KMR) has a slightly improved individual fitness (S28 Fig). Other growth parameters (g¯Mmax,g¯Hmax,1/K¯MB, and 1/K¯HR) remain mostly constant during community selection because mutants with lower-than-maximal values were selected against by intracommunity selection and by intercommunity selection (S27 Fig). Other legends are the same as in S8 Fig. The simulation codes can be found in S4 Code, and the data can be found in S21 Data. (TIF) [file pbio.3000295.s029.tif]

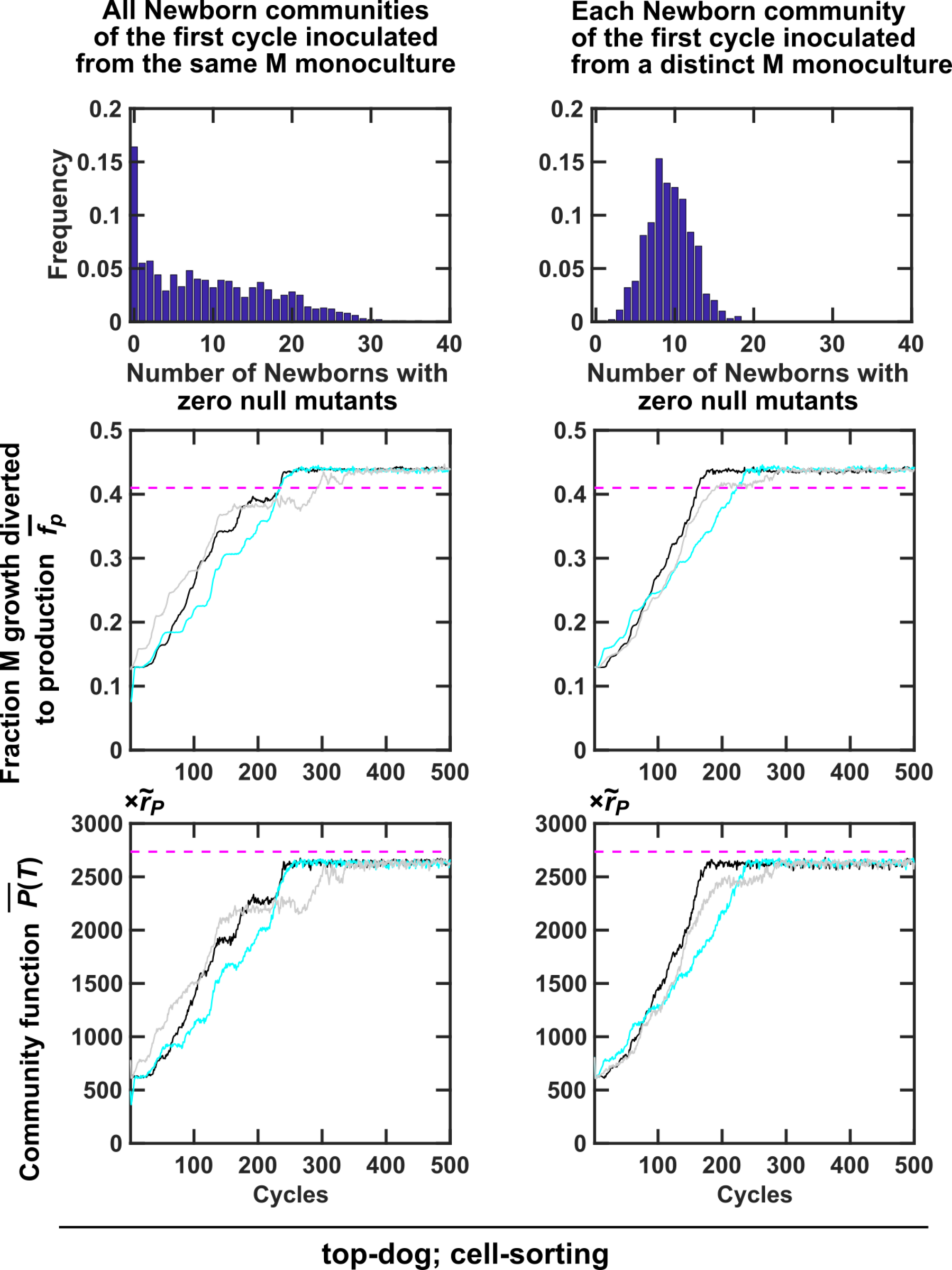

Supplement: S30 Fig — An M monoculture grew from a single non-null M cell. This M cell went through approximately 23 doublings and therefore multiplied into approximately 107 cells. Every time a non-null M cell divides, the mother and daughter cells can independently mutate and become a null M cell (fP = 0) at a fixed probability of 10−3. If a non-null M cell has fP = 0.13, then it will grow at a rate 87% of that of a null cell. After approximately 23 doublings, the M monocultures have on average about 3% null mutants. Sixty randomly chosen M cells from the same monoculture or from distinct monocultures, together with 40 H cells, were used to inoculate each of the 100 Newborns for the first selection cycle. (Top panels) Histograms of the number of Newborn communities of the first cycle that are free of noncontributor M mutants when inoculated from a single M monoculture (Left panel) or from independently grown M monocultures (right panel). To generate the histograms, the pregrowth and inoculation process was repeated 1,000 times. (Middle and bottom panels) Improvement in f¯P(T) and P¯(T) was only slightly slower when Newborn communities from the first cycle were inoculated by the same M monoculture (left panel) than by distinct monocultures (right panel). The top-dog strategy was used to choose Adults that were then reproduced via cell sorting. Black, cyan, and gray curves are independent simulation trials. P¯(T) was averaged across the 2 chosen Adults. f¯P(T) was obtained by first averaging among M within each chosen Adult and then averaging across the chosen Adults. The simulation codes for evolution dynamics can be found in S26 Code, the simulation codes for the histograms can be found in S27 Code, and the data can be found in S22 Data. (TIF) [file pbio.3000295.s030.tif]
